# An inverse correlation between structural linguistic and human genetic diversity

**DOI:** 10.1073/pnas.2526762123

**Published:** 2026-05-01

**Authors:** Anna Graff, Erik J. Ringen, Taras Zakharko, Mark Stoneking, Kentaro K. Shimizu, Balthasar Bickel, Chiara Barbieri

**Affiliations:** ^a^ Institute for the Interdisciplinary Study of Language Evolution, University of Zurich, Zurich 8050, Switzerland; ^b^ Department of Evolutionary Biology and Environmental Studies, University of Zurich, Zurich 8057, Switzerland; ^c^ Linguistic Research Infrastructure, University of Zurich, Zurich 8050, Switzerland; ^d^ Department of Evolutionary Genetics, Max Planck Institute for Evolutionary Anthropology, Leipzig 04103, Germany; ^e^Evolutionary and Computational Genomics, Biométrie et Biologie Evolutive, UMR 5558, CNRS and Université de Lyon, Villeneuve 69622, France; ^f^Kihara Institute for Biological Research, Yokohama City University, Yokohama 244-0813, Japan; ^g^ Dipartimento di Scienze della Vita e dell’Ambiente, Università degli Studi di Cagliari, Cagliari 09124, Italy

**Keywords:** language contact, population genetics, linguistic diversity

## Abstract

Linguistic structures show uneven global distributions, but it remains unknown to what extent such distributions are driven by human population history at a global scale. Here, we track population history through population genetics and show that, adjusting for geography, phylogeny, and environment, genetic diversity (in terms of local homozygosity modeled across individuals) is inversely correlated with linguistic diversity (in terms of local entropy of structural features modeled across languages). This inverse correlation arises from the parallel impact of isolation vs. contact on both genomic and structural linguistic diversity: Isolation leads to low genetic diversity and promotes structural linguistic diversification, while contact and migration yield higher genetic diversity and promote linguistic homogenization. The extent of the correlation varies across world regions and aspects of language, but its overall global robustness highlights how hotspots of linguistic diversity can serve as a compelling example of the flexibility of human language, since they have been less affected by the increase of contact and migration that occurred over recent millennia and homogenized linguistic structures.

## Significance

Languages have highly diverse structures—e.g. some place verbs first, others elsewhere—and this diversity is remarkably uneven across the globe. In some regions, languages show a variety of verb placements, while in others they share the same. By linking population genetic and linguistic data, we show that relatively isolated regions (with low levels of genetic diversity) exhibit higher degrees of structural diversity across languages, whereas regions with local histories of more contact and migration (higher genetic diversity) tend toward lower levels of structural linguistic diversity. This inverse correlation between linguistic and genetic diversity highlights that hotspots of linguistic diversity, reflective of relative isolation, are crucial windows into the flexibility of the language faculty and the dynamics of linguistic evolution.

**Fig. 1.**
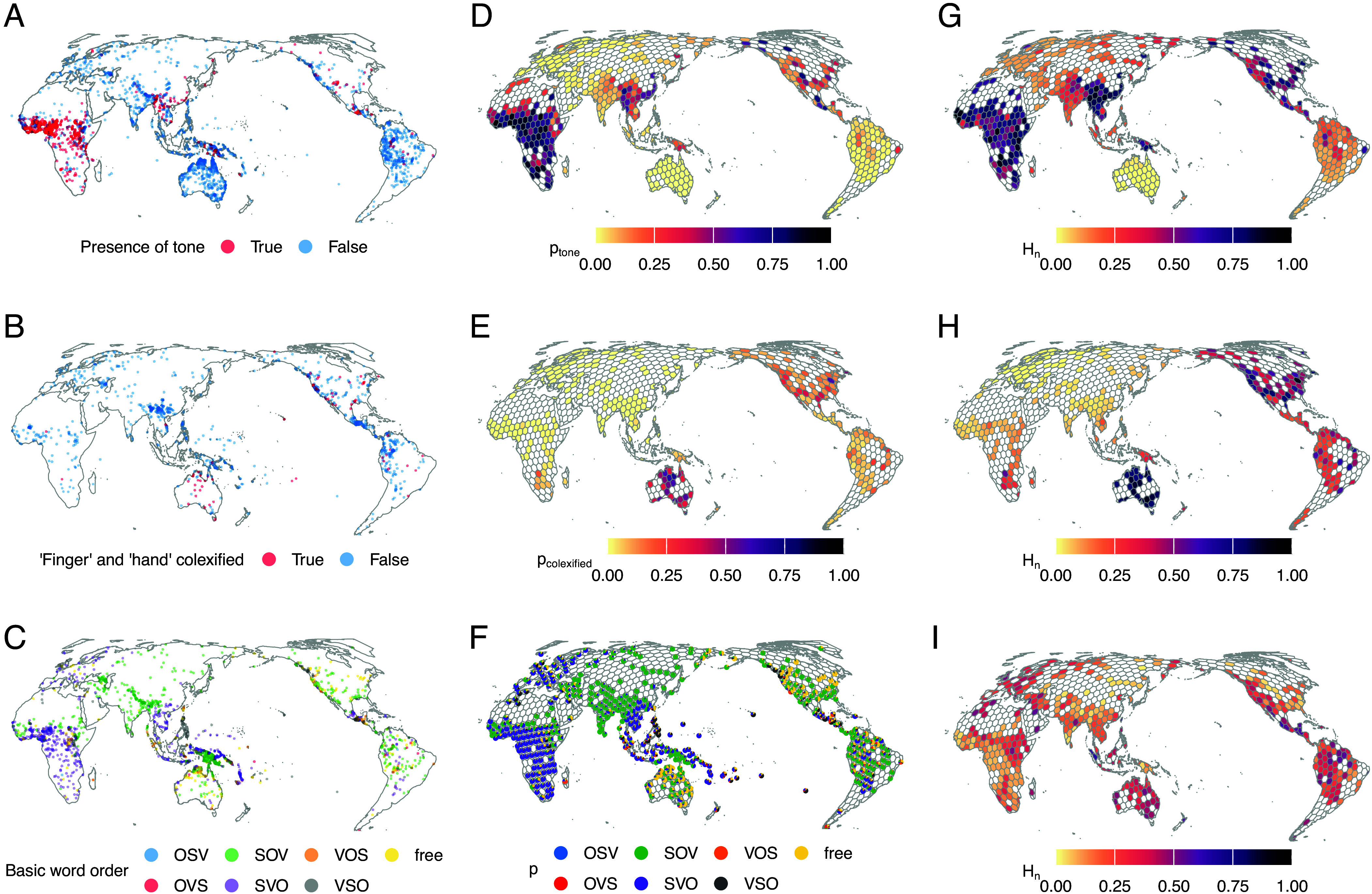
Examples of the uneven distribution of global structural diversity for three linguistic features from different linguistic domains. The *Left*-hand maps (*A*–*C*) show each coded language’s feature value according to Glottolog’s language coordinates. The maps in the middle (*D*–*F*) show posterior probabilities per grid cell for each feature state, adjusting for a global baseline, the geohistorical area the cell is located in as well as spatial and phylogenetic autocorrelation. The *Right*-hand maps (*G*–*I*) show the model-based estimates of normalized entropies (H_n_) of each feature’s distribution, derived directly from these probabilities. Grid cell diameters measure 500 km. (*A*, *D*, and *G*) Presence of tone (phonology) in N = 2,236 languages. (*B*, *E*, and *H*) Use of the same word (“colexification”) for “finger” and “hand” (lexical semantics) in N = 881 languages. (*C*, *F*, and *I*) Basic word order (S: most agent-like noun phrase, O: most patient-like noun phrase, V = verb; morphosyntax) in N = 1,502 languages. The *SI Appendix* provides the equivalent distributions obtained after randomly jittering language coordinates within a 250 km radius (sensitivity analysis; *SI Appendix*, Fig. S1), as well as zoom-in insets of high density areas: the Gulf of Guinea (*SI Appendix*, Fig. S2), and the greater New Guinea region (*SI Appendix*, Fig. S3).

The genetic patterns that emerge from human population history explain much of the distribution of languages over space and time, although not to the extent that Charles Darwin surmised in his famous proposal that the human pedigree would match the phylogeny of languages (1, 2). Much less is known about how genetic history might explain the distribution of the structures that languages have, such as patterns in their grammars, vocabularies, and sound systems (Fig. 1 and *SI Appendix*, Figs. S1–S3), which tend to be spread over larger arrays of languages and language families. For example, the linear order of sentences tends to be similar over major language families in Asia, across parts of Indo-European, Turkic, Mongolic, Tungusic, and several others (green dots in Fig. 1*C*). Early proposals that this results from population history over several millennia (3) have remained difficult to assess without sufficient evidence at this time depth. Progress in linking linguistic information to evidence about population history from genetics has now opened new avenues to address such questions. For example, after adjusting for spatial autocorrelation, genetic distances have been found to correlate with grammar but not phonology or the lexicon in Northeast Asia (4), with specialized parts of the lexicon in Central African hunter-gatherers (5), and with sound systems in Oceania but not elsewhere (6). However, it remains an open question how general such correlations are across different language structures and across varied world regions. While correlations with patterns of sound (7, 8) and meaning (9) might in rare cases be partially driven by functional relationships with genes, it is unknown whether other mechanisms beyond shared local history impact language diversity. One candidate mechanism is grounded in the dynamics of isolation and contact.

Human populations over the millennia have oscillated between contact and isolation, impacted by bouts of demic dispersals and expansions which left discernible traces in the genome (10). Contact and isolation can also directly impact linguistic structures, with isolation favoring diversification through vertical evolution and contact leading to homogenization through horizontal borrowing (11–13). Specifically, high levels of migration and wide-spread population contact are expected to result in regions of reduced diversity in structures (*spread zones)*. This perspective aligns with recent findings that diverse scenarios of population contact (as operationalized by genetic admixture) lead to universally similar rates of linguistic borrowing (13), with only few linguistic features exhibiting opposite effects of divergence under contact. In contrast, structurally diverse regions (*accumulation zones*) would emerge in areas where linguistic diversity is maintained or enriched, often in contexts of limited integration and increased isolation at the fringes of spread zones (11, 14, 15).

From this, for any sample location, we expect higher levels of genetic diversity, resulting from intensified contact between populations and by large and sustained population size, to correlate with linguistic homogenization. Conversely, we expect lower levels of genetic diversity, caused by isolation under reduced contact, a small population size and bottlenecks, to correlate with linguistic diversification. We expect this effect to be indirect, with the linguistic diversity in a given location being affected by the demographic history that took place in that same location even a few generations before.

We assess this hypothesized inverse correlation by linking global genetic data with structural linguistic data. We adjust for several confounds known to drive the distribution of languages, such as the natural environment, population densities, and local sociocultural traditions (*SI Appendix*, Fig. S4) (16–26). Most of these confounds stem from shared history although the relationship between sound systems and environmental temperature (27–29) is possibly adaptive.

Two alternative outcomes to the expected inverse correlation are possible. First, the absence of any detectable correlation would suggest that structural linguistic diversity evolves largely independently of any demographic contact patterns and is instead primarily shaped by cultural transmission or functional (e.g., cognitive) constraints. Second, a direct correlation between genetic and linguistic diversity would imply that languages diversify by drift-like processes in populations, with contact exerting little homogenizing influence.

We note that both genetic and linguistic contact can arise from a wide range of social and demographic processes (e.g., attitudes toward multilingualism, settlement patterns, network density, and duration) and they need not be bidirectional. Also, while genetic variation provides one quantifiable window into human population history, it captures only certain aspects of population-level processes and does not disentangle the full complexity of past social interactions or demographic events, such as all those cultural exchanges that do not lead to interbreeding. Therefore, our analyses focus on broad-scale correlations between recent genetic and structural linguistic diversity without inferring specific historical scenarios of contact. However, we note that if purely cultural or linguistic effects were large in determining local degrees of linguistic diversity, they would be expected to override the signal from genetics rather than strengthening them, and that cultural or genetic effects occurring at different times would similarly weaken the inverse correlation, rather than creating false positives.

## Modeling Diversity Estimates for Linguistic Features

We estimate a measure of structural linguistic and another of genetic diversity within equally sized grid cells. To do so, we partition the world’s land mass into a geodesic grid of near-uniformly spaced hexagons (30, 31). To ensure our results are not biased by the choice of grid cell sizes, we consider two grids: one with a median cell diameter of 500 km to approximate a realistic range of demographic, cultural, and linguistic contact, while ensuring adequate data coverage per cell, and a finer one with a median cell diameter of 300 km that reduces data coverage but can pick up narrower contact (*SI Appendix*, Fig. S4). In order to further account for uncertainty in the allocation of both genetic and linguistic data to the grids, we additionally perform all analyses on coordinates that are randomly jittered within a maximum radius of 250 km and 150 km for the coarser and finer grids, respectively (*SI Appendix*, Figs. S1–S5).

First, we estimate structural linguistic diversity in terms of normalized Shannon entropy for established features relating to structures in phonology, lexical semantics, and grammar (see Fig. 1 for one example each) in each cell. The features originate from four databases [the World Atlas of Language Structures (32), AUTOTYP (33), PHOIBLE (34), and Lexibank (35)] that were previously unified, standardized, and systematically curated to minimize logical and statistical independence among features (Typology Linked and Independent or “TLI-statistical” for short) (36). The dataset covers 333 features in 4,257 languages worldwide. Each language is mapped to the spatial grid based on its coordinates information from Glottolog v5.0 (37). While polygon data exist for many languages, we rely on point-based locations because genetic sample locations are available only as points and using different sampling units for linguistic and genetic data would introduce spatial inconsistencies. Aggregating both data types into the same hexagonal grid both ensures a consistent alignment of all data layers used in the analyses while avoiding assumptions about precise language or population locations or any specific correspondence between languages and populations. The robustness checks using randomly jittered points further ascertain our results are not confounded by overly precise assumptions of sampling locations or language-population combinations.

Normalized Shannon entropy per feature and cell is computed from the expected probabilities of the feature’s states among the languages present in each cell. These probabilities are in turn estimated with a series of Bayesian generalized additive mixed models (GAMMs) with Bernoulli or Categorical likelihood functions that adjust for i) universal baseline expectations of each state across all languages the feature is coded for, ii) the broader geohistorical area in which the languages are located (33), iii) the cell the language is mapped to, iv) any relatedness among languages, and v) the spatial autocorrelation between the cells (25, 38). The universal baseline accounts for the fact that local probabilities might not be sole result of the population history of interest but can also be affected by universal preferences grounded in the processing, learning, or information-theoretical balancing of specific structures (39–41). The other adjustments account for phylogenetic and spatial autocorrelation (Galton’s Problem and Tobler’s Law) that are likely to affect local history. We note that empirically, these models are driven by both global baselines and local variation, suggesting that universal preferences on local feature probabilities are unlikely to be either overestimated or underestimated (*SI Appendix*, Figs. S6–S14). With posterior draws from each GAMM, we then compute normalized entropy estimates for each feature in each cell, along with their uncertainty (*Materials and Methods*).

As a sensitivity analysis, we also derive entropy estimates according to the same procedure from an alternative linguistic dataset based on the Grambank dataset (42), “GBI-statistical”, which covers 196 features in 2,467 languages (36). However, the intersection of linguistic and genetic data in these analyses is lower than with the TLI-statistical dataset: Out of 266 cells for which genetic data is available in the grid with a 500 km cell diameter, GBI-statistical covers data in 181 cells (68%), as opposed to 217 (82%) in TLI-statistical. In the finer grid, covering genetic data in 354 cells, GBI-statistical covers linguistic data for 184 cells (52%) and TLI-statistical for 230 (65%).

**Fig. 2.**
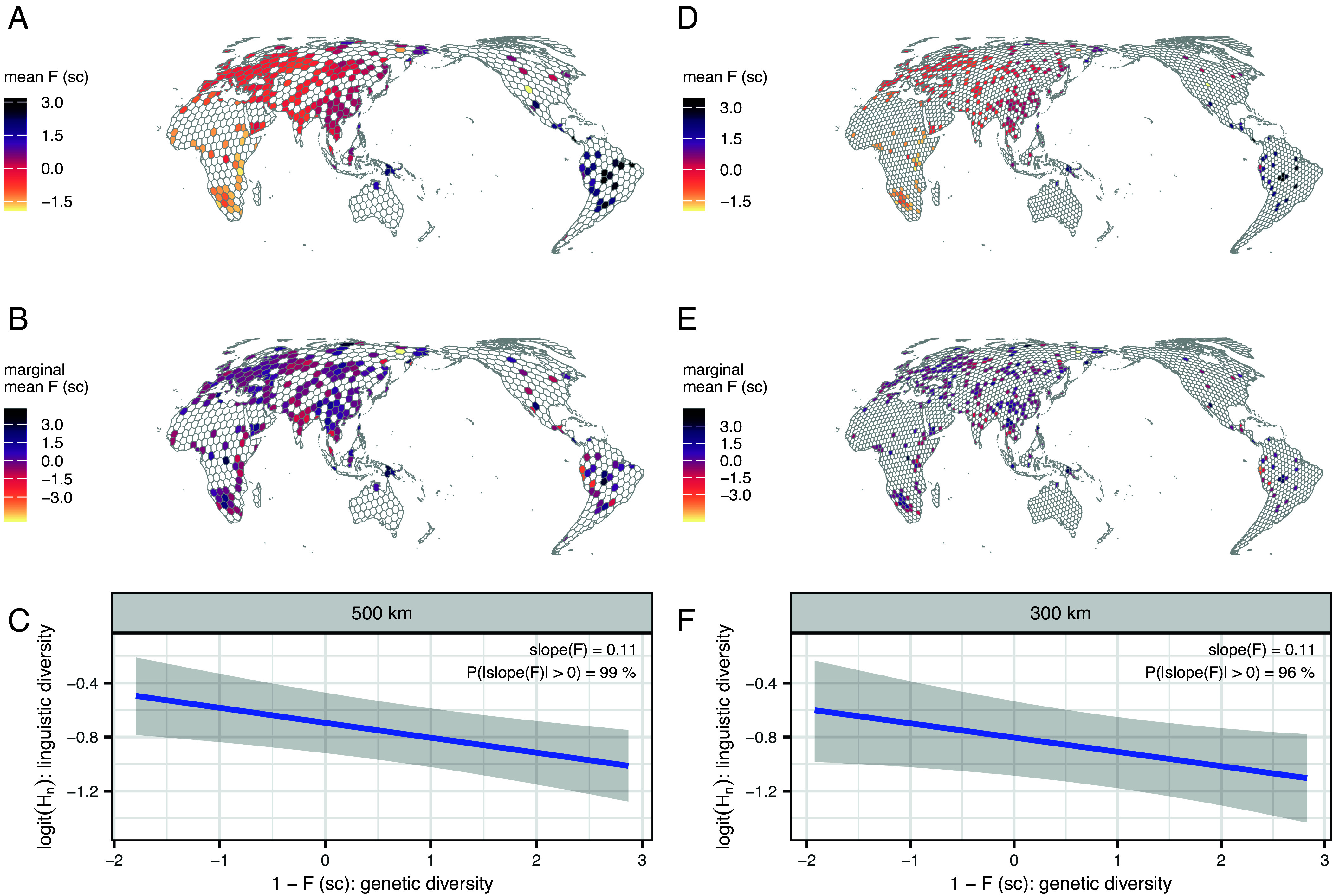
Genetic predictor: distribution, conditional distribution, and conditional effect on cell-wise linguistic diversity (H_n_, normalized entropy) when the other predictors are at their mean in the best-fitting model. (*A*–*C*) consider the grid with a mean diameter of 500 km. (*D*–*F*). consider the grid with a mean diameter of 300 km. (*A* and *D*) show the mean posterior estimates of the scaled (z-scored) Wright’s *F* coefficients across grid cells, taking into account geohistorical area, population, grid cell, and spatial autocorrelation, with higher values indicating more excess homozygosity, hence lower genetic diversity. See *SI Appendix*, Fig. S5 for the distribution of individuals, the randomly jittered distributions of individuals as well as corresponding estimates of *F*. (*B* and *E*) show scaled mean posterior *F*-values marginalized over the variation by continent, showing that random slopes by geohistorical area effectively absorb the out-of-Africa gradient. (*C* and *F*) show the conditional effect of genetic diversity on linguistic diversity from the main model. For interpretability, the x-axis is plotted on the transformed scale **1–*scaled *F* (*F**_sc_), so that higher values indicate higher genetic diversity. Gray shading represents the 89% credible intervals.

Second, we estimate genetic diversity per cell from a model which infers the degree of excess homozygosity across individuals, i.e. Wright’s *F* coefficient (or fixation index), with PLINK v1.9 (43) (*Materials and Methods*). High values of *F* correspond to low genetic diversity (Fig. 2). Values for modeling are drawn from the genetic data in the GeLaTo database, which compiles global genomic datasets generated with the Human Origins SNP chip, an array designed to minimize ascertainment bias in global analyses of human genetic diversity (1). The data cover 5,737 unrelated individuals from 650 populations, each linked to the sampling location’s geographic coordinates and derived from published studies that focused on reconstructing global patterns in human demographic history (*SI Appendix*, Fig. S5). Cell-wise estimates for *F* (*z*-scored posterior means and SD) are estimated from a GAMM with a Gaussian likelihood adjusting for i) universal baseline expectations for *F* across all individuals, ii) the broader geohistorical area in which the individual was sampled, iii) the cell the individual is mapped to, iv) the autochthonous population the individual belongs to, and v) the spatial autocorrelation between cells. Adjusting for geographical area (via random effects) effectively absorbs the out-of-Africa gradient (Figs. 2 *B* and *E*), by which values of homozygosity increase proportionally to the distance from Africa (44). Therefore, area-level random effects suffice to account for these known cross-area effects, and we focused on the scaled *F* measure as appropriate to use across our models. Because genetic sampling is globally uneven, particularly as compared to linguistic documentation, the availability and spatial distribution of genetic samples constitute the main limiting factor for how many grid cells can be included in our analyses and for the geographic match between linguistic and genetic coverage (Figs. 2 and *SI Appendix*, Figs. S4 and S5).

We then regress the (logit-transformed) entropy estimates of each feature on the estimates of the *F* coefficients (with their SD as measurement errors) in a Bayesian GAMM with a Gaussian likelihood (*Materials and Methods* and *SI Appendix*, Fig. S15 for posterior predictive checks on all models) in every cell where data for the feature and the genetics were both available. We let the effects of *F* vary across features (random intercepts and slopes) in order to profit from partial pooling, while accounting for the fact that they are coded unevenly across cells and might relate to *F* in different ways (Fig. 1 *D*–*I*). We also let the effects vary across geohistorical areas to allow for socioculturally conditioned variation at a larger scale than the grid cells and to adjust for regional differences in the scale of *F* (45) (*Materials and Methods*).

We furthermore adjust our estimates for various potentially confounding predictors (*SI Appendix*, Fig. S4 *E*–*N*). The first two are based on the notion that feature diversity might trivially just depend on the number of languages (log language richness in *SI Appendix*, Fig. S4 *E* and *F*) and the diversity of language families (log linguistic taxonomic diversity *SI Appendix*, Fig. S4 *G* and *H*) in a cell. Including these predictors directly adjusts for uneven linguistic sampling intensity across regions, so that areas with exceedingly high or low linguistic density do not bias the estimated relationships. Two further predictors, the first two principal components of a PCA over eleven measures including climatic, altimetric, productivity, and land use factors (*SI Appendix*, Fig. S4 *I*–*L*) account for environmental variation which is known to affect linguistic distributions (20, 21, 24, 25). PC1 is mainly loaded by variables related to climate, and PC2 is mainly loaded by variables related to terrain. Finally, we include (log) population density (*SI Appendix*, Fig. S4 *M* and *N*) from historical estimates as an alternative measure of population history that might affect linguistic diversity through varying intensity of contact. This predictor accounts for large-scale demographic concentration patterns, further ensuring that results are not confounded by uneven settlement density. We further include adjustments over the geographic location of each grid cell to control for spatial autocorrelation between the cells.

Our null model m1 considers only log language richness and log linguistic taxonomic diversity as (potentially trivial) predictors. Models m2-m4 further include both environmental predictors (m2); log population density (m3); or the *F* estimates for each grid cell (m4). Models m5-m7 further include additive pair-wise combinations of these predictors, and the full model m8 includes all of them. Approximate leave-one-out cross-validation (46) shows that across conditions, the full model m8 including all predictors strongly outperforms all other models (all ∆_elpd_ > 144 ± 14, see *SI Appendix*, Fig. S16). In the robustness check using jittered coordinates, m8 also outperforms all other models, albeit m6 (including the null model predictors, plus additionally both environmental and the genetic predictors) performs similarly in the analysis using the TLI data and the coarse grid and in the analysis using the GBI data and the fine grid (*SI Appendix*, Fig. S16).

## Genetic and Geographical Isolation Predict High Structural Diversity in Languages

High estimated *F* values, i.e. low individual-level genetic diversity, are the strongest and most unambiguous predictor of high structural linguistic diversity (posterior probability of a positive effect is 99% in the 500 km diameter grid and 96% in the finer grid; Fig. 2). This means that, when considering the 500 km diameter grid, an increase in the (scaled) *F*-predictor by one SD (roughly corresponding to moving from a cell in coastal Tanzania to one in coastal Yemen) is associated with a median 0.11 increase in the logit-transformed normalized Shannon entropy (denoting structural linguistic diversity), roughly corresponding to an increase in normalized Shannon entropy by 2.3% (89% highest posterior density interval (HPDI) = (0.6%, 4.2%), when all other predictors are held at their mean. To put this into perspective, the median baseline expectation at the means of all predictors, averaged over geographic coordinates and excluding by-area, by-feature, and by-cell variation is 29.1% [89%-HPDI = (24.7%, 33.3%)]. Another way to interpret this magnitude is that, if structural diversity is computed across 333 binary features (the theoretical maximum for an analysis with the TLI dataset), a 2.3% increase in normalized entropy corresponds roughly to making 11 features maximally variable (probability 50%, high entropy) rather than having a rare state with a probability of about 5% (low entropy). In the finer grid, the median increase in logit-transformed normalized Shannon entropy corresponds to an increase in normalized Shannon entropy by 2.1% [89%-HPDI = (0.1%, 4.4%); median baseline expectation: 27.1%, 89%-HPDI = (22.2%, 32.0%)]. The result is also apparent in the sensitivity analysis using the GBI-statistical dataset, albeit with less posterior certainty, consistent with reduced data coverage (*SI Appendix*, Fig. S17). Low genetic diversity (i.e., high *F* values) is the result of small effective population size, isolation, and lack of gene-flow between groups. Our results show that these demographic factors promote diversification and/or impede homogenization in language, consistent with the hypothesized inverse correlation.

While compared to the baseline expectations, the effect seems small, it is strikingly persistent and well evidenced: Among all predictors included, including the seemingly trivial predictor of language richness and established predictors like the environmental variables and spatial autocorrelation (20–22, 24, 25), the genetic predictor emerges as the strongest and best supported across geographical resolutions and datasets in all models considering the original coordinates (*SI Appendix*, Fig. S17). In the models considering jittered coordinates, a positive slope for the genetic predictor is supported with ≥94% posterior probability for the TLI dataset. Only in the sensitivity analysis with the GBI dataset and jittered coordinates is this result less well supported, in line with the reduced power of that dataset (82% posterior probability), especially when intersected with the finer grid (62%) (*SI Appendix*, Fig. S17). This result suggests that local population history, as estimated through genetics, is an important factor in shaping the landscapes of structural linguistic diversity. We emphasize, however, that the relationship is correlational, not deterministic, as it comes from the independent effect of local demographic history on both genetic and linguistic diversity patterns. Its effect size leaves ample room for other potential factors and for stochastic variation to explain variation in local levels of structural linguistic diversity.

**Fig. 3.**
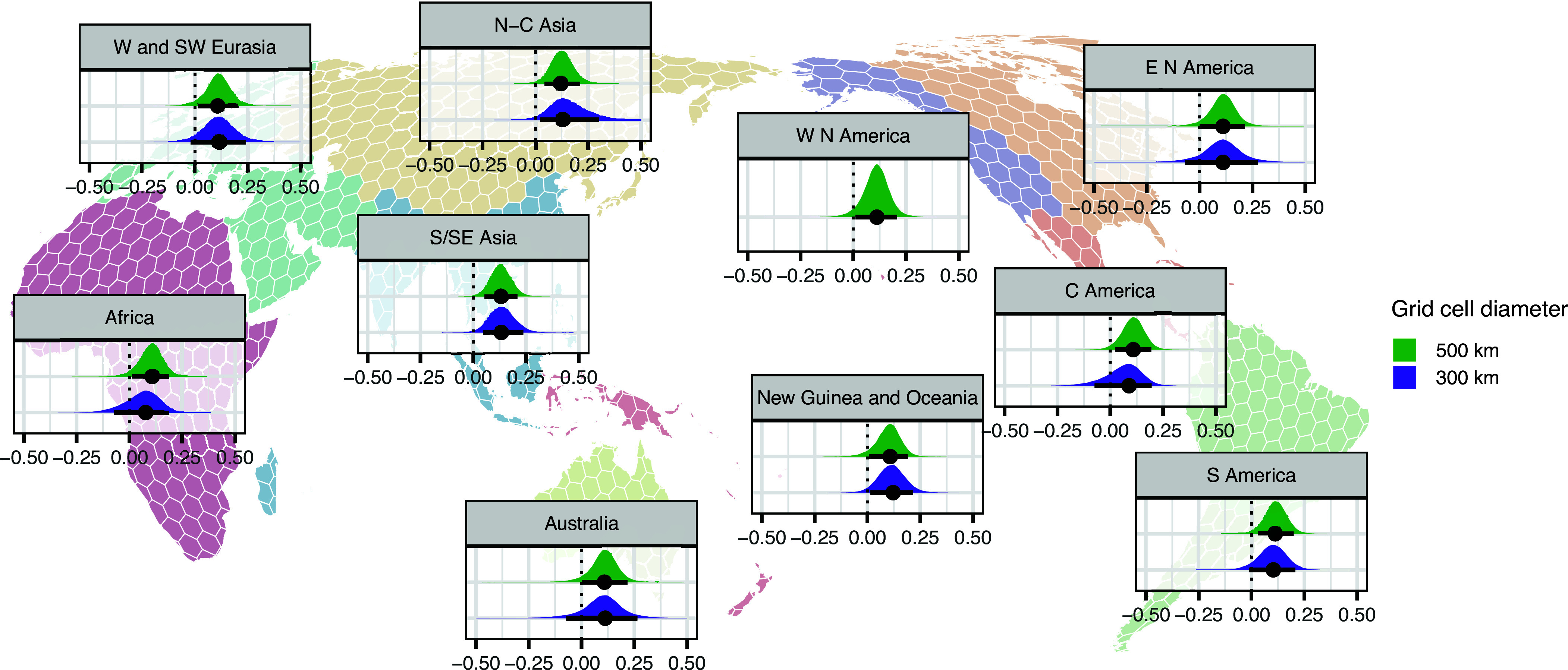
Group-level marginal effects (slopes) of the genetic effect (scaled *F*) at each geographical resolution by geohistorical area as identified by AUTOTYP. The interval bars correspond to the 89%-HPDI. Note that the 300 km grid lacks cells with both genetic and linguistic data in W N America.

The effect is similar in all ten geohistorical areas under the coarser grid resolution (with all effects > 0.10 on the logit scale and P(slope > 0) > 93%) but shows less support in some areas under the finer grid resolution and in the analyses with jittered coordinates (with effects ranging from 0.04 and 0.16 and posterior support from 78% to 99%; Figs. 3 and *SI Appendix*, Figs. S18–S21 and Table S1). Support is particularly strong under both resolutions and datasets and in the analyses with jittered coordinates in North-Central Asia as well as South and Southeast Asia [effects > 0.10, with P(slope > 0) ≥ 90%]. Support is slightly weakened again in the analyses with the finer grid when using jittered coordinates [N-C Asia: effect = 0.07 and 0.06 with P(slope > 0) = 91% and 80% for the main dataset and the sensitivity analysis, respectively; S/SE Asia: effect = 0.07 and 0.04 with P(slope > 0) = 96% and 79% for the main dataset and the sensitivity analysis, respectively; *SI Appendix*, Figs. S20 and S21 and Table S1]. A possible explanation of local increases in effect size in the Asian areas might be active processes of linguistic divergence and cultural compartmentalization, i.e. of strengthening boundaries between groups (11, 14, 47–54), but denser global data coverage is needed to test this possibility against explanations from sampling differences or alternative demographic processes.

The other predictors yield less consistent results across geographical resolutions and datasets. Language richness enhances structural diversity with less posterior support than genetic diversity (up to 99% in the analyses using the original coordinates but down to 68% in the analyses with jittered coordinates), but this result is not robust in the sensitivity analysis using the GBI dataset (*SI Appendix*, Fig. S17). Taxonomic diversity shows an effect in the opposite direction—a decrease in structural diversity—but it is supported only under the coarser [P(slope > 0) = 96%] and not under the finer resolution or sensitivity analyses (*SI Appendix*, Fig. S17). If the contrast in effect direction were to be corroborated in future research with larger and denser sampling, it would be consistent with previous observations that structural diversification is more relevant among speakers of related languages (i.e. in locations of high language richness and low taxonomic diversity) because they share common ethnolinguistic histories and may therefore be at stronger need to mark social differences (55) through schismogenesis (56, 57) than more distantly related neighbors (49, 53, 58).

Past studies highlighted that group size, population density, climate, and mountainous terrain each have a relevant but differential impact on language richness (20–22, 24, 25). Our results for these predictors are not robust across all analyses. While they generally favor the notion that higher population densities—associated with more local contact—decrease diversity in language structure, the effect has a lower posterior probability than those observed for genetics (only ≥76% posterior probability) and it is not consistent across sensitivity analyses (*SI Appendix*, Fig. S17). Similarly, regarding the environmental variables loading PC1, our results provide no support for factors associated with warm climates to favor structural diversification (only ≥60% posterior probability, *SI Appendix*, Fig. S17). For increases in environmental PC2—mainly loaded by variables indicative of mountainous terrain, which has sometimes been proposed to impede cultural and genetic contact and to harbor linguistically “conservative” and highly diverse regions (59)—we find increases of structural diversity but again with a low posterior probability of ≥61% (*SI Appendix*, Fig. S17). More extensive datasets—particularly with respect to genetics—could resolve the currently inconclusive effect directions, sizes, and support of these predictors. The fact that models excluding the environmental PCs and population density as predictors yield less predictive accuracy than the full model suggests that they cannot be dismissed as drivers of structural diversity.

## Effects on Linguistic Diversity Vary By Feature

**Fig. 4.**
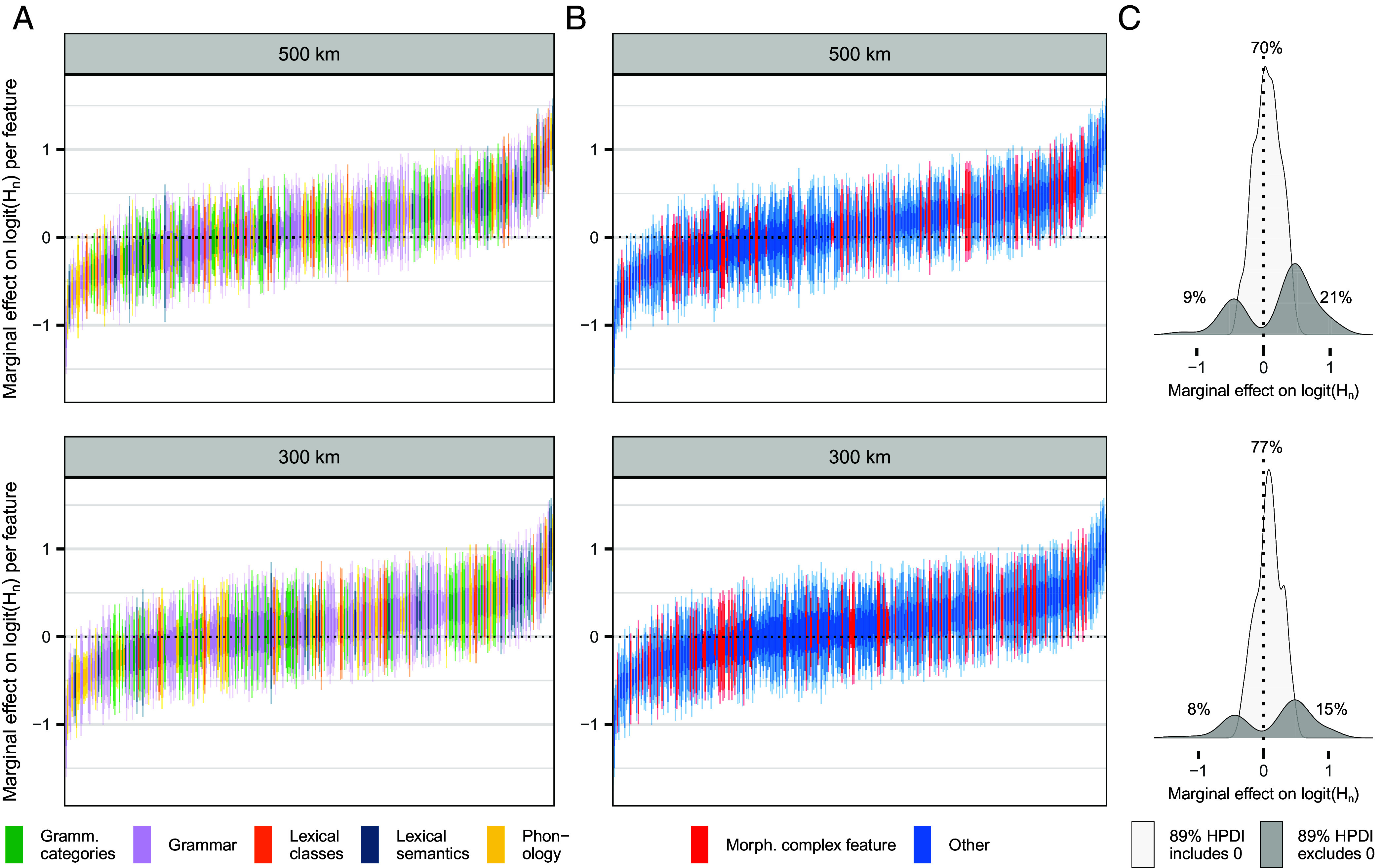
Group-level marginal effects (slopes) of the genetic effect (scaled *F*) at each geographical resolution by feature. (*A* and *B*) Features are ordered on the x-axis by their effect sizes, with intervals representing the highest posterior density intervals of 50%, 89%, and 95%. The colors show that neither domains of language (*A*, as predefined in the TLI dataset) (36) nor differences in morphological complexity (*B*, in particular, synthesis and the degree of fusion and/or affixation) (36, 63, 71) cluster in terms of marginal effects, challenging received notions of how these respond to contact and isolation between populations (See *SI Appendix*, Table S2 for a detailed list of features and their marginal effects). (*C*) Density of features with the 89% HPDI excluding vs. including zero for the genetic predictor at each resolution. The effects of genetic diversity manifest themselves in 15 to 21% of the features. Under partial pooling, some features diverge from the overall trend, exhibiting effects in the opposite direction.

While genetic diversity *F* has the largest effect size and support, these vary substantially across features (Fig. 4 and *SI Appendix*, Table S2). With the grid with a diameter of 500 km, 21% of features have a positive HPDI of 89% that excludes zero for *F* (9% of features have negative effects and 70% of features are not affected as they include zero in their 89%-HPDI). With the finer grid, these proportions lie at 15%, 8%, and 77%, respectively. In the sensitivity analyses, they lie at 19 to 35%, 13 to 25%, and 45 to 68% (*SI Appendix*, Figs. S22–S24). This substantial feature-level variation echoes recent findings of strongly varying contact effects across structural linguistic features (13). Further research is needed to probe potential factors that explain this variation, but we note that there is no apparent correlation with domains of language such as grammar, phonology, and lexical semantics (Fig. 4*A* and *SI Appendix*, Figs. S22–24*A*). Again consistent with the finding from contact effects (13), this challenges received scholarship which would lead one to expect, for example, a stronger effect of contact (i.e., low genetic diversity) reducing diversity in phonology than in grammar (60). Our findings are also inconsistent with the notion that contact and high population density reduce diversity particularly in features of complex word formation (Figs. 4*B* and *SI Appendix*, Figs. S22–24*B*) because these are difficult for second language learners (6, 61–68). Our results are more consistent with other recent findings that the effects of contact are less sensitive to degrees of complexity (69–73). Resolving these conflicting results will require targeted research on the psycholinguistic and sociolinguistic mechanisms of borrowing under different contact situations.

## Structural Diversity Hotspots As Potential Gateways to Past Linguistic Landscapes

Our finding that contact-ridden regions—those associated with high genetic diversity—exhibit low levels of linguistic diversity is consistent with the notion of spread zones (11, 14, 15), regions prone to demic and/or cultural spreads of language families associated with the emergence of agriculture and, more recently, imperial or colonial expansions. These contrast with accumulation zones, i.e., hotspots of linguistic diversity, which are associated with low genetic diversity (excess homozygosity) and, therefore, demographic histories with relatively limited and/or different patterns of contact. Languages in such hotspots of course have evolutionary histories like any other language, but our findings suggest that they were less impacted by linguistic contact effects in comparison to spread zones. As a result, they may reveal broader insights into linguistic diversity than spread zones, offering key probes for determining the boundaries of the language faculty, a long-standing challenge (74, 75). In addition, hotspots shed light on the dynamics of linguistic evolution before the large Neolithic turnovers, when smaller population densities and higher inbreeding coefficients (76–79) suggest fewer opportunities for massive contact and homogenization.

Thus, hotspots of diversity offer a privileged window on the language faculty at the level of the human species, adjusted for the confounding effects of recent population spreads. Genetic evidence is key to identify these hotspots and provides new opportunities for the language sciences—which need to move fast given the rapid global decline of language diversity (42, 80).

## Materials and Methods

### Features, Grids, and Diversity Estimates.

Linguistic features were drawn from two datasets: TLI-statistical (333 features in 4,257 languages overall) and, for a sensitivity analysis, GBI-statistical (196 features in 2,467 languages overall) (36). These datasets were curated in previous work to reduce logical and statistical dependencies between features within each dataset.

We used the approach formalized by Rivière (30) and implemented by Zakharko (31) to create a geodesic hexagonal grid used for assigning languages to geographic units via their Glottolog point coordinates. We generated grids at two resolutions: one grid measured a median cell diameter of 500 km, producing 1,139 cells across the world’s land mass (including islands), and an alternative grid measured a median cell diameter of 300 km (2,833 cells).

The TLI and GBI datasets each include a majority of binary features and a minority of categorical, multistate features. These were modeled differently. For each language *i* assigned to one geohistorical area (area), one grid cell (grid.id) and one language family (family), the probability of each binary feature (N = 269 in TLI, N = 193 in GBI) was modeled as follows:

$${\mathrm{state}}_{i}∼Bernoulli\left(\pi_{i}\right),$$

$$\pi_{i}=\frac{\exp\left(\eta_{i}\right)}{1+\exp\left(\eta_{i}\right)},$$

$$\begin{array}{c} \eta_{i}=\alpha +\alpha_{{\mathrm{AREA}}_{i}}+\alpha_{\mathrm{GRID}.{\mathrm{ID}}_{i}}+\alpha_{{\mathrm{FAMILY}}_{i}}+t2({\mathrm{lon}}_{\mathrm{GRID}.{\mathrm{ID}}_{i}},{\mathrm{lat}}_{\mathrm{GRID}.{\mathrm{ID}}_{i}})\;\;\alpha ∼N\left(0,2\right), \end{array}$$

$$\alpha_{{\mathrm{AREA}}_{i}}∼N\left(0,\sigma_{{\mathrm{AREA}}_{i}}\right),$$

$$\alpha_{\mathrm{GRID}.{\mathrm{ID}}_{i}}∼N\left(0,\sigma_{\mathrm{GRID}.{\mathrm{ID}}_{i}}\right),$$

$$\alpha_{{\mathrm{FAMILY}}_{i}}∼N\left(0,\sigma_{{\mathrm{FAMILY}}_{i}}\right),$$

$$\sigma_{{\mathrm{AREA}}_{i}},\sigma_{\mathrm{GRID}.{\mathrm{ID}}_{i},}\sigma_{{\mathrm{FAMILY}}_{i}},\sigma_{\mathrm{spline}}∼N\left(0,2\right).$$

${State}_{i}$ denotes the state of the feature in language *i*. α indicates the intercept, i.e., the global baseline for the feature. The terms $\alpha_{{AREA}_{i}}$, $\alpha_{GRID.{ID}_{i}}$, and $\alpha_{{FAMILY}_{i}}$ indicate that this intercept can vary by area, grid cell, and family (random intercepts). The term $t2({lon}_{GRID.{ID}_{i}},{lat}_{GRID.{ID}_{i}})$ indicates a tensor product spline smoothing over the geographic location of the centroid of the grid cell the language is located in (coordinates in projection EPSG:8859). The intercept, all individual varying effects and the SD over the tensor product spline were each assumed to have normal priors with mean 0 and SD 2.

The probability of states in categorical features with J > 2 states (N = 64 in TLI, N = 3 in GBI) in each language *i* were modeled as follows:

${state}_{i}∼Categorical\left(\pi_{i1},\pi_{i2},\cdots ,\pi_{\mathrm{iJ}}\right)$, where $\sum_{j=1}^{J}\pi_{\mathrm{ij}}=1$

$$\pi_{\mathrm{ij}}=\frac{\exp\left(\eta_{\mathrm{ij}}\right)}{\sum_{j=1}^{J}\exp\left(\eta_{\mathrm{ij}}\right)}=\mathrm{softmax}(\eta_{\mathrm{ij}}),$$

$$\begin{array}{c} \eta_{\mathrm{ij}}=\alpha +\alpha_{{\mathrm{AREA}}_{i,j}}+\alpha_{\mathrm{GRID}.{\mathrm{ID}}_{i,j}}+\alpha_{{\mathrm{FAMILY}}_{i,j}}+t2_{j}({\mathrm{lon}}_{\mathrm{GRID}.{\mathrm{ID}}_{i}},{\mathrm{lat}}_{\mathrm{GRID}.{\mathrm{ID}}_{i}}),\\ \;\alpha ∼\;N\left(0,2\right), \end{array}$$

$$\alpha_{{\mathrm{AREA}}_{i,j}}∼N\left(0,\sigma_{{\mathrm{AREA}}_{i}}\right),$$

$$\alpha_{\mathrm{GRID}.{\mathrm{ID}}_{i,j}}∼N\left(0,\sigma_{\mathrm{GRID}.{\mathrm{ID}}_{i}}\right),$$

$$\alpha_{{\mathrm{FAMILY}}_{i,j}}∼N\left(0,\sigma_{{\mathrm{FAMILY}}_{i}}\right),$$

$$\sigma_{{\mathrm{AREA}}_{i}},\sigma_{\mathrm{GRID}.{\mathrm{ID}}_{i},}\sigma_{{\mathrm{FAMILY}}_{i}},\sigma_{\mathrm{spline}}∼N\left(0,2\right).$$

All terms have the same function as in the Bernoulli model and we also assumed the same priors. The only difference is that the probability estimates for each of the J > 2 states are drawn from a Categorical distribution for each language *i*.

For each feature, all available languages were modeled in a GAMM implemented using the brms (81) interface to Stan (82, 83) in R (84).

We then extracted draws from the expected value of the posterior predictive distribution of each of these models, obtaining state probability estimates per feature and language. Since each language is attributed to one grid cell, we could then compute feature-wise estimates of the normalized Shannon entropy (H_n_) of the probabilities of all feature states per grid cell:

$$H_{n}=-\frac{\sum_{j=1}^{J}\pi_{j}\log(\pi_{j})}{\log(J)}.$$

We collected the posterior means and SD of H_n_ and of logit(H_n_) per feature and grid cell, separately for each dataset (TLI, GBI), grid resolution, and for the analyses using original and jittered coordinates. The models described in this section therefore act as “parametric bootstraps” to provide regularized estimates (with uncertainty) of grid-level entropy.

### Predictor Data Aggregation.

Predictor data (for an overview, see *SI Appendix*, Table S3) for our main models (see below) was aggregated at the level of the grid cells following an existing pipeline (21).

For the genetic predictor, we used the mean and SD of posterior cell-wise estimates of Wright’s *F*, computed from 5,737 unrelated individuals from 650 autochthonous populations with a sampling location that is sufficiently clear (median: 8 individuals per population) in the GeLaTo (“Genes and Languages Together”) database (1). *F* was calculated with PLINK v 1.9 (43) on the autosomal chromosomes for each individual in the dataset as the observed (*H*_*O*_) minus expected (*H*_*E*_) homozygous genotypes, divided by the number of nonmissing genotypes (i.e., the number of nonmissing SNPs) minus the expected number of homozygotes:

$$F=\frac{H_{O}-H_{E}}{N_{\mathrm{NM}}-H_{E}}.$$

The expected number of homozygotes is calculated from Hardy–Weinberg expectations, i.e. assuming *p*^2^ + 2*pq* + *q*^2^ = 1, where *p* corresponds to the frequency of the dominant allele, *q* corresponds to the frequency of the recessive allele, *p*^2^ corresponds to the expected frequency of homozygous dominant genotypes, *q*^2^ corresponds to the expected frequency of homozygous recessive genotypes and *2pq* denotes the expected frequency of heterozygous individuals.

Then, for each individual *i* assigned to one geohistorical area (area), one grid cell (grid.id), and one population (population), *F* was modeled as follows:

$$F_{i}∼N\left(\mu_{i,}\sigma_{i}\right),$$

$$\begin{array}{c} \mu_{i}=\alpha +\alpha_{{\mathrm{AREA}}_{i}}+\alpha_{\mathrm{GRID}.{\mathrm{ID}}_{i}}+\alpha_{{\mathrm{POPULATION}}_{i}}+t2({\mathrm{lon}}_{\mathrm{GRID}.{\mathrm{ID}}_{i}},{\mathrm{lat}}_{\mathrm{GRID}.{\mathrm{ID}}_{i}}),\\ \;\alpha ∼N\left(0,2\right), \end{array}$$

$$\alpha_{{\mathrm{AREA}}_{i}}∼N\left(0,\sigma_{{\mathrm{AREA}}_{i}}\right),$$

$$\alpha_{\mathrm{GRID}.{\mathrm{ID}}_{i}}∼N\left(0,\sigma_{\mathrm{GRID}.{\mathrm{ID}}_{i}}\right),$$

$$\alpha_{{\mathrm{FAMILY}}_{i}}∼N\left(0,\sigma_{{\mathrm{FAMILY}}_{i}}\right),$$

$$\sigma_{{\mathrm{AREA}}_{i}},\sigma_{\mathrm{GRID}.{\mathrm{ID}}_{i},}\sigma_{{\mathrm{FAMILY}}_{i}},\sigma_{\mathrm{spline}},\sigma_{i}∼N\left(0,2\right).$$

$F_{i}$ denotes Wright’s coefficient *F* in individual *i*. α indicates the intercept, i.e. the global baseline for *F*. The terms $\alpha_{{AREA}_{i}}$, $\alpha_{GRID.{ID}_{i}}$, and $\alpha_{{POPULATION}_{i}}$ indicate that this intercept can vary by area, grid cell, and population (random intercepts). The term $t2({lon}_{GRID.{ID}_{i}},{lat}_{GRID.{ID}_{i}})$ indicates a tensor product spline smoothing over the geographic location of the centroid of the grid cell the individual was sampled in (coordinates in projection EPSG:8859). The intercept, all individual varying effects, and the SD over the tensor product spline were each assumed to have normal priors with mean 0 and SD 2.

For the (log) language richness predictor, we used the cell-wise logarithm of the count of languages and dialects according to Glottolog, v. 5.0 (37).

For the (log) taxonomic diversity predictor, we used the cell-wise logarithm of the taxonomic diversity of the locally attested languages and dialects according to the Glottolog taxonomy (37). The taxonomic diversity index used is based on the index adopted in the R package “densify” (85). It adjusts for uneven taxonomic depths and node occurrences, producing a single diversity score based on richness of taxonomic levels: Cells score higher when they contain more families or more branches at similar taxonomic levels (yielding a richer representation of possible branches within attested families). Cells score lower when they contain fewer families and/or when languages are concentrated in fewer branches within a family.

As environmental predictors, we used the centered and scaled first and second principal components of a probabilistic principal component analysis (PPCA) over eleven environmental variables from various sources (*SI Appendix*, Table S3) (21, 86, 87), performed in R using the pca() function of the pcaMethods package (88). Variables were extracted for the year 2000 CE, or, for the variables from WorldClim2 (86), for the averages for the years 1970–2000. The first PC of the environmental predictors had an R^2^ of 34.8 at the main spatial resolution and an R^2^ value of 35.2 at the resolution of the sensitivity analysis. Its main loadings were the number of months with mean temperature >15 °C (27.1% in the main analysis, 27.3% in the sensitivity analysis), mean annual temperature (23.7% in the main analysis, 23.3% in the sensitivity analysis), and temperature of the warmest quarter (22.7% at both resolutions). The second PC had an R^2^ of 18.6 in the main analysis and an R^2^ of 17.1 in the sensitivity analysis. Its main loadings were altitude (26.7% and 27.7% for the main and sensitivity resolutions, respectively), altitude variation (20.1% and 19.3%, respectively) and seasonal variance of precipitation (15.6% and 16.8%, respectively).

For the (log) population density predictor, we used the cell-wise logarithm of the median population density in the year 2000 CE extracted from HYDE, v.3.3 (87).

Finally, varying intercepts and slopes (random effects) by area were defined according to language assignments (36) to one of the ten continent-sized areas (Africa, W and SW Eurasia, N-C Asia, S/SE Asia, New Guinea and Oceania, Australia, W N America, E N America, C America and S America) delineated in the AUTOTYP database (33). We did not use areas with a finer resolution because these would lead to extreme sparsity and fail to adjust for the out-of-Africa gradient in the *F* statistic.

### Modeling Structural Diversity.

As the response variable of our main models, again implemented in the brms (81) interface to Stan (82, 83), we used the logit-transformed posterior mean entropies per grid cell $\left[\mathrm{logit}({H_{n}}_{i})\right]$ and the corresponding SD of these estimates $\left[\mathrm{sd}(\mathrm{logit}({H_{n}}_{i})\right]$ from the features of TLI (N = 333) and GBI (N = 196) separately, for each of the original and the jittered language coordinates. In our models, the total variance of each observation was composed of the observed SD and an additional residual SDσ, which was estimated by the model, following a normal prior with mean 0 and SD 2.

$$\mathrm{logit}({H_{n}}_{i})∼N\left(\eta_{i},\sigma_{i}\right),$$

$$\sigma_{i}=\sqrt{\mathrm{sd}{\left(\mathrm{logit}({H_{n}}_{i})\right)}^{2}+\sigma^{2}},$$

σ=N(0,2).

Our null model m1, including only (log) language richness and (log) taxonomic diversity as main predictors, was defined as follows:

$$\begin{array}{c} \eta_{i}=\alpha +\alpha_{{\mathrm{FEATURE}}_{i}}+\alpha_{{\mathrm{AREA}}_{i}}+\alpha_{\mathrm{GRID}.{\mathrm{ID}}_{i}}+\left(\beta_{R}+\beta_{R,{\mathrm{FEATURE}}_{i}}+\beta_{R,{\mathrm{AREA}}_{i}}\right)\;\times R_{i}\\ \;+\left(\beta_{T}+\beta_{T,{\mathrm{FEATURE}}_{i}}+\beta_{T,{\mathrm{AREA}}_{i}}\right)\times T_{i}\\ +t2\left({\mathrm{lon}}_{\mathrm{GRID}.{\mathrm{ID}}_{i}},{\mathrm{lat}}_{\mathrm{GRID}.{\mathrm{ID}}_{i}}\right),\\ \left[\begin{array}{c} \alpha_{\mathrm{FEATURE}}\\ \beta_{R,\mathrm{FEATURE}}\\ \beta_{T,\mathrm{FEATURE}} \end{array}\right]∼mvN\left(\left[\begin{array}{c} 0\\ 0\\ 0 \end{array}\right],\Sigma_{\mathrm{FEATURE}}\right), \end{array}$$

$$\Sigma_{\mathrm{FEATURE}}=\left[\begin{array}{ccc} \sigma_{\alpha_{\mathrm{FEATURE}}}^{2} & \sigma_{\alpha_{\mathrm{FEATURE}}}\sigma_{\beta_{R,\mathrm{FEATURE}}}\rho & \sigma_{\alpha_{\mathrm{FEATURE}}}\sigma_{\beta_{T,\mathrm{FEATURE}}}\rho \\ \sigma_{\alpha_{\mathrm{FEATURE}}}\sigma_{\beta_{R,\mathrm{FEATURE}}}\rho & \sigma_{\beta_{R,\mathrm{FEATURE}}}^{2} & \sigma_{\beta_{R,\mathrm{FEATURE}}}\sigma_{\beta_{T,\mathrm{FEATURE}}}\rho \\ \sigma_{\alpha_{\mathrm{FEATURE}}}\sigma_{\beta_{T,\mathrm{FEATURE}}}\rho & \sigma_{\beta_{R,\mathrm{FEATURE}}}\sigma_{\beta_{T,\mathrm{FEATURE}}}\rho & \sigma_{\beta_{T,\mathrm{FEATURE}}}^{2} \end{array}\right],$$

$$\left[\begin{array}{c} \alpha_{\mathrm{AREA}}\\ \beta_{R,\mathrm{AREA}}\\ \beta_{T,\mathrm{AREA}} \end{array}\right]∼mvN\left(\left[\begin{array}{c} 0\\ 0\\ 0 \end{array}\right],\Sigma_{\mathrm{AREA}}\right),$$

$$\Sigma_{\mathrm{AREA}}=\left[\begin{array}{ccc} \sigma_{\alpha_{\mathrm{AREA}}}^{2} & \sigma_{\alpha_{\mathrm{AREA}}}\sigma_{\beta_{R,\mathrm{AREA}}}\rho & \sigma_{\alpha_{\mathrm{AREA}}}\sigma_{\beta_{T,\mathrm{AREA}}}\rho \\ \sigma_{\alpha_{\mathrm{AREA}}}\sigma_{\beta_{R,\mathrm{AREA}}}\rho & \sigma_{\beta_{R,\mathrm{AREA}}}^{2} & \sigma_{\beta_{R,\mathrm{AREA}}}\sigma_{\beta_{T,\mathrm{AREA}}}\rho \\ \sigma_{\alpha_{\mathrm{AREA}}}\sigma_{\beta_{T,\mathrm{AREA}}}\rho & \sigma_{\beta_{R,\mathrm{AREA}}}\sigma_{\beta_{T,\mathrm{AREA}}}\rho & \sigma_{\beta_{T,\mathrm{AREA}}}^{2} \end{array}\right].$$

α indicates the intercept, β_R_ indicates the fixed effect of the centered and scaled logarithm of language richness (R). β_T_ indicates the fixed effect of the centered and scaled logarithm of taxonomic diversity (T). The terms $\alpha_{{FEATURE}_{i}}$, $\alpha_{{AREA}_{i}}$, and $\alpha_{GRID.{ID}_{i}}$ indicate that the intercept varies by feature, by area of the grid cell, and by grid cell. $\beta_{R,{FEATURE}_{i}}$ and $\beta_{T,{FEATURE}_{i}}$ indicate that both the richness and the taxonomic diversity slopes vary by feature. $\beta_{R,{AREA}_{i}}$ and $\beta_{T,{AREA}_{i}}$ indicate that the richness and the taxonomic diversity slopes further vary by area. Finally, the term $t2\left({lon}_{GRID.{ID}_{i}},{lat}_{GRID.{ID}_{i}}\right)$ indicates a tensor product spline for smooths over the geographic location of the grid cell (coordinates in Equal Earth Asia-Pacific projection EPSG:8859).

We set the following priors on model parameters:

$$\alpha ,\beta_{R},\beta_{T}∼N\left(0,2\right),$$

$$\alpha_{{\mathrm{FEATURE}}_{i}},\beta_{R,{\mathrm{FEATURE}}_{i}},\beta_{T,{\mathrm{FEATURE}}_{i}}∼N\left(0,\sigma_{{\mathrm{FEATURE}}_{i}}\right),$$

$$\alpha_{{\mathrm{AREA}}_{i}},\beta_{R,{\mathrm{AREA}}_{i}},\beta_{T,{\mathrm{AREA}}_{i}}∼N\left(0,\sigma_{{\mathrm{AREA}}_{i}}\right),$$

$$\alpha_{\mathrm{GRID}.{\mathrm{ID}}_{i}}∼N\left(0,\sigma_{\mathrm{GRID}.{\mathrm{ID}}_{i}}\right),$$

$$\sigma_{{\mathrm{FEATURE}}_{i}},\sigma_{{\mathrm{AREA}}_{i},}\sigma_{\mathrm{GRID}.{\mathrm{ID}}_{i}},\sigma_{\mathrm{spline}}∼N\left(0,2\right),$$

$$R_{\mathrm{FEATURE}},R_{\mathrm{AREA}}∼LKJ\left(2\right).$$

The intercept and each of the fixed effects were assumed to follow a normal prior with mean 0 and SD 2. Each varying effect term also individually followed a normal prior with mean 0 and SD 2. Jointly, the varying effects by feature followed multivariate normal priors with mean 0 and variance–covariance $\Sigma_{FEATURE}$. The effects by area followed multivariate normal priors with mean 0 and variance–covariance $\Sigma_{AREA}$. The covariance matrices were decomposed into a prior SD vector and a correlation matrix **R**. Each **R** matrix was individually drawn from an *LKJ*(2) prior. The tensor smooth term SD followed a normal prior with mean 0 and SD 2.

Models m2-m8 followed the same structure of model m1. In addition to all terms in m1, their linear predictors additionally included different combinations of further linear main effects (P1, P2, D and *F*) and corresponding varying slopes by feature ($\beta_{P1,{FEATURE}_{i}},\beta_{P2,{FEATURE}_{i}},\beta_{D,{FEATURE}_{i}},\beta_{F,{FEATURE}_{i}}$) and area ($\beta_{P1,{AREA}_{i}},\beta_{P2,{AREA}_{i}},\beta_{D,{AREA}_{i}},\beta_{F,{AREA}_{i}}$) (*SI Appendix*, Table S4).

Prior configurations in models m2-m8 were equivalent to m1, with sigma, the intercept, each fixed effect term, each varying effect term and the tensor smooth term SD individually following a normal prior with mean 0 and SD 2. Effects by feature and area each followed multivariate normal priors with mean 0 and variance–covariance $\Sigma_{FEATURE}$ and $\Sigma_{AREA}$ respectively. These variance–covariance matrices followed the structure of $\Sigma_{FEATURE}$ and $\Sigma_{AREA}$ from m1 but were appropriately redimensioned to include SD vectors and correlation matrices **R** accounting for all varying effects included.

In addition to all terms in m1, m2 included all (scaled) terms relating to the environment (i.e., P1 and P2 and all related terms). m3 additionally included all terms relating to (log) population density (i.e., D and all related terms). m4 additionally included all terms relating to individual genetic diversity (*F*, including measurement error in terms of the SD from the GAMM that adjusts for geohistorical area, population, and spatial autocorrelation; and all related terms). m5 additionally included all terms relating to the environment and population density (i.e., P1, P2, and D, and all related terms). m6 additionally included all terms relating to the environment and genetics (i.e., P1, P2, and *F*, and all related terms). m7 additionally included all terms relating to population density and genetics (i.e., D and *F*, and all related terms). Finally, m8, the full model, additionally included all terms relating to the environment, population density, and genetics (i.e., P1, P2, D, *F,* and all related terms). *SI Appendix*, Table S4 provides an overview of models m1-m8 in R notation.

### Reporting Posterior Distributions.

To summarize and visualize posterior distributions obtained from our models, we resort to the highest posterior density interval (HPDI) encompassing 89% of the posterior probability. This threshold is ultimately arbitrary—just as any other chosen level would be. Grand averages and marginal effects were estimated using the posterior_epred function in brms (81) (full code available in the OSF-repository: https://osf.io/2qgje) (89).

## Supplementary Material

Appendix 01 (PDF)

## Data Availability

Raw data, models and scripts have been deposited in the following OSF-repository: https://osf.io/2qgje (89).
