# Supplementary material for: An inverse correlation between structural linguistic and human genetic diversity
Source: Proc Natl Acad Sci U S A. 2026 May 1;123(18):e2526762123. doi: 10.1073/pnas.2526762123 (PMC13142977; doi:10.1073/pnas.2526762123)
Supplement: Supplementary file 1 — Appendix 01 (PDF) [file pnas.2526762123.sapp.pdf]

PNAS

Supporting Information for
An inverse correlation between structural linguistic and human genetic diversity

Anna Graff*, Erik J. Ringen, Taras Zakharko, Mark Stoneking, Kentaro K. Shimizu, Balthasar Bickel*, Chiara Barbieri

* Email: anna.graff@uzh.ch, balthasar.bickel@uzh.ch.

This PDF file includes:

Figures S1 to S24
Tables S1 to S4
SI References

Figures

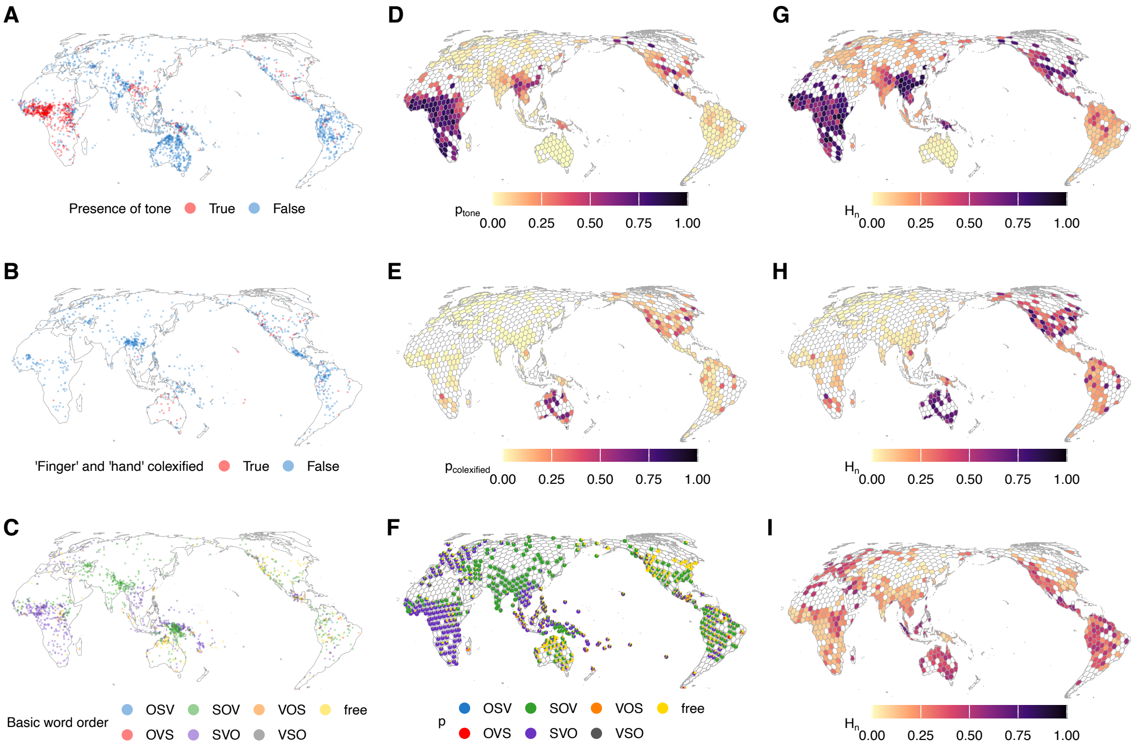

Fig. S1. Distributions of feature values (**A–C**), cell-wise probability estimates (**D–F**) and cell-wise normalized entropies (H_n) for the presence of tone (**A, D, G**), colexification for "finger" and "hand (**B, E, H**) and basic word order (**C, F, I**) after randomly jittering the original language coordinates from Glottolog within a radius of 250 km. Same plotting conventions as in Fig. 1.

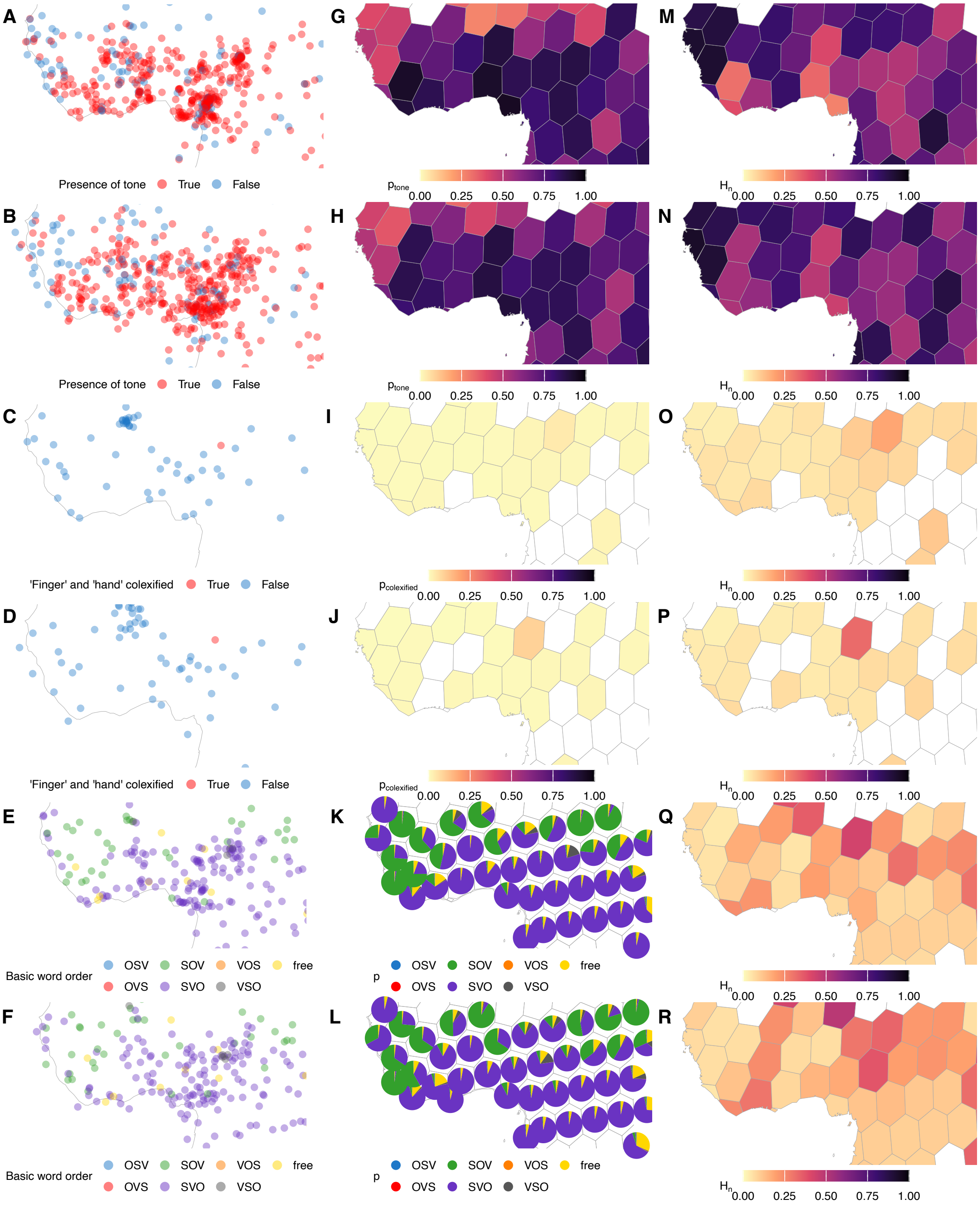

Fig. S2. Features and associated probability and entropy plots from Figs. 1 and S1, zoomed in on the Gulf of Guinea, showing both the Glottolog coordinate based distributions for tone (A, G, M), colexification of "finger" and "hand" (C, I, O) and basic word order (E, K, Q) and those using jittered coordinates (B, H, N), (D, J, P) and (F, L, R), respectively. Same plotting conventions as in Fig. 1.

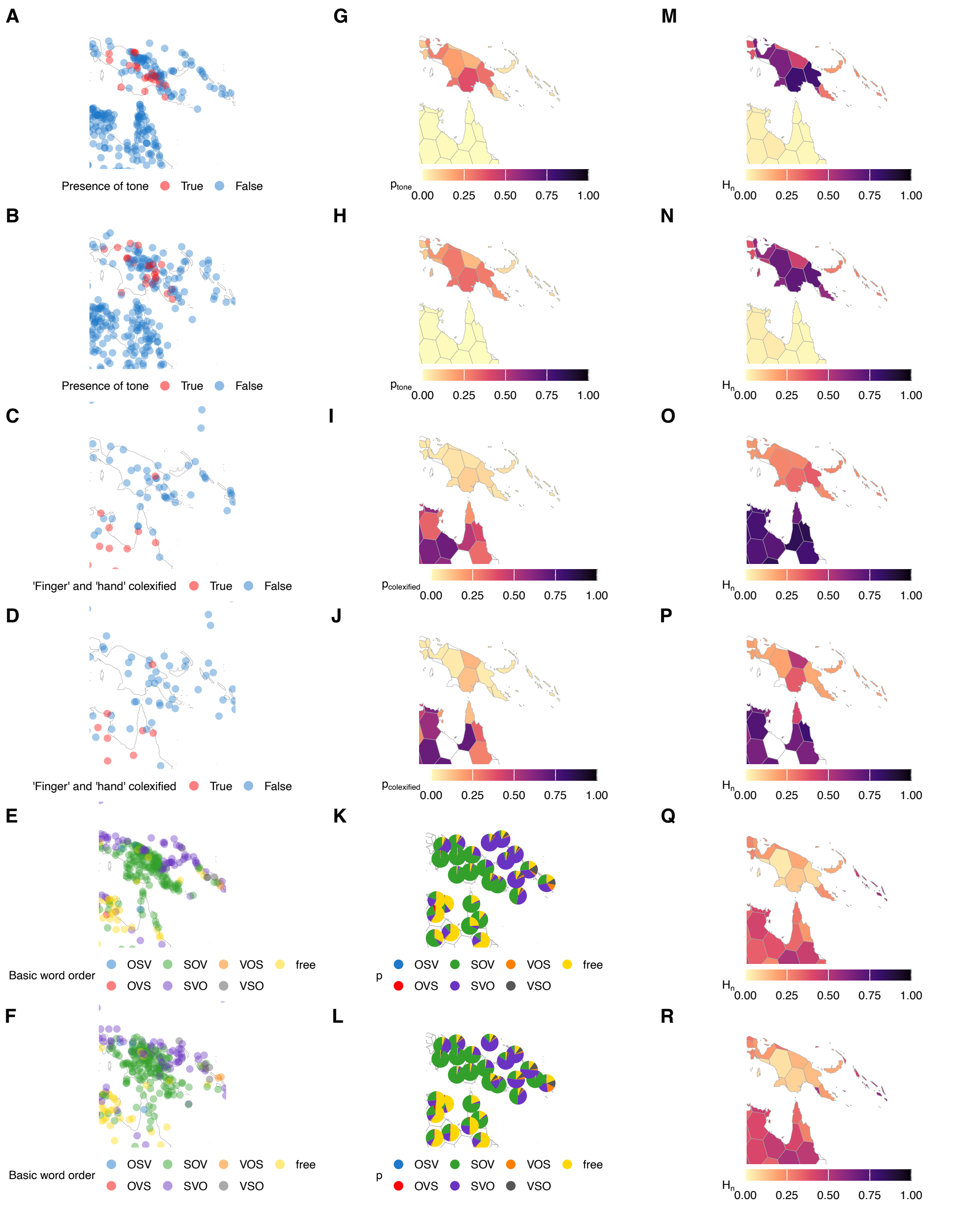

Fig. S3. Features and associated probability and entropy plots from Figs. 1 and S1, zoomed in on New Guinea and northern Australia, showing both the Glottolog coordinate based distributions for tone (A, G, M), colexification of "finger" and "hand" (C, I, O) and basic word order (E, K, Q) and those using jittered coordinates (B, H, N), (D, J, P) and (F, L, R), respectively. Same plotting conventions as in Fig. 1.

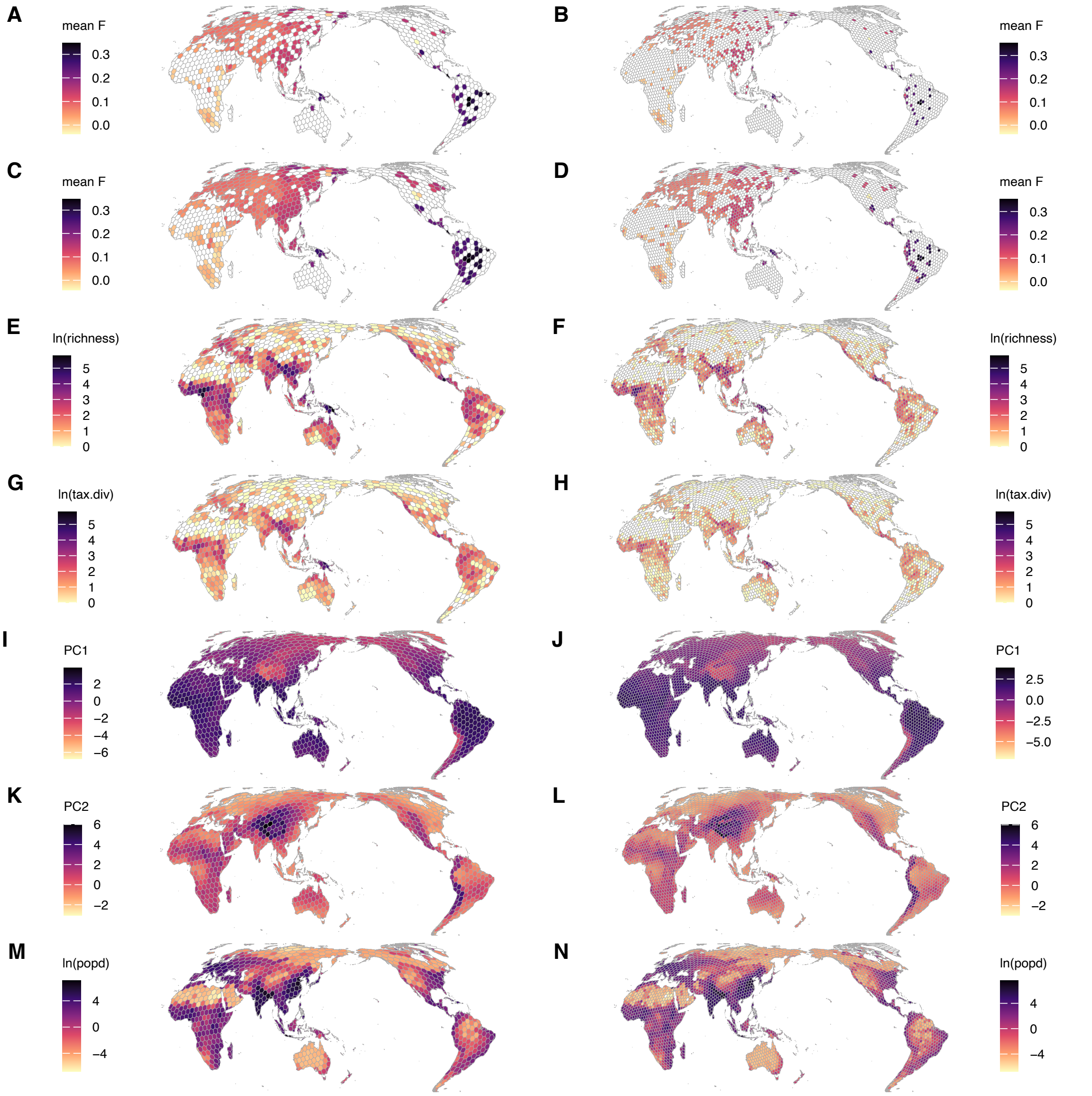

Fig. S4. Environmental and demographic predictors used for the models of local structural diversity, at grid cell diameters 500 km (**A,C,E,G,I,K,M**) and 300 km (**B,D,F,H,J,L,N**). (**A,B**) Mean posterior estimates of Wright's *F* coefficient, with increasing values indicating higher excess homozygosity and therefore lower levels of genetic diversity, derived from the original sample location coordinates. (**C,D**) Mean posterior estimates of Wright's *F* coefficient, derived after jittering individual sample coordinates by 250 km (**C**) and 150 km (**D**). (**E,F**) (Log) language richness, i.e. number of languages and dialects per cell. (**G,H**) (Log) taxonomic diversity of languages and dialects per cell, scoring how diverse and balanced the included languages' taxonomic relationships are. (**I,J**) First PC of environmental predictors. In the 500 km grid (R^2 = 34.8, **I**), the main loadings are the number of months with mean temperature > 15°C (27.1%), mean annual temperature (23.7%) and temperature of the warmest quarter (22.7%). In the 300 km grid (R^2 = 35.2, **J**), the main loadings are the number of months with mean temperature > 15°C (27.3%), mean annual temperature (23.3%) and temperature of the warmest quarter (22.7%). (**K,L**) Second PC of environmental predictors. In the 500 km grid (R^2 = 18.6, **K**), the main loadings are altitude (26.7%), altitude variation (20.1%) and seasonal variance of precipitation (15.6%). In the 300 km grid (R^2 = 17.1, **L**), the main loadings are altitude (27.7%), altitude variation (19.3%) and seasonal variance of precipitation (16.8%). (**M,N**) (Log) population density.

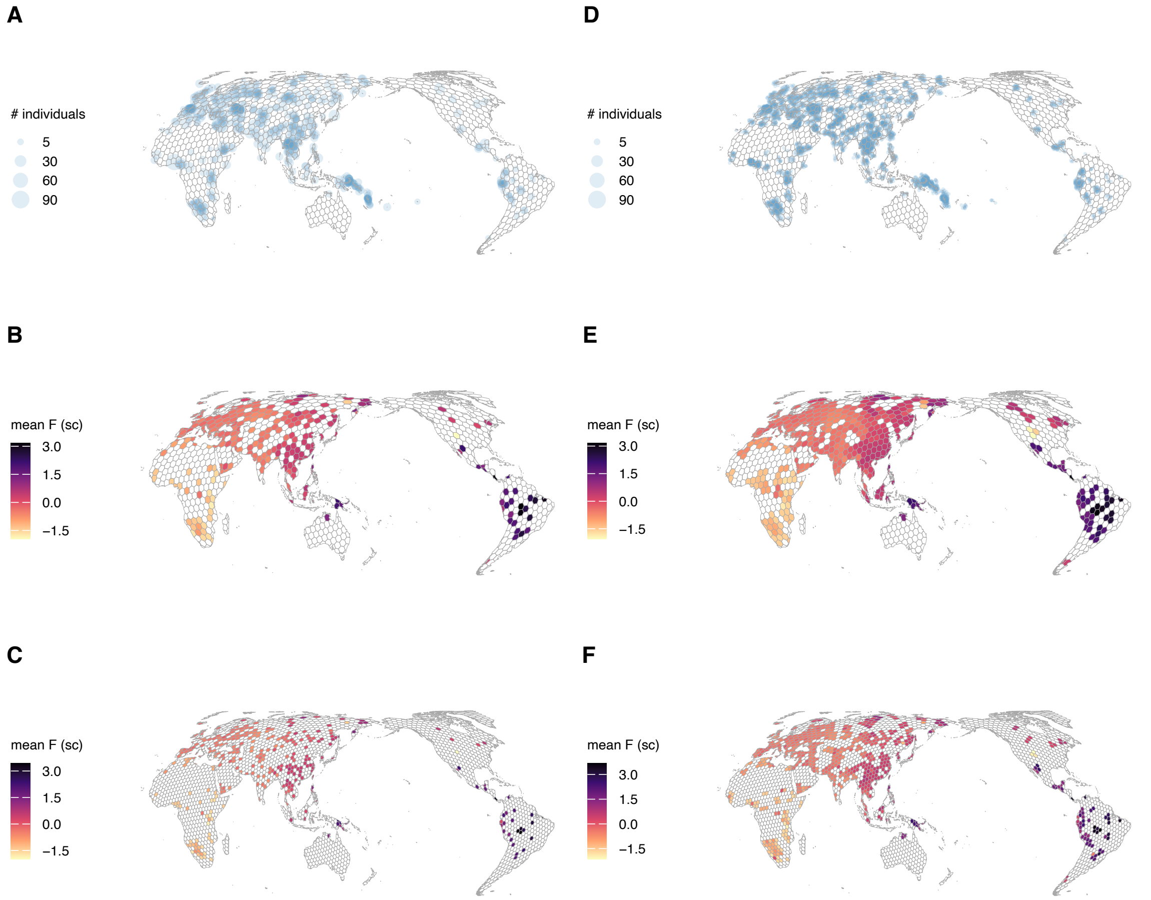

Fig. S5. Genetic samples and model-derived estimates for Wright's *F* per grid cell. (**A**) shows the distribution of individuals for which genetic data are available, according to the coordinates available in the original sources. (**B**) and (**C**) show the scaled (z-scored) model-derived estimates for *F* per grid cell, which entered the main models, considering the coarser and finer grid, respectively. (**D**) shows the distribution of these same individuals after randomly jittering the location for each individual within a radius of 250 km, and (**E**) presents the corresponding scaled model-derived *F*-estimates per grid cell. (**F**) illustrates the scaled model-derived estimates for *F* after randomly jittering locations by 150 km.

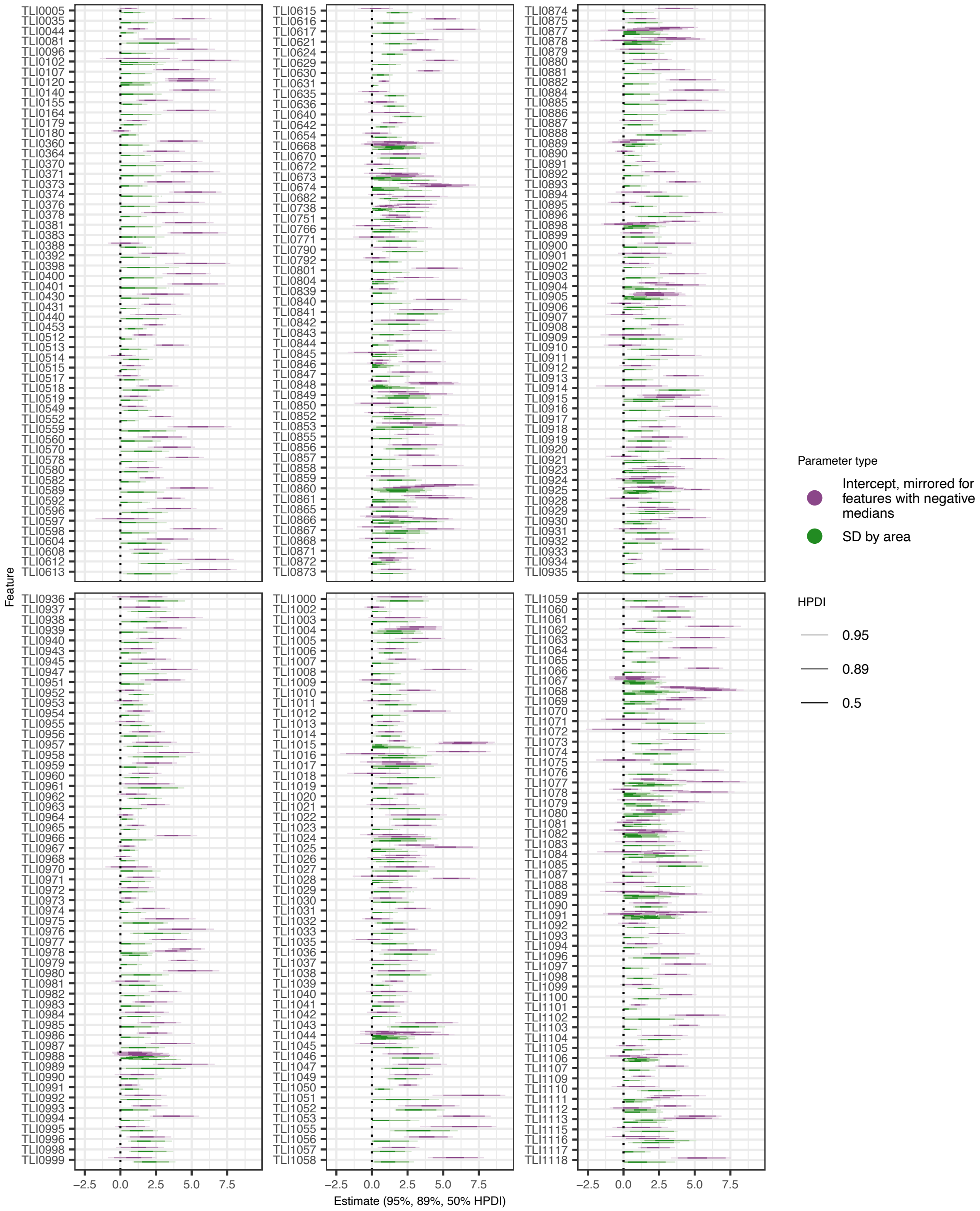

Fig. S6. Intervals denoting posterior draws for feature-wise intercepts (mirrored for features with negative medians) and standard deviations by area for the TLI dataset (500 km diameter) from the models using the original language coordinates from Glottolog. The figure exhibits feature-level variation regarding the empirical relative importance of the global intercept as compared to group-level variation by AUTOTYP-area.

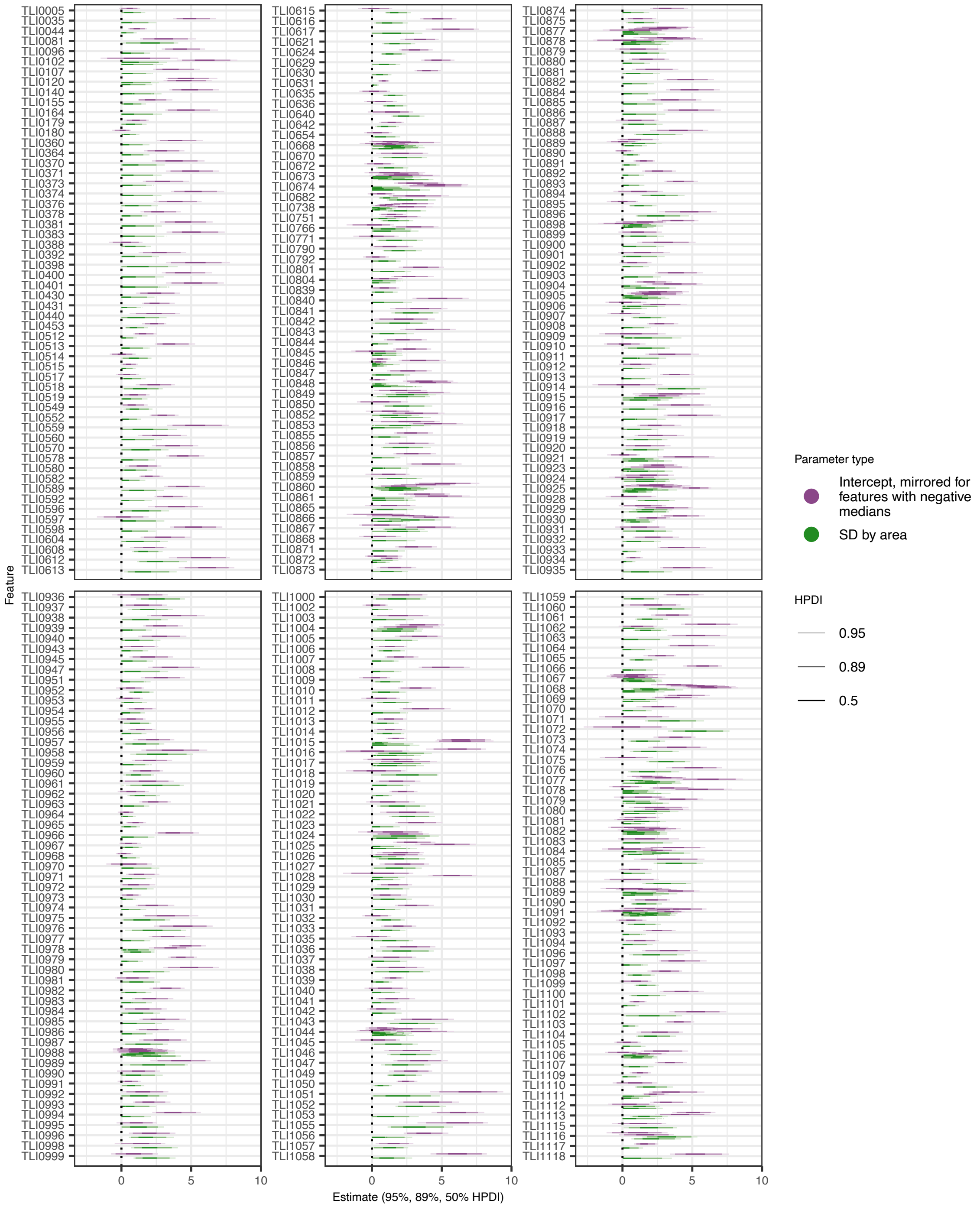

Fig. S7. Intervals denoting posterior draws for feature-wise intercepts (mirrored for features with negative medians) and standard deviations by area for the TLI dataset (500 km diameter) from the models using the language coordinates after random jittering within a radius of 250 km.

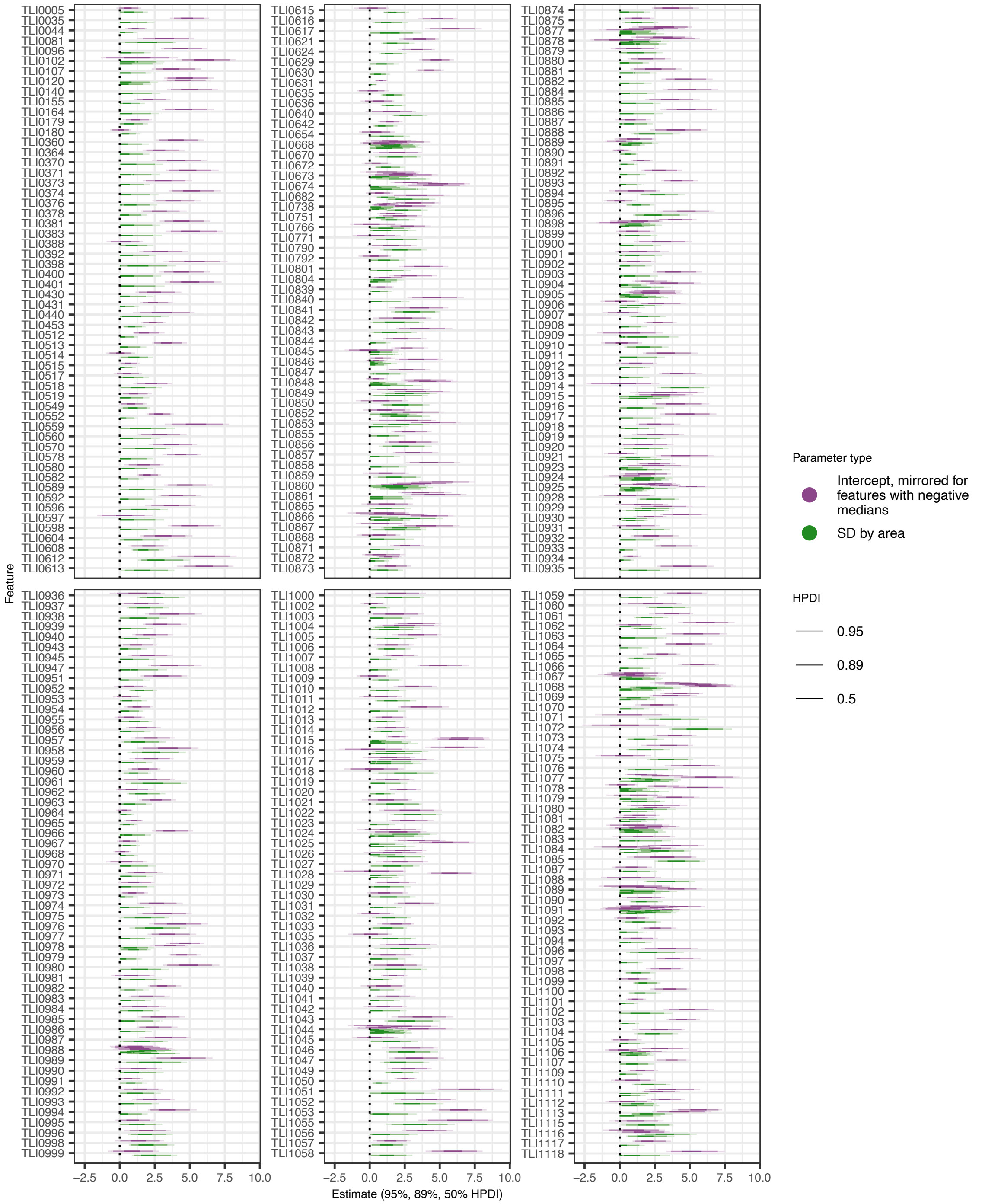

Fig. S8. Intervals denoting posterior draws for feature-wise intercepts (mirrored for features with negative medians) and standard deviations by area for the TLI dataset (300 km diameter) from the models using the original language coordinates from Glottolog.

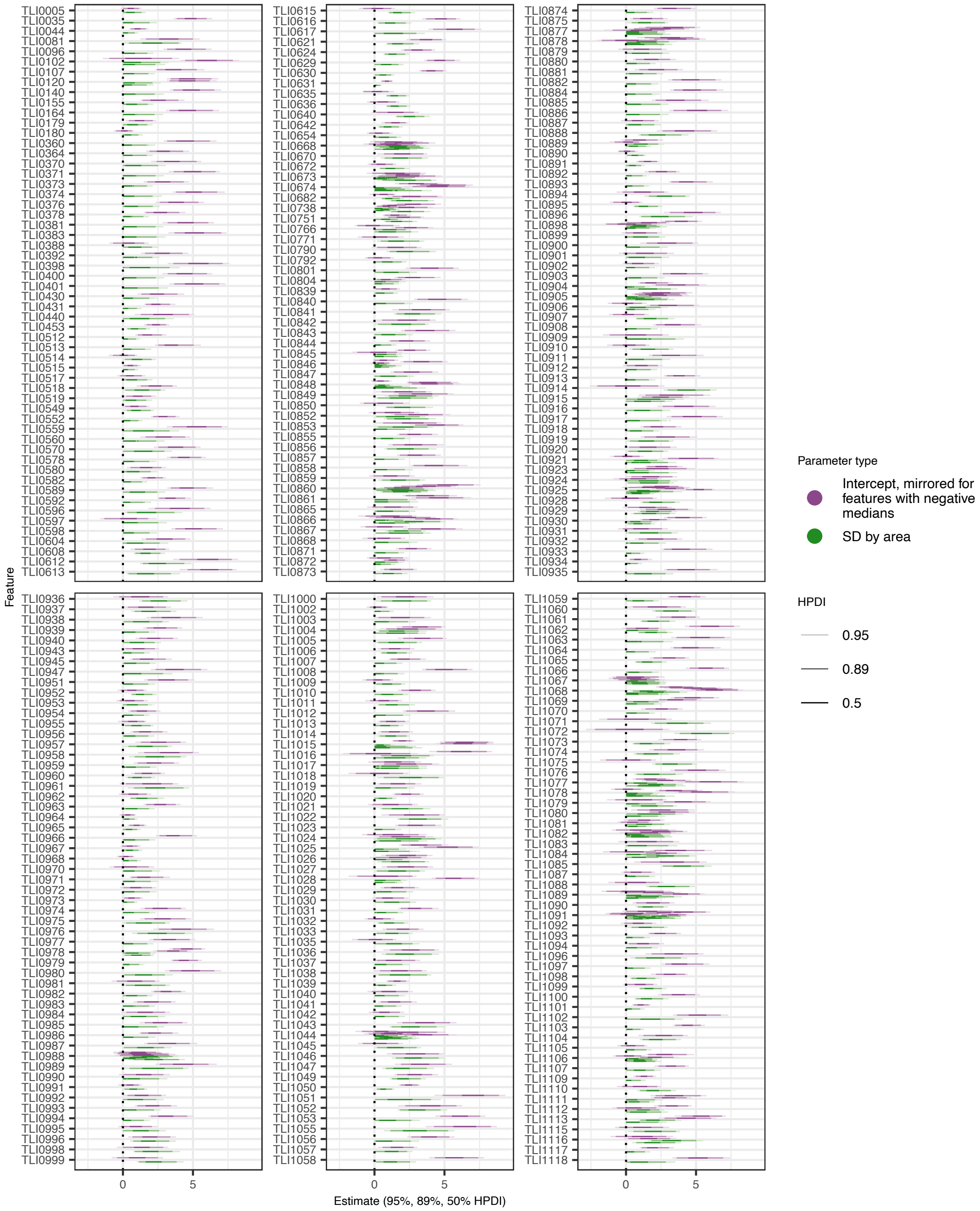

Fig. S9. Intervals denoting posterior draws for feature-wise intercepts (mirrored for features with negative medians) and standard deviations by area for the TLI dataset (300 km diameter) from the models using the language coordinates after random jittering within a radius of 150 km.

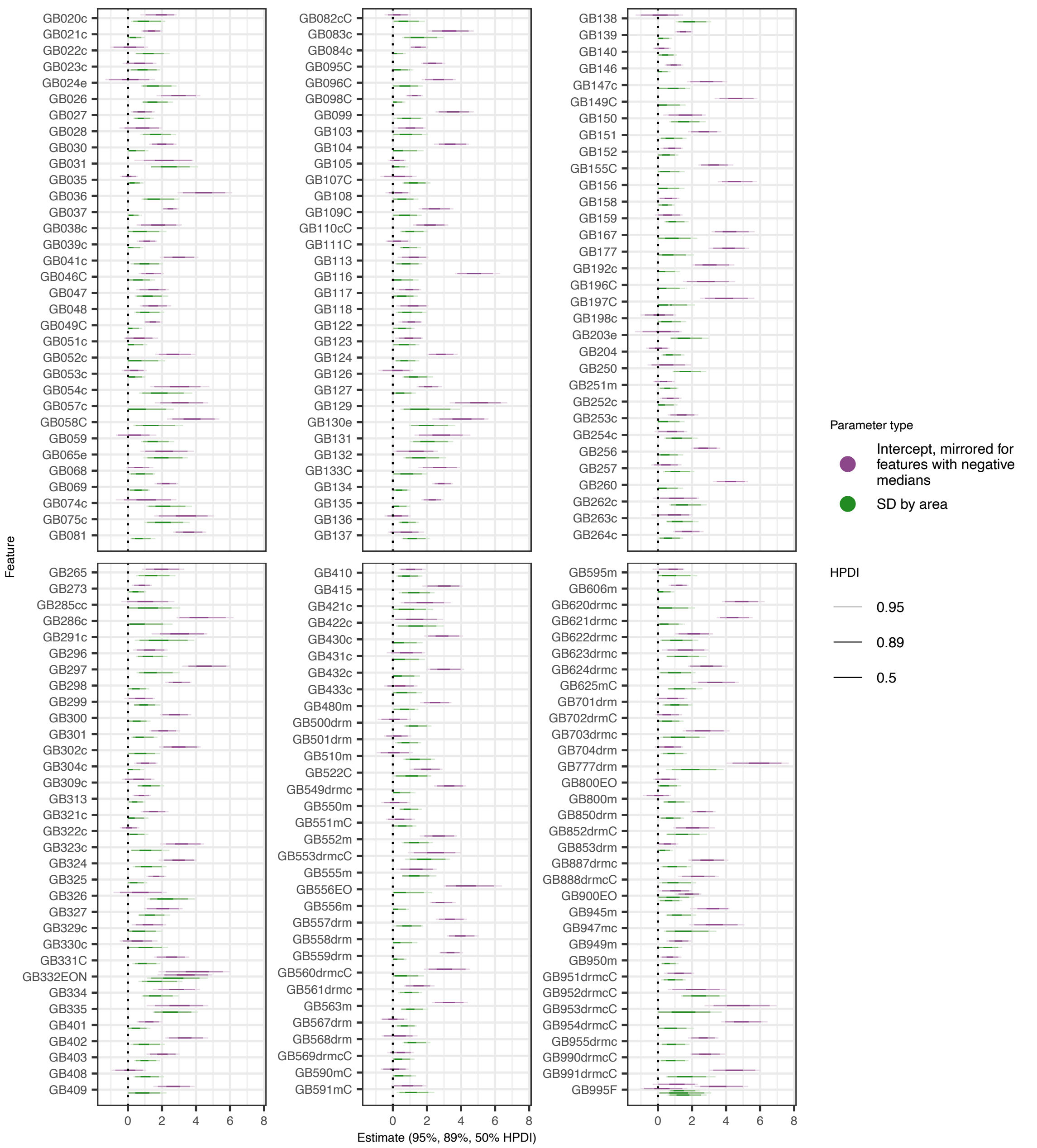

Fig. S10. Intervals denoting posterior draws for feature-wise intercepts (mirrored for features with negative medians) and standard deviations by area for the GBI dataset (500 km diameter) from the models using the original language coordinates from Glottolog.

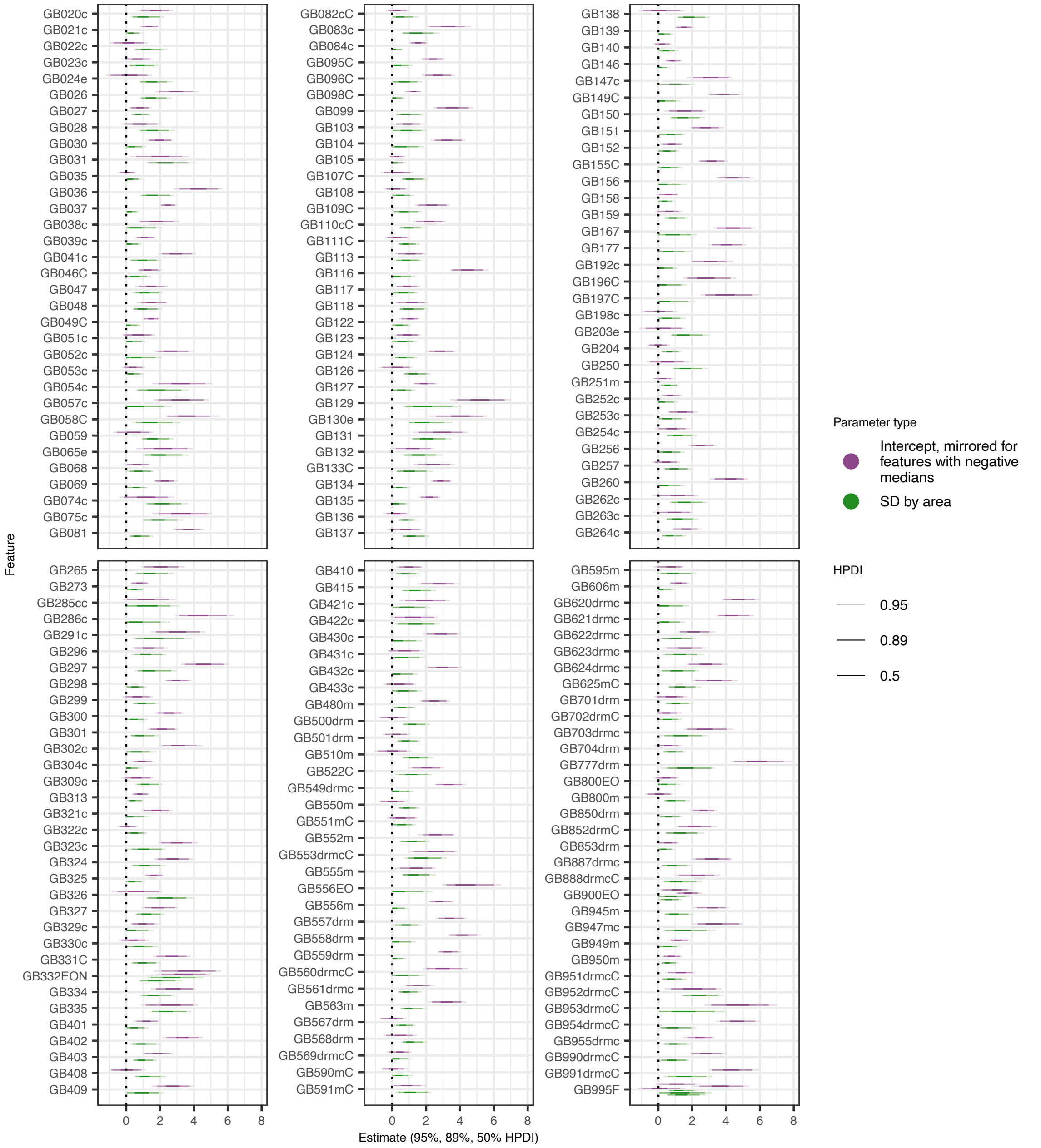

Fig. S11. Intervals denoting posterior draws for feature-wise intercepts (mirrored for features with negative medians) and standard deviations by area for the GBI dataset (500 km diameter) from the models using the language coordinates after random jittering within a radius of 250 km.

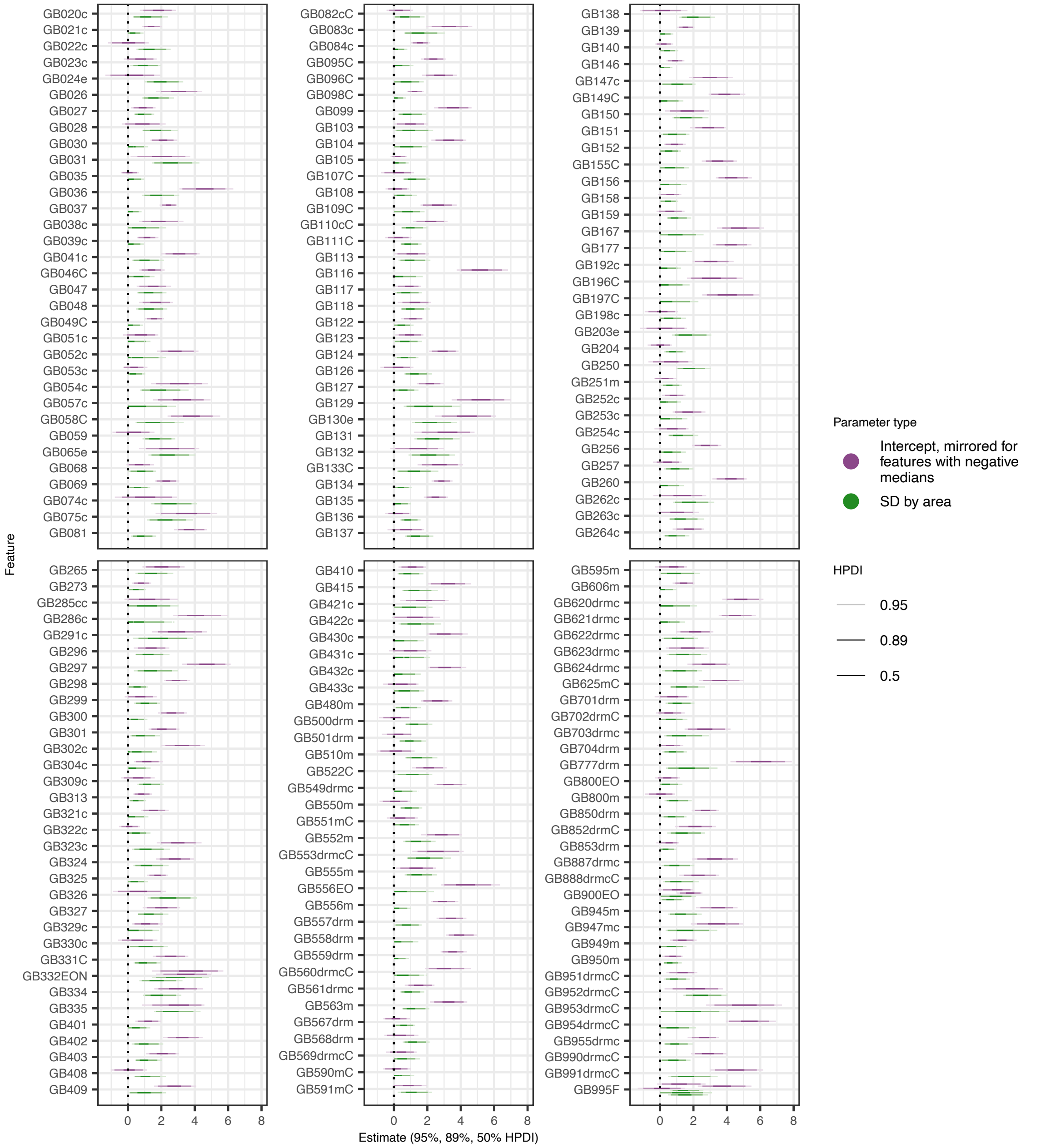

Fig. S12. Intervals denoting posterior draws for feature-wise intercepts (mirrored for features with negative medians) and standard deviations by area for the GBI dataset (300 km diameter) from the models using the original language coordinates from Glottolog.

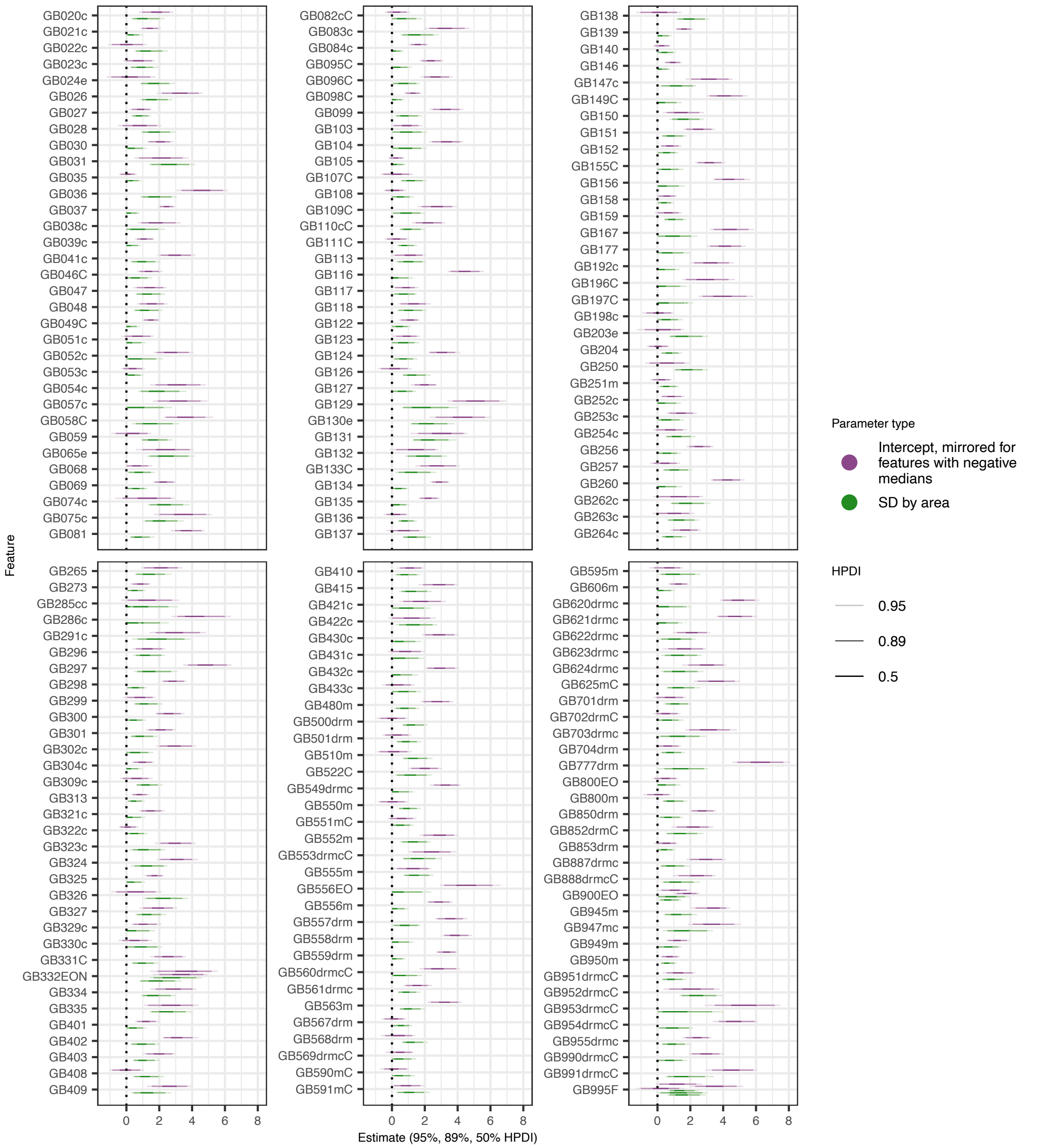

Fig. S13. Intervals denoting posterior draws for feature-wise intercepts (mirrored for features with negative medians) and standard deviations by area for the GBI dataset (300 km diameter) from the models using the language coordinates after random jittering within a radius of 150 km.

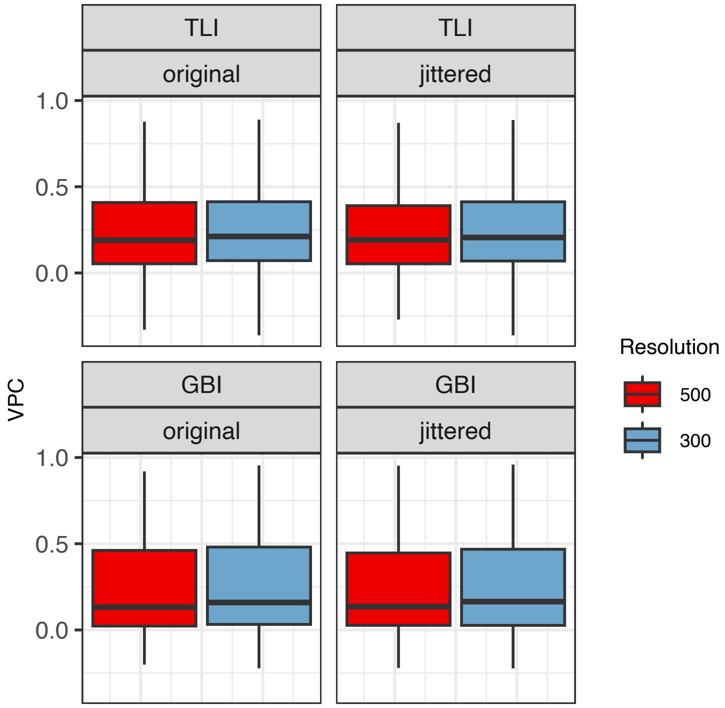

Fig. S14. Median variation partition coefficient (VPC) for all feature-wise models in both datasets and resolutions, using both the original and randomly jittered coordinates, following the logic implemented in variance_decomposition() in the *performance* R-package (1). The VPC is defined as 1 minus the ratio between the variance of posterior predictive distributions *not* conditioned by the varying intercepts of the random effects (i.e., global intercept only) and the variance of the distributions conditioned by the varying intercepts of the random effects). VPC-measures of 0 indicate that random effects do not account for more variation than just the baseline, while VPC-measures approaching 1 denote increasing importance of random effects.

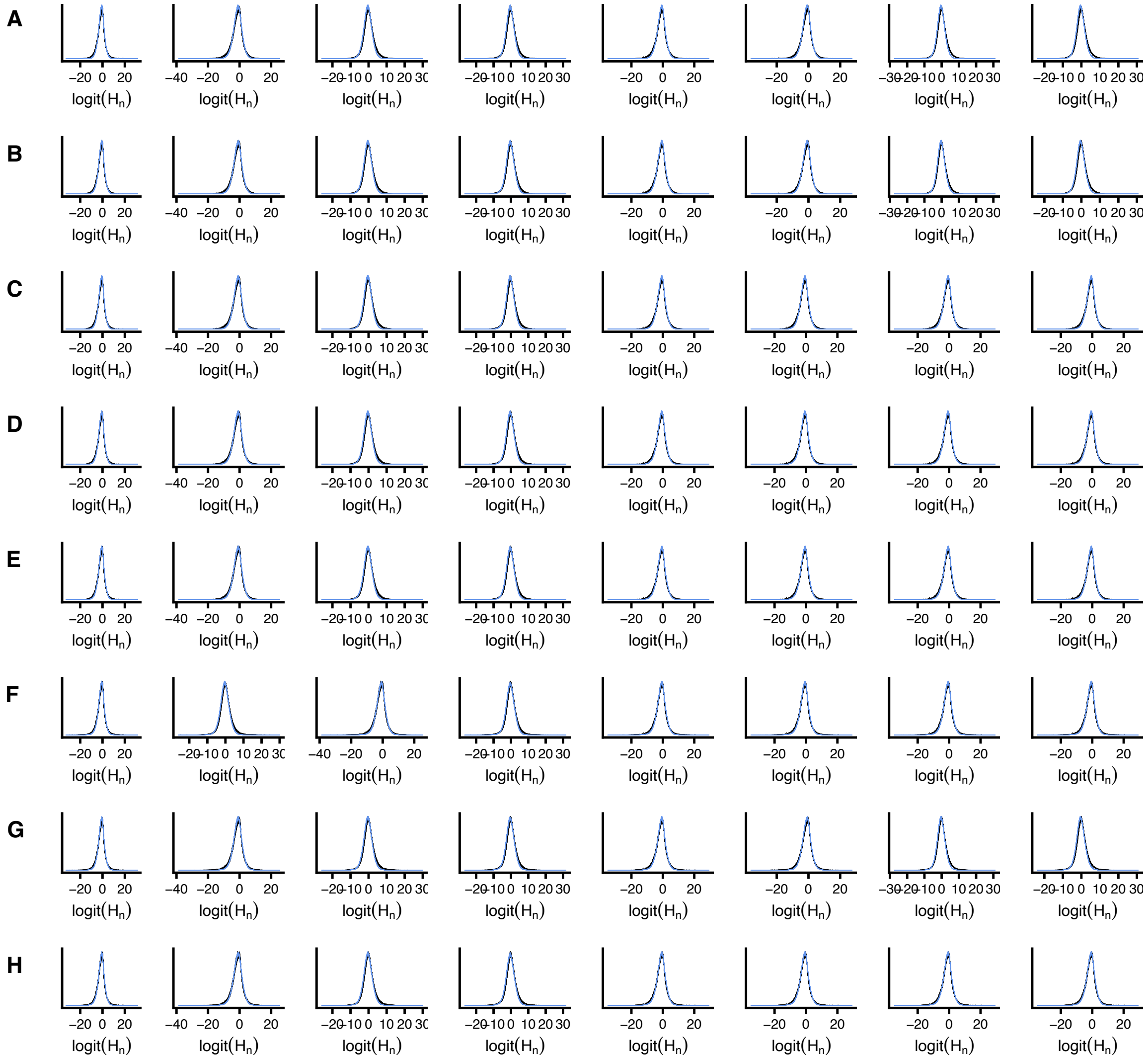

Fig. S15. Posterior predictive checks of all main analysis models, with 100 draws from feature-wise entropy predictions (feature models) in black and 100 predicted values including measurement error in blue. From left to right: TLI dataset, grid cell diameter 500 km, original coordinates; TLI dataset, grid cell diameter 300 km, original coordinates; GBI dataset, grid cell diameter 500 km, original coordinates; GBI dataset, grid cell diameter 300 km, original coordinates. TLI dataset, grid cell diameter 500 km, jittered coordinates; TLI dataset, grid cell diameter 300 km, jittered coordinates; GBI dataset, grid cell diameter 500 km, jittered coordinates; GBI dataset, grid cell diameter 300 km, jittered coordinates. (**A**) model m1. (**B**) model m2. (**C**) model m3. (**D**) model m4. (**E**) model m5. (**F**) model m6. (**G**) model m7. (**H**) model m8.

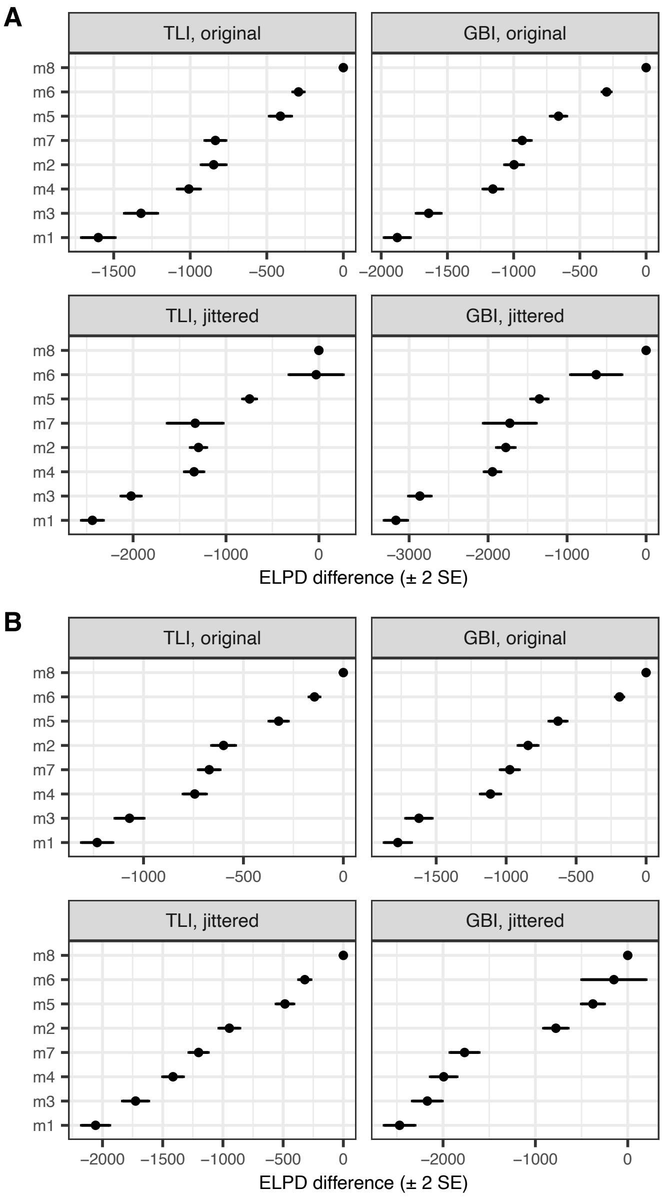

Fig. S16. Model comparison using ELPD differences between all main analysis models m1-m8 for the TLI-statistical and GBI-statistical linguistic datasets, both for the analyses using the original and jittered coordinates. (**A**) Grid cell diameter = 500 km. (**B**) Grid cell diameter = 300 km. The full model m8 including environmental as well as log population density and genetic predictors performs across conditions. Only in two analyses using jittered coordinates (TLI in the coarser grid and GBI in the finer grid) is the advantage of m8 over m6 (excluding log population density) not clearly supported, because the ELPD differences remain within 2 standard errors.

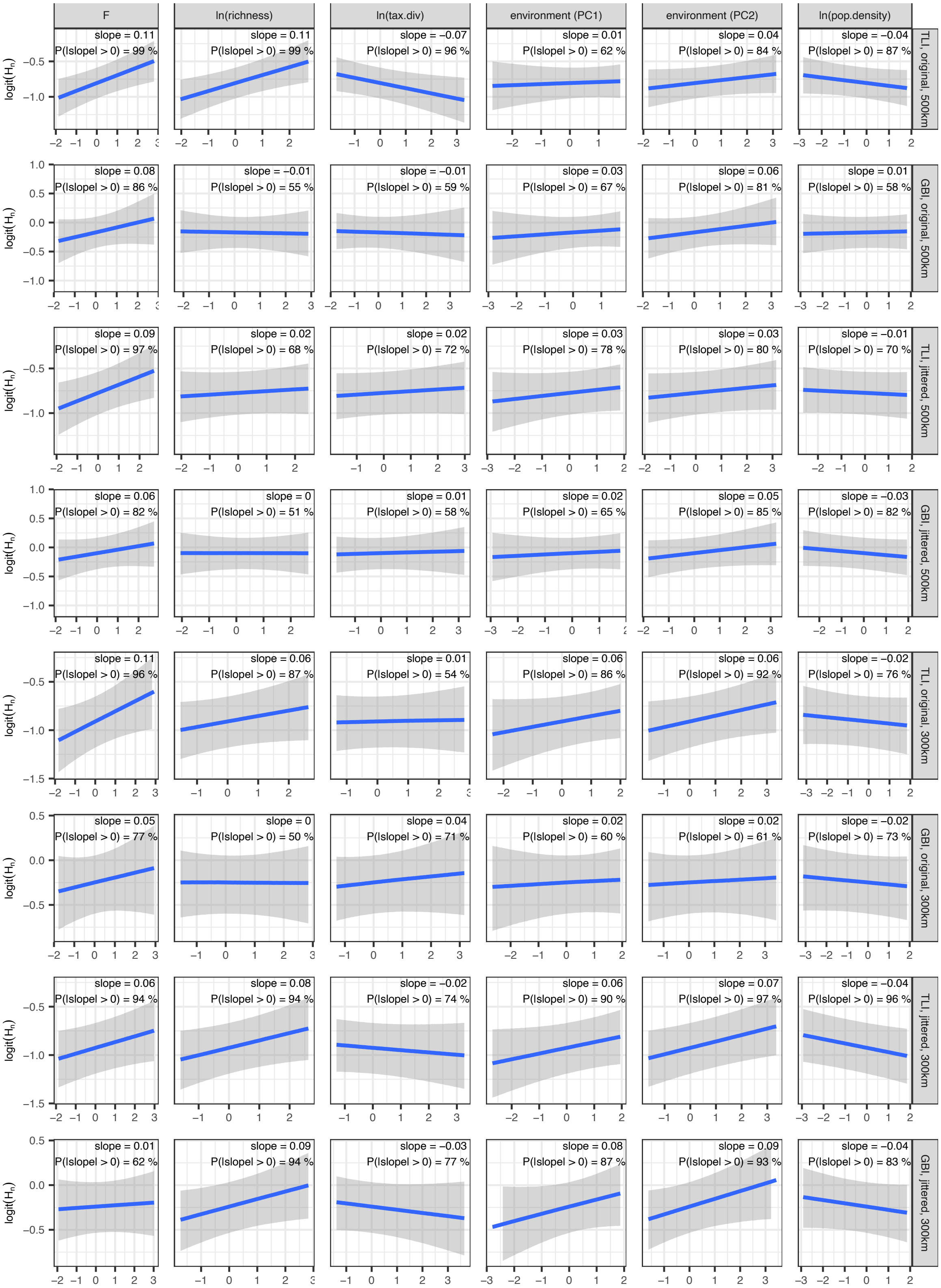

Fig. S17. Marginal effects at the mean of all main effects (F, log language richness, log taxonomic diversity, environmental PC1, environmental PC2 and log population density) in all analyses, i.e. at both geographic resolutions, with both the TLI and the GBI dataset, and using both the original and jittered coordinates. Intervals correspond to the 89% credible interval.

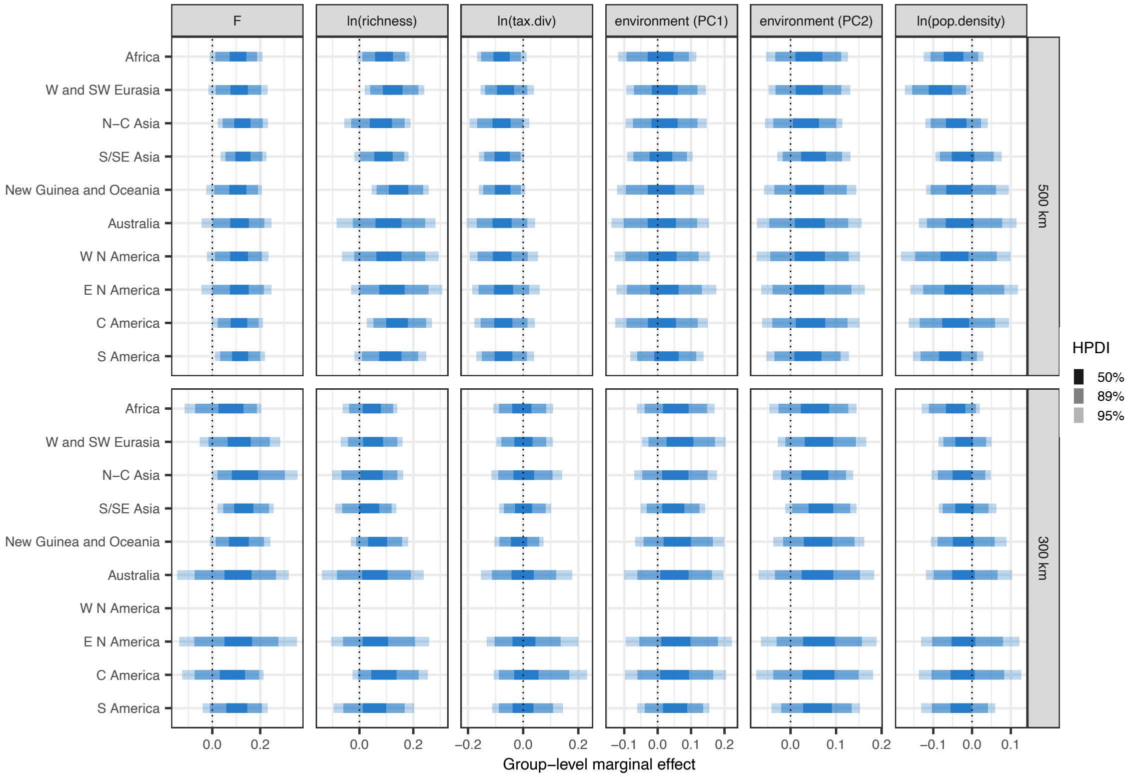

Fig. S18. Group-level marginal effects (slopes) of the six main predictors by geohistorical area as identified by AUTOTYP (2) in the TLI dataset at both resolutions (cell diameter 500 km and 300 km) in the analysis using the original coordinates.

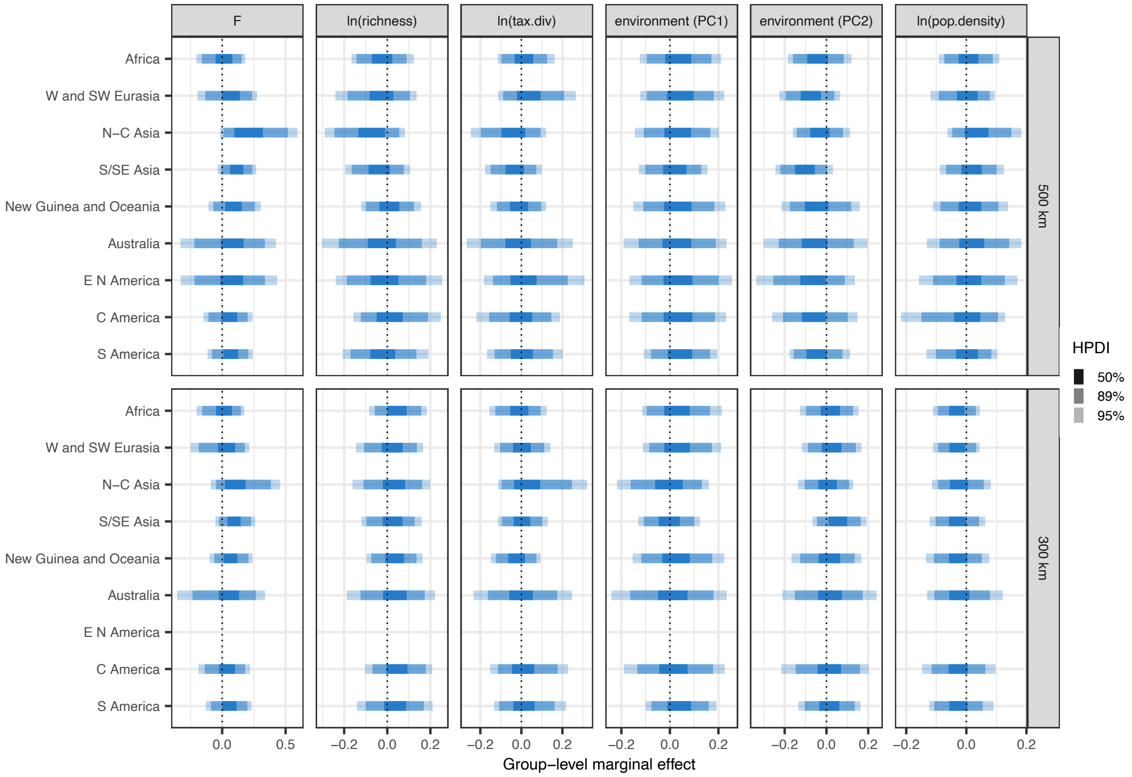

Fig. S19. Group-level marginal effects (slopes) of the six main predictors by geohistorical area as identified by AUTOTYP (2) in the sensitivity analysis using the GBI dataset at both resolutions (cell diameter 500 km and 300 km) in the analysis using the original coordinates.

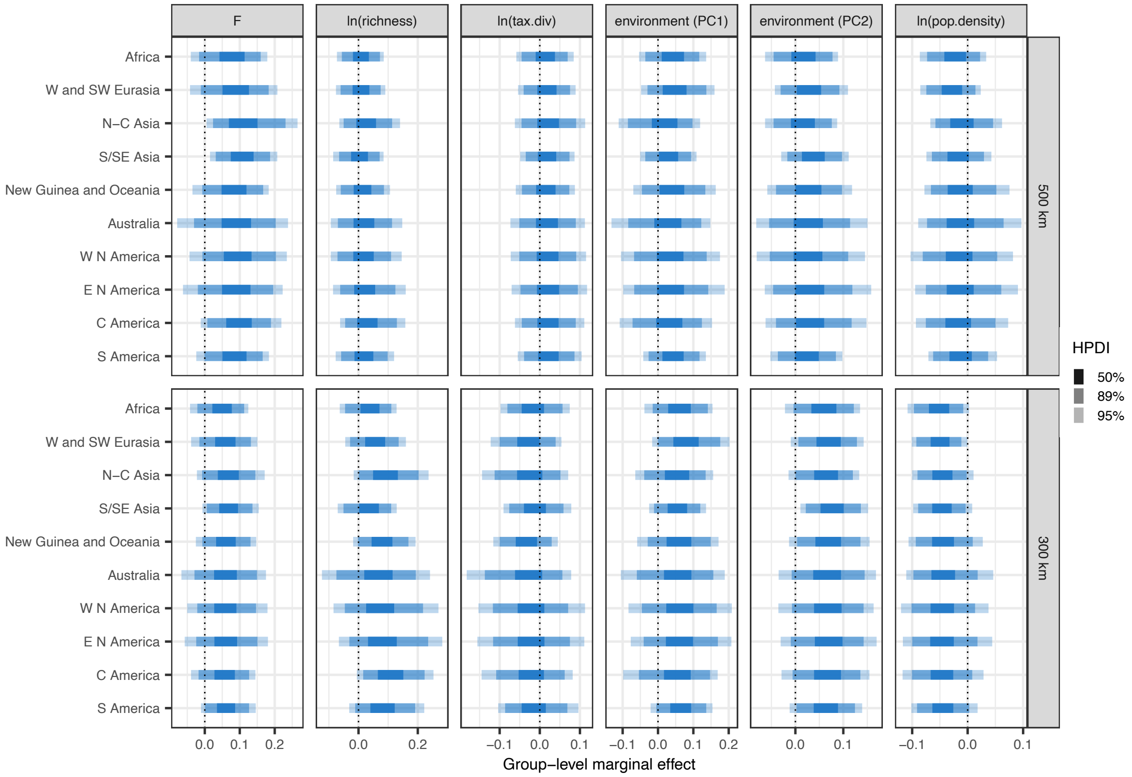

Fig. S20. Group-level marginal effects (slopes) of the six main predictors by geohistorical area as identified by AUTOTYP (2) in the TLI dataset at both resolutions (cell diameter 500 km and 300 km) in the analysis using jittered coordinates.

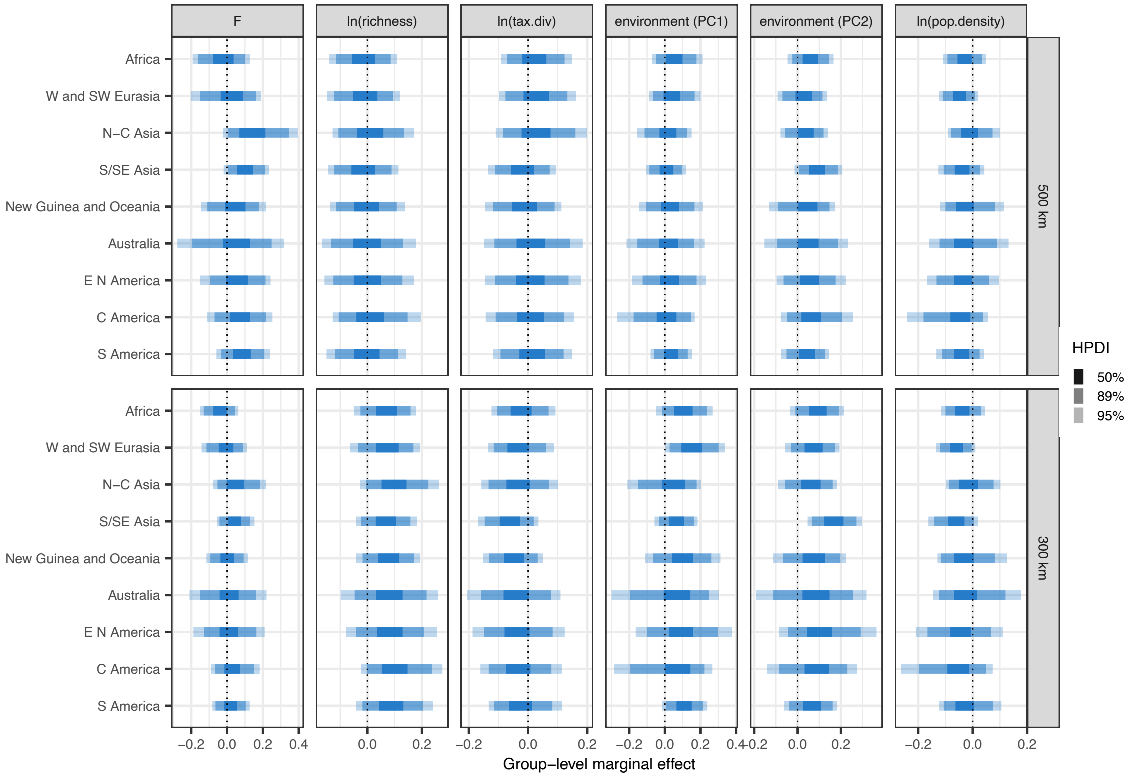

Fig. S21. Group-level marginal effects (slopes) of the six main predictors by geohistorical area as identified by AUTOTYP (2) in the GBI dataset at both resolutions (cell diameter 500 km and 300 km) in the analysis using jittered coordinates.

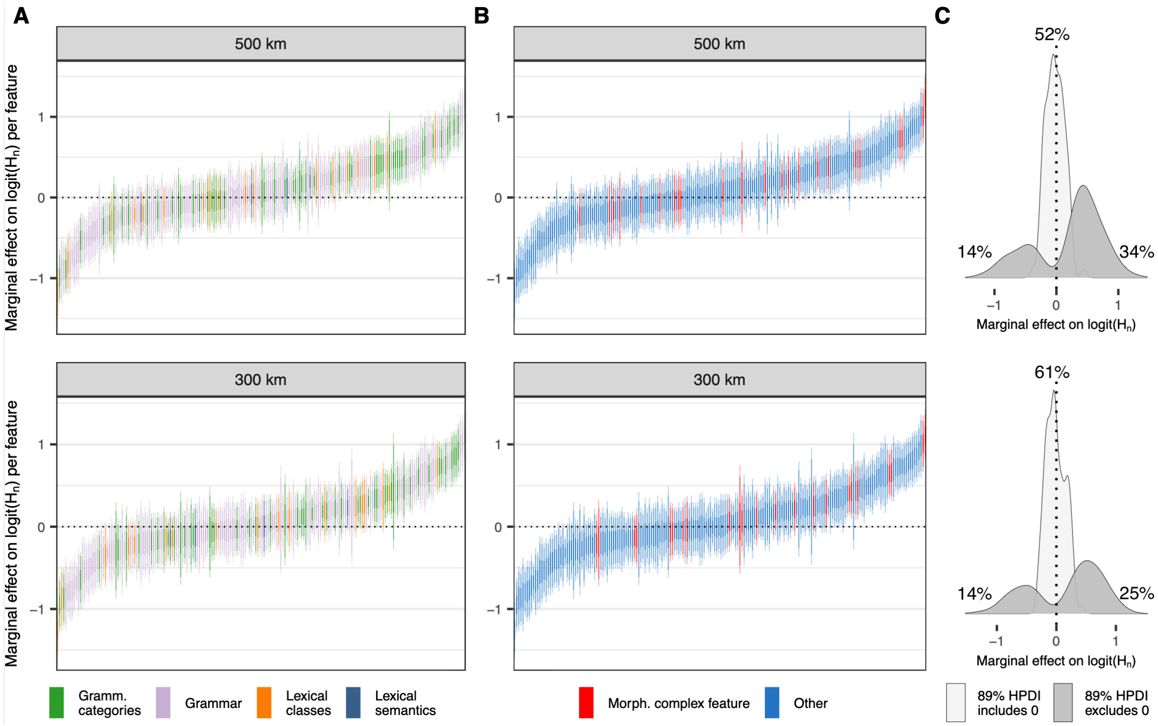

Fig. S22. Group-level marginal effects (slopes) of the genetic effect at each geographical resolution by feature (x-axis) in GBI, using the original coordinates. Same plotting conventions as in Fig. 4. The list of features can be found in Table S2 in the OSF repository (https://osf.io/2qgje).

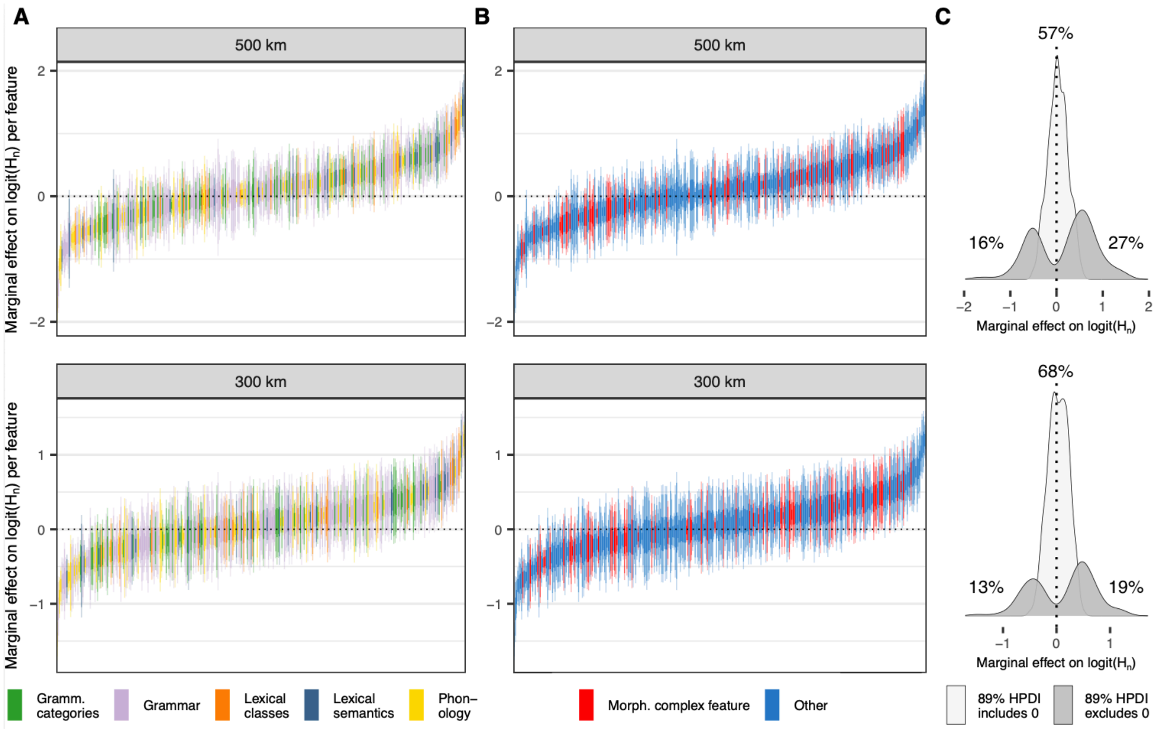

Fig. S23. Group-level marginal effects (slopes) of the genetic effect at each geographical resolution by feature (x-axis) in TLI, using the jittered coordinates. Same plotting conventions as in Fig. 4. The list of features can be found in Table S2 in the OSF repository (https://osf.io/2qgje).

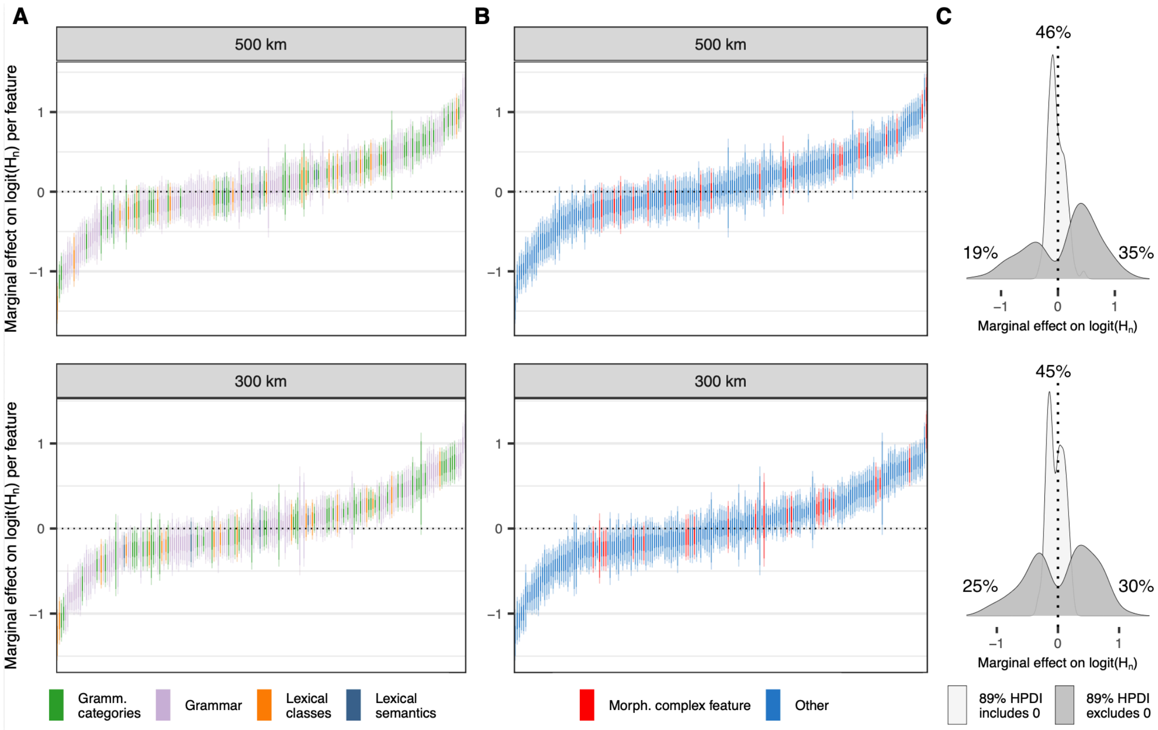

Fig. S24. Group-level marginal effects (slopes) of the genetic effect at each geographical resolution by feature (x-axis) in GBI, using the jittered coordinates. Same plotting conventions as in Fig. 4. The list of features can be found in Table S2 in the OSF repository (https://osf.io/2qgje).

Tables

Table S1.

Posterior probabilities for the effect of F in each area (random slopes) at each geographical resolution and for each dataset, both for the analyses using the original and jittered coordinates. Note that this table is also available as a .csv file on the OSF repository (https://osf.io/2qgje).

Dataset	Diameter	Coordinates	Autotyp area	Estimate	89% HPDI (lower)	89% HPDI (upper)	Posterior probability
TLI	300 km	original	Africa	0.058	-0.071	0.187	0.777
TLI	300 km	original	Australia	0.098	-0.074	0.266	0.858
TLI	300 km	original	C America	0.061	-0.073	0.196	0.782
TLI	300 km	original	E N America	0.104	-0.068	0.282	0.866
TLI	300 km	original	N-C Asia	0.161	0.022	0.302	0.981
TLI	300 km	original	New Guinea and Oceania	0.114	0.014	0.215	0.964
TLI	300 km	original	S America	0.098	-0.01	0.207	0.923
TLI	300 km	original	S/SE Asia	0.137	0.041	0.232	0.991
TLI	300 km	original	W and SW Eurasia	0.114	-0.014	0.242	0.929
TLI	500 km	original	Africa	0.101	0.013	0.188	0.957
TLI	500 km	original	Australia	0.105	-0.001	0.215	0.933
TLI	500 km	original	C America	0.108	0.023	0.194	0.972
TLI	500 km	original	E N America	0.107	0	0.214	0.939
TLI	500 km	original	N-C Asia	0.126	0.04	0.207	0.992
TLI	500 km	original	New Guinea and Oceania	0.098	0.008	0.191	0.943
TLI	500 km	original	S America	0.116	0.034	0.2	0.984
TLI	500 km	original	S/SE Asia	0.132	0.055	0.208	0.997
TLI	500 km	original	W and SW Eurasia	0.11	0.015	0.205	0.96
TLI	500 km	original	W N America	0.108	0.011	0.207	0.951
GBI	300 km	original	Africa	-0.004	-0.157	0.15	0.516
GBI	300 km	original	Australia	0.021	-0.234	0.27	0.627
GBI	300 km	original	C America	0.028	-0.129	0.192	0.637
GBI	300 km	original	N-C Asia	0.164	-0.056	0.388	0.907
GBI	300 km	original	New Guinea and Oceania	0.071	-0.063	0.209	0.806
GBI	300 km	original	S America	0.057	-0.09	0.2	0.747

GBI	300 km	original	S/SE Asia	0.099	-0.029	0.223	0.901
GBI	300 km	original	W and SW Eurasia	0.006	-0.182	0.182	0.575
GBI	500 km	original	Africa	0	-0.153	0.16	0.526
GBI	500 km	original	Australia	0.061	-0.207	0.334	0.703
GBI	500 km	original	C America	0.051	-0.108	0.201	0.716
GBI	500 km	original	E N America	0.059	-0.209	0.331	0.7
GBI	500 km	original	N-C Asia	0.257	0.01	0.501	0.978
GBI	500 km	original	New Guinea and Oceania	0.097	-0.065	0.257	0.844
GBI	500 km	original	S America	0.064	-0.081	0.206	0.774
GBI	500 km	original	S/SE Asia	0.115	-0.011	0.236	0.935
GBI	500 km	original	W and SW Eurasia	0.056	-0.126	0.235	0.721
TLI	300 km	jittered	Africa	0.044	-0.021	0.113	0.852
TLI	300 km	jittered	Australia	0.056	-0.03	0.149	0.865
TLI	300 km	jittered	C America	0.054	-0.019	0.126	0.886
TLI	300 km	jittered	E N America	0.061	-0.024	0.15	0.886
TLI	300 km	jittered	N-C Asia	0.07	-0.008	0.145	0.937
TLI	300 km	jittered	New Guinea and Oceania	0.06	-0.008	0.13	0.919
TLI	300 km	jittered	S America	0.064	0.001	0.127	0.948
TLI	300 km	jittered	S/SE Asia	0.071	0.008	0.138	0.964
TLI	300 km	jittered	W and SW Eurasia	0.057	-0.015	0.132	0.895
TLI	300 km	jittered	W N America	0.062	-0.021	0.147	0.897
TLI	500 km	jittered	Africa	0.072	-0.016	0.16	0.9
TLI	500 km	jittered	Australia	0.084	-0.031	0.202	0.891
TLI	500 km	jittered	C America	0.1	0.009	0.191	0.962
TLI	500 km	jittered	E N America	0.084	-0.02	0.195	0.901
TLI	500 km	jittered	N-C Asia	0.125	0.024	0.231	0.984
TLI	500 km	jittered	New Guinea and Oceania	0.078	-0.007	0.167	0.921
TLI	500 km	jittered	S America	0.081	-0.001	0.166	0.937
TLI	500 km	jittered	S/SE Asia	0.11	0.031	0.187	0.989
TLI	500 km	jittered	W and SW Eurasia	0.085	-0.011	0.183	0.919
TLI	500 km	jittered	W N America	0.095	-0.007	0.203	0.932
GBI	300 km	jittered	Africa	-0.042	-0.131	0.045	0.774

GBI	300 km	jittered	Australia	0.004	-0.152	0.161	0.548
GBI	300 km	jittered	C America	0.042	-0.064	0.153	0.731
GBI	300 km	jittered	E N America	0.012	-0.125	0.167	0.567
GBI	300 km	jittered	N-C Asia	0.064	-0.054	0.183	0.803
GBI	300 km	jittered	New Guinea and Oceania	0.001	-0.093	0.093	0.512
GBI	300 km	jittered	S America	0.02	-0.065	0.104	0.647
GBI	300 km	jittered	S/SE Asia	0.044	-0.044	0.126	0.792
GBI	300 km	jittered	W and SW Eurasia	-0.014	-0.114	0.089	0.569
GBI	500 km	jittered	Africa	-0.031	-0.164	0.1	0.625
GBI	500 km	jittered	Australia	0.034	-0.187	0.255	0.652
GBI	500 km	jittered	C America	0.071	-0.07	0.218	0.797
GBI	500 km	jittered	E N America	0.053	-0.106	0.205	0.729
GBI	500 km	jittered	N-C Asia	0.168	0.004	0.344	0.96
GBI	500 km	jittered	New Guinea and Oceania	0.04	-0.106	0.181	0.689
GBI	500 km	jittered	S America	0.088	-0.032	0.208	0.883
GBI	500 km	jittered	S/SE Asia	0.107	0.005	0.213	0.954
GBI	500 km	jittered	W and SW Eurasia	0.005	-0.151	0.162	0.559

Table S2.

Posterior probabilities for the effect of F for each feature (random slopes) at each geographical resolution and for each dataset, both for the analyses using the original and jittered coordinates. Note that this table is also available as a .csv file on the OSF repository (https://osf.io/2qgje). In the .csv table, feature definitions and linguistic domain attributions (as per reference (3)), as well as morphological complexity status are also provided.

Dataset	Diameter	Feature	Coordinates	Estimate	89% HPDI (lower)	89% HPDI (upper)	Posterior probability
TLI	300 km	TLI1055	original	-1.398	-1.684	-1.091	1
TLI	300 km	TLI0855	original	-1.083	-1.497	-0.673	1
TLI	300 km	TLI1066	original	-0.835	-1.108	-0.547	1
TLI	300 km	TLI1039	original	-0.637	-1.003	-0.251	0.997
TLI	300 km	TLI0670	original	-0.63	-1.085	-0.192	0.988
TLI	300 km	TLI0612	original	-0.572	-0.855	-0.297	0.999
TLI	300 km	TLI1016	original	-0.566	-0.919	-0.22	0.996
TLI	300 km	TLI0578	original	-0.558	-0.893	-0.205	0.996
TLI	300 km	TLI1063	original	-0.531	-1.04	-0.027	0.955
TLI	300 km	TLI1060	original	-0.518	-0.776	-0.268	0.999
TLI	300 km	TLI0615	original	-0.502	-0.717	-0.291	1
TLI	300 km	TLI0102	original	-0.496	-1.022	-0.005	0.941
TLI	300 km	TLI1036	original	-0.477	-0.895	-0.047	0.964
TLI	300 km	TLI1103	original	-0.471	-0.703	-0.23	0.999
TLI	300 km	TLI0630	original	-0.462	-0.673	-0.237	1
TLI	300 km	TLI1049	original	-0.436	-0.652	-0.223	0.999
TLI	300 km	TLI0908	original	-0.434	-0.867	-0.002	0.946
TLI	300 km	TLI0856	original	-0.409	-0.883	0.08	0.913
TLI	300 km	TLI0635	original	-0.404	-0.626	-0.186	0.998
TLI	300 km	TLI0966	original	-0.381	-0.71	-0.065	0.969
TLI	300 km	TLI0453	original	-0.375	-0.601	-0.142	0.995
TLI	300 km	TLI0976	original	-0.352	-0.73	0.024	0.932
TLI	300 km	TLI0861	original	-0.349	-0.746	0.059	0.92
TLI	300 km	TLI0893	original	-0.334	-0.627	-0.049	0.966
TLI	300 km	TLI0979	original	-0.327	-0.547	-0.107	0.991
TLI	300 km	TLI1110	original	-0.325	-0.688	0.026	0.927
TLI	300 km	TLI0081	original	-0.316	-0.881	0.23	0.82
TLI	300 km	TLI1085	original	-0.316	-0.731	0.089	0.89
TLI	300 km	TLI1111	original	-0.316	-0.665	0.043	0.924
TLI	300 km	TLI1076	original	-0.294	-0.702	0.094	0.88
TLI	300 km	TLI0975	original	-0.291	-0.826	0.233	0.81

TLI	300 km	TLI0954	original	-0.287	-0.677	0.099	0.881
TLI	300 km	TLI1069	original	-0.278	-0.517	-0.041	0.969
TLI	300 km	TLI1011	original	-0.277	-0.732	0.148	0.844
TLI	300 km	TLI1029	original	-0.272	-0.783	0.248	0.802
TLI	300 km	TLI0631	original	-0.269	-0.519	-0.025	0.959
TLI	300 km	TLI1104	original	-0.264	-0.534	-0.001	0.943
TLI	300 km	TLI1012	original	-0.244	-0.744	0.265	0.779
TLI	300 km	TLI0608	original	-0.242	-0.452	-0.039	0.969
TLI	300 km	TLI1028	original	-0.239	-0.591	0.116	0.858
TLI	300 km	TLI1065	original	-0.238	-0.459	-0.013	0.957
TLI	300 km	TLI1099	original	-0.237	-0.568	0.11	0.868
TLI	300 km	TLI0919	original	-0.236	-0.815	0.34	0.744
TLI	300 km	TLI0844	original	-0.227	-0.708	0.291	0.765
TLI	300 km	TLI0804	original	-0.226	-0.601	0.135	0.838
TLI	300 km	TLI1079	original	-0.226	-0.665	0.248	0.784
TLI	300 km	TLI0886	original	-0.224	-0.799	0.344	0.734
TLI	300 km	TLI0957	original	-0.22	-0.626	0.194	0.804
TLI	300 km	TLI1050	original	-0.208	-0.439	0.024	0.924
TLI	300 km	TLI0580	original	-0.208	-0.743	0.338	0.73
TLI	300 km	TLI1057	original	-0.201	-0.602	0.206	0.787
TLI	300 km	TLI0956	original	-0.2	-0.628	0.238	0.77
TLI	300 km	TLI0140	original	-0.199	-0.65	0.215	0.768
TLI	300 km	TLI1083	original	-0.199	-0.779	0.404	0.705
TLI	300 km	TLI0933	original	-0.191	-0.584	0.237	0.773
TLI	300 km	TLI0874	original	-0.188	-0.649	0.297	0.737
TLI	300 km	TLI0374	original	-0.173	-0.651	0.297	0.72
TLI	300 km	TLI0984	original	-0.172	-0.662	0.323	0.713
TLI	300 km	TLI1089	original	-0.164	-0.611	0.287	0.72
TLI	300 km	TLI0035	original	-0.161	-0.409	0.079	0.855
TLI	300 km	TLI1117	original	-0.16	-0.687	0.389	0.682
TLI	300 km	TLI1062	original	-0.156	-0.345	0.025	0.912
TLI	300 km	TLI0371	original	-0.153	-0.627	0.322	0.695
TLI	300 km	TLI0964	original	-0.149	-0.495	0.172	0.764
TLI	300 km	TLI0512	original	-0.144	-0.63	0.329	0.684
TLI	300 km	TLI0518	original	-0.142	-0.553	0.283	0.706
TLI	300 km	TLI1023	original	-0.131	-0.684	0.437	0.646
TLI	300 km	TLI0096	original	-0.127	-0.579	0.321	0.673
TLI	300 km	TLI1116	original	-0.127	-0.515	0.263	0.7
TLI	300 km	TLI0930	original	-0.124	-0.336	0.081	0.832

TLI	300 km	TLI0044	original	-0.121	-0.476	0.237	0.706
TLI	300 km	TLI0107	original	-0.119	-0.57	0.325	0.667
TLI	300 km	TLI0388	original	-0.118	-0.622	0.4	0.646
TLI	300 km	TLI0906	original	-0.106	-0.512	0.294	0.663
TLI	300 km	TLI1094	original	-0.106	-0.615	0.404	0.633
TLI	300 km	TLI0400	original	-0.106	-0.611	0.35	0.636
TLI	300 km	TLI1075	original	-0.106	-0.353	0.133	0.757
TLI	300 km	TLI0624	original	-0.102	-0.344	0.149	0.745
TLI	300 km	TLI0951	original	-0.096	-0.595	0.429	0.619
TLI	300 km	TLI0965	original	-0.093	-0.408	0.231	0.678
TLI	300 km	TLI0902	original	-0.093	-0.632	0.405	0.613
TLI	300 km	TLI1000	original	-0.091	-0.494	0.314	0.64
TLI	300 km	TLI0164	original	-0.09	-0.555	0.38	0.618
TLI	300 km	TLI1098	original	-0.089	-0.384	0.211	0.682
TLI	300 km	TLI0912	original	-0.084	-0.659	0.467	0.594
TLI	300 km	TLI0910	original	-0.077	-0.632	0.48	0.589
TLI	300 km	TLI0867	original	-0.074	-0.405	0.267	0.636
TLI	300 km	TLI1040	original	-0.071	-0.658	0.521	0.576
TLI	300 km	TLI0440	original	-0.056	-0.646	0.546	0.561
TLI	300 km	TLI1106	original	-0.051	-0.397	0.294	0.593
TLI	300 km	TLI1009	original	-0.048	-0.591	0.509	0.556
TLI	300 km	TLI0880	original	-0.041	-0.587	0.485	0.547
TLI	300 km	TLI0848	original	-0.04	-0.285	0.204	0.602
TLI	300 km	TLI0960	original	-0.038	-0.364	0.271	0.574
TLI	300 km	TLI1041	original	-0.034	-0.611	0.549	0.537
TLI	300 km	TLI0519	original	-0.033	-0.542	0.46	0.542
TLI	300 km	TLI1004	original	-0.03	-0.349	0.288	0.556
TLI	300 km	TLI0629	original	-0.029	-0.193	0.134	0.61
TLI	300 km	TLI1090	original	-0.027	-0.259	0.2	0.575
TLI	300 km	TLI0875	original	-0.026	-0.46	0.432	0.536
TLI	300 km	TLI0668	original	-0.023	-0.28	0.23	0.559
TLI	300 km	TLI0852	original	-0.015	-0.396	0.352	0.525
TLI	300 km	TLI0674	original	-0.011	-0.267	0.249	0.527
TLI	300 km	TLI0853	original	-0.003	-0.468	0.452	0.507
TLI	300 km	TLI1014	original	-0.002	-0.378	0.377	0.503
TLI	300 km	TLI0891	original	-0.002	-0.287	0.28	0.501
TLI	300 km	TLI0973	original	-0.001	-0.51	0.508	0.501
TLI	300 km	TLI0931	original	0.001	-0.536	0.548	0.502
TLI	300 km	TLI1026	original	0.007	-0.492	0.505	0.508

TLI	300 km	TLI0937	original	0.007	-0.387	0.425	0.513
TLI	300 km	TLI0974	original	0.007	-0.52	0.534	0.505
TLI	300 km	TLI0953	original	0.009	-0.464	0.486	0.511
TLI	300 km	TLI0879	original	0.009	-0.548	0.606	0.511
TLI	300 km	TLI0839	original	0.01	-0.368	0.369	0.515
TLI	300 km	TLI0970	original	0.013	-0.553	0.598	0.517
TLI	300 km	TLI0890	original	0.014	-0.318	0.331	0.528
TLI	300 km	TLI0792	original	0.015	-0.478	0.496	0.519
TLI	300 km	TLI1003	original	0.018	-0.506	0.566	0.52
TLI	300 km	TLI1084	original	0.018	-0.387	0.407	0.526
TLI	300 km	TLI0978	original	0.022	-0.18	0.222	0.569
TLI	300 km	TLI0849	original	0.024	-0.374	0.404	0.541
TLI	300 km	TLI0866	original	0.03	-0.285	0.344	0.559
TLI	300 km	TLI0887	original	0.032	-0.542	0.572	0.536
TLI	300 km	TLI1035	original	0.032	-0.458	0.521	0.543
TLI	300 km	TLI1074	original	0.034	-0.262	0.326	0.574
TLI	300 km	TLI0909	original	0.034	-0.618	0.66	0.536
TLI	300 km	TLI0967	original	0.036	-0.372	0.434	0.558
TLI	300 km	TLI1042	original	0.037	-0.549	0.643	0.538
TLI	300 km	TLI0945	original	0.04	-0.498	0.565	0.549
TLI	300 km	TLI0987	original	0.041	-0.384	0.478	0.559
TLI	300 km	TLI0899	original	0.042	-0.508	0.566	0.549
TLI	300 km	TLI1037	original	0.046	-0.491	0.585	0.554
TLI	300 km	TLI0878	original	0.046	-0.419	0.526	0.563
TLI	300 km	TLI0845	original	0.048	-0.345	0.444	0.577
TLI	300 km	TLI0885	original	0.048	-0.512	0.624	0.554
TLI	300 km	TLI1118	original	0.049	-0.485	0.584	0.559
TLI	300 km	TLI0850	original	0.05	-0.474	0.57	0.56
TLI	300 km	TLI0916	original	0.05	-0.521	0.599	0.558
TLI	300 km	TLI1059	original	0.054	-0.258	0.372	0.607
TLI	300 km	TLI1088	original	0.061	-0.239	0.379	0.622
TLI	300 km	TLI0597	original	0.064	-0.581	0.735	0.563
TLI	300 km	TLI0999	original	0.066	-0.371	0.532	0.592
TLI	300 km	TLI0515	original	0.066	-0.421	0.58	0.581
TLI	300 km	TLI0920	original	0.066	-0.508	0.656	0.573
TLI	300 km	TLI1072	original	0.067	-0.409	0.533	0.592
TLI	300 km	TLI0905	original	0.068	-0.311	0.447	0.613
TLI	300 km	TLI1113	original	0.07	-0.186	0.327	0.669
TLI	300 km	TLI1005	original	0.075	-0.321	0.468	0.619

TLI	300 km	TLI0943	original	0.075	-0.453	0.616	0.589
TLI	300 km	TLI0738	original	0.075	-0.089	0.233	0.774
TLI	300 km	TLI0672	original	0.077	-0.272	0.443	0.635
TLI	300 km	TLI1019	original	0.078	-0.415	0.553	0.602
TLI	300 km	TLI0915	original	0.079	-0.334	0.496	0.621
TLI	300 km	TLI0654	original	0.084	-0.354	0.522	0.622
TLI	300 km	TLI0370	original	0.086	-0.401	0.6	0.608
TLI	300 km	TLI0907	original	0.088	-0.362	0.544	0.618
TLI	300 km	TLI0865	original	0.089	-0.43	0.609	0.607
TLI	300 km	TLI0771	original	0.09	-0.478	0.638	0.6
TLI	300 km	TLI0846	original	0.09	-0.221	0.394	0.681
TLI	300 km	TLI0155	original	0.095	-0.357	0.551	0.631
TLI	300 km	TLI1015	original	0.095	-0.082	0.273	0.805
TLI	300 km	TLI0888	original	0.1	-0.434	0.604	0.623
TLI	300 km	TLI0430	original	0.102	-0.449	0.606	0.622
TLI	300 km	TLI0892	original	0.103	-0.234	0.446	0.686
TLI	300 km	TLI0894	original	0.103	-0.11	0.324	0.777
TLI	300 km	TLI0959	original	0.106	-0.394	0.592	0.634
TLI	300 km	TLI1002	original	0.106	-0.37	0.615	0.634
TLI	300 km	TLI1053	original	0.107	-0.14	0.358	0.756
TLI	300 km	TLI1047	original	0.108	-0.323	0.574	0.645
TLI	300 km	TLI0914	original	0.109	-0.254	0.465	0.687
TLI	300 km	TLI0514	original	0.114	-0.373	0.613	0.647
TLI	300 km	TLI1044	original	0.118	-0.175	0.406	0.742
TLI	300 km	TLI0513	original	0.119	-0.26	0.495	0.693
TLI	300 km	TLI1082	original	0.12	-0.309	0.551	0.671
TLI	300 km	TLI0868	original	0.124	-0.46	0.736	0.63
TLI	300 km	TLI0613	original	0.127	-0.105	0.367	0.805
TLI	300 km	TLI1097	original	0.128	-0.292	0.542	0.688
TLI	300 km	TLI0982	original	0.13	-0.153	0.42	0.765
TLI	300 km	TLI0376	original	0.132	-0.339	0.631	0.665
TLI	300 km	TLI0924	original	0.133	-0.326	0.611	0.673
TLI	300 km	TLI0932	original	0.134	-0.43	0.685	0.649
TLI	300 km	TLI0991	original	0.135	-0.3	0.591	0.683
TLI	300 km	TLI0360	original	0.137	-0.341	0.611	0.677
TLI	300 km	TLI0766	original	0.138	-0.275	0.546	0.705
TLI	300 km	TLI1077	original	0.138	-0.061	0.339	0.866
TLI	300 km	TLI0925	original	0.143	0.01	0.283	0.952
TLI	300 km	TLI1073	original	0.144	-0.057	0.337	0.88

TLI	300 km	TLI0901	original	0.145	-0.378	0.661	0.676
TLI	300 km	TLI0517	original	0.145	-0.373	0.652	0.674
TLI	300 km	TLI0640	original	0.146	-0.108	0.404	0.819
TLI	300 km	TLI1092	original	0.148	-0.262	0.565	0.717
TLI	300 km	TLI0180	original	0.148	-0.278	0.575	0.71
TLI	300 km	TLI0988	original	0.154	-0.224	0.536	0.742
TLI	300 km	TLI0998	original	0.158	-0.306	0.604	0.714
TLI	300 km	TLI1018	original	0.159	-0.375	0.725	0.679
TLI	300 km	TLI0972	original	0.162	-0.412	0.734	0.679
TLI	300 km	TLI1061	original	0.164	-0.053	0.382	0.885
TLI	300 km	TLI0552	original	0.165	-0.191	0.531	0.765
TLI	300 km	TLI0898	original	0.166	-0.116	0.445	0.828
TLI	300 km	TLI0990	original	0.166	-0.357	0.706	0.695
TLI	300 km	TLI0938	original	0.169	-0.336	0.676	0.706
TLI	300 km	TLI1078	original	0.169	-0.102	0.453	0.836
TLI	300 km	TLI1043	original	0.17	-0.326	0.677	0.707
TLI	300 km	TLI0904	original	0.174	-0.287	0.619	0.732
TLI	300 km	TLI0992	original	0.175	-0.318	0.695	0.712
TLI	300 km	TLI0378	original	0.177	-0.321	0.677	0.716
TLI	300 km	TLI0882	original	0.178	-0.3	0.67	0.721
TLI	300 km	TLI1071	original	0.18	-0.3	0.663	0.728
TLI	300 km	TLI0889	original	0.183	-0.196	0.564	0.778
TLI	300 km	TLI0971	original	0.185	-0.39	0.753	0.698
TLI	300 km	TLI1022	original	0.191	-0.204	0.584	0.779
TLI	300 km	TLI0642	original	0.197	0.008	0.393	0.948
TLI	300 km	TLI0913	original	0.198	-0.164	0.543	0.816
TLI	300 km	TLI1021	original	0.204	-0.338	0.74	0.728
TLI	300 km	TLI0005	original	0.208	-0.246	0.672	0.766
TLI	300 km	TLI0682	original	0.209	-0.014	0.439	0.93
TLI	300 km	TLI1109	original	0.212	-0.276	0.718	0.749
TLI	300 km	TLI0900	original	0.213	-0.323	0.743	0.738
TLI	300 km	TLI1067	original	0.213	-0.257	0.665	0.771
TLI	300 km	TLI1038	original	0.214	-0.315	0.765	0.737
TLI	300 km	TLI1102	original	0.217	-0.155	0.608	0.817
TLI	300 km	TLI0401	original	0.221	-0.254	0.705	0.768
TLI	300 km	TLI0847	original	0.223	-0.277	0.74	0.756
TLI	300 km	TLI0877	original	0.228	-0.277	0.722	0.767
TLI	300 km	TLI0911	original	0.23	-0.33	0.802	0.742
TLI	300 km	TLI0983	original	0.232	-0.251	0.746	0.774

TLI	300 km	TLI1013	original	0.237	-0.192	0.67	0.808
TLI	300 km	TLI1031	original	0.237	-0.246	0.715	0.784
TLI	300 km	TLI0616	original	0.239	0.065	0.415	0.984
TLI	300 km	TLI0934	original	0.242	-0.178	0.634	0.829
TLI	300 km	TLI0751	original	0.253	0.06	0.445	0.981
TLI	300 km	TLI1024	original	0.259	-0.219	0.74	0.807
TLI	300 km	TLI1115	original	0.26	-0.209	0.708	0.818
TLI	300 km	TLI0995	original	0.261	-0.251	0.752	0.797
TLI	300 km	TLI0858	original	0.261	-0.32	0.841	0.763
TLI	300 km	TLI1081	original	0.267	-0.03	0.557	0.928
TLI	300 km	TLI0917	original	0.275	-0.253	0.818	0.794
TLI	300 km	TLI1032	original	0.277	-0.205	0.768	0.817
TLI	300 km	TLI0381	original	0.278	-0.18	0.728	0.838
TLI	300 km	TLI0549	original	0.28	-0.136	0.709	0.857
TLI	300 km	TLI0921	original	0.288	0.048	0.54	0.968
TLI	300 km	TLI0860	original	0.292	0.024	0.564	0.958
TLI	300 km	TLI1105	original	0.296	-0.199	0.786	0.833
TLI	300 km	TLI0981	original	0.298	-0.232	0.815	0.819
TLI	300 km	TLI0929	original	0.3	0.072	0.533	0.981
TLI	300 km	TLI0918	original	0.302	-0.237	0.846	0.813
TLI	300 km	TLI0935	original	0.304	-0.159	0.767	0.853
TLI	300 km	TLI0560	original	0.305	-0.209	0.849	0.824
TLI	300 km	TLI0947	original	0.305	-0.197	0.796	0.838
TLI	300 km	TLI0940	original	0.305	-0.195	0.798	0.838
TLI	300 km	TLI1030	original	0.309	-0.235	0.834	0.823
TLI	300 km	TLI1045	original	0.311	-0.097	0.712	0.89
TLI	300 km	TLI1064	original	0.318	-0.109	0.739	0.883
TLI	300 km	TLI0962	original	0.319	-0.085	0.728	0.895
TLI	300 km	TLI0843	original	0.322	-0.201	0.836	0.84
TLI	300 km	TLI0398	original	0.323	-0.158	0.822	0.852
TLI	300 km	TLI1091	original	0.323	0.13	0.513	0.996
TLI	300 km	TLI1068	original	0.325	0.176	0.475	0.999
TLI	300 km	TLI0980	original	0.328	-0.057	0.715	0.914
TLI	300 km	TLI0985	original	0.328	-0.22	0.894	0.828
TLI	300 km	TLI0120	original	0.33	0.021	0.643	0.956
TLI	300 km	TLI1020	original	0.332	-0.024	0.709	0.927
TLI	300 km	TLI0963	original	0.335	-0.031	0.69	0.93
TLI	300 km	TLI0952	original	0.336	0.017	0.662	0.952
TLI	300 km	TLI1080	original	0.341	-0.045	0.73	0.921

TLI	300 km	TLI0857	original	0.342	-0.151	0.848	0.863
TLI	300 km	TLI0872	original	0.344	-0.029	0.726	0.928
TLI	300 km	TLI0871	original	0.345	-0.069	0.762	0.906
TLI	300 km	TLI0859	original	0.345	-0.169	0.851	0.862
TLI	300 km	TLI1070	original	0.348	-0.05	0.73	0.923
TLI	300 km	TLI1096	original	0.348	-0.167	0.83	0.866
TLI	300 km	TLI1046	original	0.351	-0.074	0.762	0.909
TLI	300 km	TLI1093	original	0.353	-0.107	0.82	0.887
TLI	300 km	TLI0873	original	0.354	-0.144	0.843	0.874
TLI	300 km	TLI0994	original	0.359	-0.12	0.821	0.889
TLI	300 km	TLI0986	original	0.362	-0.192	0.904	0.857
TLI	300 km	TLI0636	original	0.365	0.133	0.611	0.992
TLI	300 km	TLI0364	original	0.365	-0.093	0.837	0.896
TLI	300 km	TLI1058	original	0.368	0.044	0.701	0.964
TLI	300 km	TLI0903	original	0.371	-0.183	0.922	0.86
TLI	300 km	TLI0996	original	0.378	-0.077	0.82	0.912
TLI	300 km	TLI1010	original	0.398	0.04	0.764	0.96
TLI	300 km	TLI0179	original	0.408	-0.022	0.835	0.937
TLI	300 km	TLI0968	original	0.414	0	0.811	0.949
TLI	300 km	TLI1056	original	0.415	-0.007	0.833	0.944
TLI	300 km	TLI0936	original	0.418	-0.005	0.831	0.945
TLI	300 km	TLI0673	original	0.418	0.103	0.77	0.978
TLI	300 km	TLI0993	original	0.421	-0.069	0.935	0.91
TLI	300 km	TLI1025	original	0.422	-0.063	0.892	0.922
TLI	300 km	TLI1087	original	0.434	-0.02	0.923	0.93
TLI	300 km	TLI0977	original	0.436	0.027	0.825	0.959
TLI	300 km	TLI0801	original	0.437	-0.005	0.88	0.943
TLI	300 km	TLI1008	original	0.439	0.009	0.899	0.944
TLI	300 km	TLI0617	original	0.453	0.209	0.695	0.998
TLI	300 km	TLI0842	original	0.457	-0.058	0.962	0.924
TLI	300 km	TLI0896	original	0.463	0.219	0.717	0.998
TLI	300 km	TLI0604	original	0.463	-0.067	1	0.918
TLI	300 km	TLI0884	original	0.463	-0.037	0.945	0.935
TLI	300 km	TLI1027	original	0.466	-0.066	0.986	0.922
TLI	300 km	TLI0790	original	0.473	0.018	0.941	0.95
TLI	300 km	TLI0895	original	0.474	0.247	0.694	1
TLI	300 km	TLI0841	original	0.478	0.056	0.876	0.969
TLI	300 km	TLI0383	original	0.49	0.012	0.965	0.95
TLI	300 km	TLI0598	original	0.491	0.063	0.901	0.969

TLI	300 km	TLI0589	original	0.495	0.132	0.866	0.985
TLI	300 km	TLI0596	original	0.498	0.092	0.895	0.977
TLI	300 km	TLI1017	original	0.504	-0.007	0.98	0.95
TLI	300 km	TLI1112	original	0.509	0.171	0.829	0.993
TLI	300 km	TLI0881	original	0.52	-0.035	1.049	0.938
TLI	300 km	TLI0582	original	0.527	0.16	0.907	0.988
TLI	300 km	TLI0592	original	0.538	0.116	0.957	0.98
TLI	300 km	TLI0431	original	0.543	0.085	0.992	0.973
TLI	300 km	TLI1107	original	0.554	0.275	0.835	0.999
TLI	300 km	TLI0392	original	0.577	0.065	1.102	0.962
TLI	300 km	TLI1100	original	0.58	0.301	0.848	1
TLI	300 km	TLI1101	original	0.583	0.193	0.979	0.991
TLI	300 km	TLI0373	original	0.597	0.055	1.109	0.966
TLI	300 km	TLI1033	original	0.604	0.246	0.957	0.997
TLI	300 km	TLI1006	original	0.614	0.159	1.045	0.986
TLI	300 km	TLI0621	original	0.621	0.446	0.799	1
TLI	300 km	TLI0923	original	0.678	0.285	1.079	0.997
TLI	300 km	TLI0840	original	0.681	0.197	1.173	0.988
TLI	300 km	TLI0961	original	0.721	0.204	1.25	0.986
TLI	300 km	TLI0570	original	0.722	0.257	1.178	0.995
TLI	300 km	TLI1051	original	0.764	0.505	1.008	1
TLI	300 km	TLI0989	original	0.807	0.301	1.277	0.997
TLI	300 km	TLI1007	original	0.811	0.363	1.264	0.998
TLI	300 km	TLI0955	original	0.929	0.597	1.27	1
TLI	300 km	TLI0939	original	0.979	0.524	1.425	1
TLI	300 km	TLI0958	original	1.02	0.577	1.456	1
TLI	300 km	TLI0559	original	1.048	0.61	1.474	1
TLI	300 km	TLI1052	original	1.074	0.807	1.343	1
TLI	300 km	TLI0928	original	1.095	0.651	1.526	1
TLI	500 km	TLI1055	original	-1.319	-1.644	-0.989	1
TLI	500 km	TLI0855	original	-1.122	-1.472	-0.778	1
TLI	500 km	TLI1066	original	-0.819	-1.095	-0.542	1
TLI	500 km	TLI0578	original	-0.677	-0.971	-0.37	1
TLI	500 km	TLI1016	original	-0.666	-1.002	-0.319	0.999
TLI	500 km	TLI0612	original	-0.624	-0.877	-0.368	1
TLI	500 km	TLI1036	original	-0.608	-0.991	-0.232	0.995
TLI	500 km	TLI1039	original	-0.601	-0.856	-0.34	1
TLI	500 km	TLI0615	original	-0.569	-0.766	-0.373	1
TLI	500 km	TLI1063	original	-0.556	-1.033	-0.087	0.969

TLI	500 km	TLI1060	original	-0.556	-0.797	-0.314	1
TLI	500 km	TLI0453	original	-0.504	-0.687	-0.316	1
TLI	500 km	TLI0102	original	-0.502	-0.945	-0.043	0.962
TLI	500 km	TLI1103	original	-0.494	-0.72	-0.281	1
TLI	500 km	TLI0861	original	-0.47	-0.787	-0.156	0.993
TLI	500 km	TLI0512	original	-0.444	-0.842	-0.059	0.965
TLI	500 km	TLI0635	original	-0.422	-0.617	-0.219	1
TLI	500 km	TLI1111	original	-0.416	-0.646	-0.177	0.998
TLI	500 km	TLI1028	original	-0.415	-0.685	-0.133	0.992
TLI	500 km	TLI1049	original	-0.4	-0.61	-0.196	0.999
TLI	500 km	TLI0954	original	-0.398	-0.755	-0.025	0.959
TLI	500 km	TLI0630	original	-0.396	-0.57	-0.212	1
TLI	500 km	TLI0518	original	-0.392	-0.784	0.004	0.945
TLI	500 km	TLI0966	original	-0.385	-0.704	-0.066	0.974
TLI	500 km	TLI0976	original	-0.38	-0.79	-0.001	0.939
TLI	500 km	TLI0979	original	-0.376	-0.552	-0.197	1
TLI	500 km	TLI0908	original	-0.375	-0.78	0.041	0.928
TLI	500 km	TLI0974	original	-0.36	-0.861	0.18	0.866
TLI	500 km	TLI0957	original	-0.359	-0.73	0.021	0.938
TLI	500 km	TLI0670	original	-0.356	-0.774	0.046	0.919
TLI	500 km	TLI1069	original	-0.352	-0.6	-0.094	0.987
TLI	500 km	TLI1057	original	-0.343	-0.768	0.073	0.903
TLI	500 km	TLI1079	original	-0.341	-0.712	0.018	0.933
TLI	500 km	TLI0844	original	-0.339	-0.828	0.139	0.868
TLI	500 km	TLI1099	original	-0.338	-0.632	-0.038	0.965
TLI	500 km	TLI0893	original	-0.309	-0.546	-0.076	0.981
TLI	500 km	TLI1085	original	-0.307	-0.728	0.136	0.875
TLI	500 km	TLI0081	original	-0.292	-0.859	0.261	0.798
TLI	500 km	TLI0608	original	-0.289	-0.478	-0.095	0.993
TLI	500 km	TLI0631	original	-0.285	-0.522	-0.067	0.977
TLI	500 km	TLI1110	original	-0.284	-0.642	0.071	0.9
TLI	500 km	TLI0371	original	-0.282	-0.728	0.154	0.846
TLI	500 km	TLI0956	original	-0.256	-0.663	0.133	0.847
TLI	500 km	TLI0856	original	-0.241	-0.638	0.167	0.833
TLI	500 km	TLI1083	original	-0.241	-0.837	0.337	0.744
TLI	500 km	TLI0164	original	-0.24	-0.63	0.162	0.834
TLI	500 km	TLI1023	original	-0.228	-0.75	0.32	0.75
TLI	500 km	TLI1062	original	-0.227	-0.394	-0.056	0.983
TLI	500 km	TLI0919	original	-0.225	-0.783	0.356	0.734

TLI	500 km	TLI1029	original	-0.224	-0.659	0.24	0.789
TLI	500 km	TLI0096	original	-0.223	-0.662	0.2	0.793
TLI	500 km	TLI0933	original	-0.214	-0.576	0.168	0.823
TLI	500 km	TLI0804	original	-0.209	-0.462	0.038	0.91
TLI	500 km	TLI0580	original	-0.207	-0.65	0.247	0.769
TLI	500 km	TLI1104	original	-0.197	-0.446	0.055	0.896
TLI	500 km	TLI0140	original	-0.192	-0.561	0.181	0.794
TLI	500 km	TLI0915	original	-0.191	-0.577	0.217	0.774
TLI	500 km	TLI0891	original	-0.182	-0.469	0.096	0.849
TLI	500 km	TLI0930	original	-0.179	-0.399	0.041	0.902
TLI	500 km	TLI0792	original	-0.177	-0.565	0.207	0.772
TLI	500 km	TLI1012	original	-0.172	-0.668	0.33	0.712
TLI	500 km	TLI1011	original	-0.171	-0.571	0.202	0.759
TLI	500 km	TLI0912	original	-0.169	-0.683	0.374	0.696
TLI	500 km	TLI0107	original	-0.168	-0.551	0.219	0.761
TLI	500 km	TLI0388	original	-0.167	-0.626	0.331	0.714
TLI	500 km	TLI1019	original	-0.166	-0.601	0.272	0.731
TLI	500 km	TLI1065	original	-0.165	-0.359	0.03	0.914
TLI	500 km	TLI1050	original	-0.163	-0.358	0.039	0.904
TLI	500 km	TLI0597	original	-0.162	-0.804	0.476	0.655
TLI	500 km	TLI0886	original	-0.159	-0.699	0.414	0.673
TLI	500 km	TLI0879	original	-0.157	-0.702	0.38	0.679
TLI	500 km	TLI1094	original	-0.156	-0.636	0.341	0.696
TLI	500 km	TLI0984	original	-0.149	-0.583	0.275	0.707
TLI	500 km	TLI0440	original	-0.143	-0.683	0.388	0.666
TLI	500 km	TLI0902	original	-0.141	-0.585	0.291	0.698
TLI	500 km	TLI0975	original	-0.141	-0.594	0.311	0.692
TLI	500 km	TLI1075	original	-0.139	-0.365	0.083	0.84
TLI	500 km	TLI0960	original	-0.134	-0.439	0.183	0.754
TLI	500 km	TLI0771	original	-0.131	-0.617	0.359	0.667
TLI	500 km	TLI0892	original	-0.126	-0.444	0.191	0.737
TLI	500 km	TLI1076	original	-0.125	-0.488	0.242	0.707
TLI	500 km	TLI0999	original	-0.109	-0.529	0.301	0.667
TLI	500 km	TLI0035	original	-0.109	-0.324	0.101	0.789
TLI	500 km	TLI0867	original	-0.105	-0.403	0.18	0.717
TLI	500 km	TLI0920	original	-0.096	-0.655	0.5	0.601
TLI	500 km	TLI0374	original	-0.092	-0.528	0.358	0.631
TLI	500 km	TLI0875	original	-0.091	-0.511	0.324	0.637
TLI	500 km	TLI0519	original	-0.086	-0.541	0.342	0.62

TLI	500 km	TLI0910	original	-0.085	-0.594	0.442	0.603
TLI	500 km	TLI1089	original	-0.082	-0.44	0.301	0.638
TLI	500 km	TLI1000	original	-0.079	-0.466	0.307	0.627
TLI	500 km	TLI1115	original	-0.077	-0.534	0.36	0.611
TLI	500 km	TLI0890	original	-0.075	-0.363	0.221	0.658
TLI	500 km	TLI0674	original	-0.075	-0.311	0.167	0.691
TLI	500 km	TLI1003	original	-0.074	-0.619	0.499	0.581
TLI	500 km	TLI0514	original	-0.073	-0.489	0.34	0.61
TLI	500 km	TLI0906	original	-0.07	-0.417	0.285	0.626
TLI	500 km	TLI0400	original	-0.068	-0.519	0.377	0.597
TLI	500 km	TLI1040	original	-0.066	-0.63	0.486	0.575
TLI	500 km	TLI0880	original	-0.062	-0.593	0.45	0.572
TLI	500 km	TLI1116	original	-0.047	-0.364	0.259	0.595
TLI	500 km	TLI0964	original	-0.04	-0.364	0.298	0.578
TLI	500 km	TLI1072	original	-0.038	-0.443	0.386	0.558
TLI	500 km	TLI0848	original	-0.038	-0.188	0.115	0.656
TLI	500 km	TLI0624	original	-0.035	-0.263	0.203	0.594
TLI	500 km	TLI0044	original	-0.031	-0.357	0.297	0.563
TLI	500 km	TLI0931	original	-0.029	-0.519	0.488	0.537
TLI	500 km	TLI0970	original	-0.029	-0.559	0.52	0.538
TLI	500 km	TLI0965	original	-0.028	-0.341	0.275	0.555
TLI	500 km	TLI1117	original	-0.023	-0.533	0.502	0.531
TLI	500 km	TLI0973	original	-0.023	-0.46	0.403	0.533
TLI	500 km	TLI0967	original	-0.022	-0.365	0.308	0.545
TLI	500 km	TLI0654	original	-0.021	-0.385	0.351	0.537
TLI	500 km	TLI0987	original	-0.019	-0.483	0.414	0.528
TLI	500 km	TLI0629	original	-0.018	-0.177	0.146	0.57
TLI	500 km	TLI0945	original	-0.014	-0.474	0.459	0.52
TLI	500 km	TLI0849	original	-0.009	-0.297	0.269	0.517
TLI	500 km	TLI0937	original	-0.007	-0.415	0.352	0.514
TLI	500 km	TLI1041	original	-0.007	-0.525	0.519	0.509
TLI	500 km	TLI0899	original	-0.005	-0.477	0.502	0.505
TLI	500 km	TLI0370	original	-0.002	-0.464	0.452	0.502
TLI	500 km	TLI1009	original	0.001	-0.476	0.482	0.501
TLI	500 km	TLI0874	original	0.001	-0.417	0.413	0.504
TLI	500 km	TLI0846	original	0.004	-0.228	0.233	0.513
TLI	500 km	TLI1004	original	0.006	-0.28	0.281	0.514
TLI	500 km	TLI0909	original	0.01	-0.639	0.668	0.508
TLI	500 km	TLI1026	original	0.01	-0.438	0.461	0.516

TLI	500 km	TLI0672	original	0.011	-0.313	0.322	0.524
TLI	500 km	TLI0613	original	0.021	-0.223	0.262	0.555
TLI	500 km	TLI1098	original	0.022	-0.258	0.308	0.546
TLI	500 km	TLI0668	original	0.022	-0.157	0.212	0.579
TLI	500 km	TLI0998	original	0.023	-0.391	0.429	0.534
TLI	500 km	TLI1035	original	0.024	-0.413	0.474	0.536
TLI	500 km	TLI0850	original	0.029	-0.497	0.541	0.536
TLI	500 km	TLI1042	original	0.03	-0.499	0.561	0.535
TLI	500 km	TLI0916	original	0.031	-0.518	0.599	0.536
TLI	500 km	TLI1082	original	0.032	-0.302	0.351	0.563
TLI	500 km	TLI0738	original	0.033	-0.095	0.151	0.667
TLI	500 km	TLI0901	original	0.034	-0.483	0.568	0.544
TLI	500 km	TLI1045	original	0.035	-0.33	0.39	0.563
TLI	500 km	TLI0517	original	0.036	-0.406	0.469	0.552
TLI	500 km	TLI1044	original	0.039	-0.194	0.266	0.607
TLI	500 km	TLI0887	original	0.04	-0.506	0.592	0.546
TLI	500 km	TLI0852	original	0.04	-0.27	0.378	0.583
TLI	500 km	TLI1090	original	0.044	-0.18	0.267	0.626
TLI	500 km	TLI0953	original	0.056	-0.396	0.468	0.58
TLI	500 km	TLI1088	original	0.059	-0.171	0.297	0.654
TLI	500 km	TLI1015	original	0.06	-0.076	0.2	0.759
TLI	500 km	TLI0925	original	0.062	-0.034	0.165	0.844
TLI	500 km	TLI1037	original	0.066	-0.484	0.587	0.578
TLI	500 km	TLI0865	original	0.068	-0.455	0.567	0.585
TLI	500 km	TLI0971	original	0.068	-0.395	0.545	0.59
TLI	500 km	TLI0853	original	0.074	-0.373	0.498	0.604
TLI	500 km	TLI0905	original	0.075	-0.251	0.415	0.643
TLI	500 km	TLI0376	original	0.079	-0.391	0.549	0.606
TLI	500 km	TLI0878	original	0.08	-0.335	0.497	0.62
TLI	500 km	TLI0866	original	0.082	-0.156	0.327	0.705
TLI	500 km	TLI1118	original	0.082	-0.431	0.597	0.602
TLI	500 km	TLI0155	original	0.085	-0.311	0.495	0.634
TLI	500 km	TLI0972	original	0.088	-0.385	0.578	0.614
TLI	500 km	TLI0839	original	0.099	-0.257	0.453	0.673
TLI	500 km	TLI0640	original	0.101	-0.112	0.315	0.771
TLI	500 km	TLI0513	original	0.102	-0.225	0.417	0.694
TLI	500 km	TLI0990	original	0.102	-0.325	0.526	0.648
TLI	500 km	TLI0845	original	0.105	-0.287	0.491	0.666
TLI	500 km	TLI0988	original	0.106	-0.205	0.421	0.703

TLI	500 km	TLI0978	original	0.108	-0.06	0.278	0.846
TLI	500 km	TLI0005	original	0.109	-0.237	0.475	0.688
TLI	500 km	TLI0943	original	0.111	-0.357	0.579	0.651
TLI	500 km	TLI0515	original	0.112	-0.349	0.56	0.653
TLI	500 km	TLI1106	original	0.117	-0.156	0.409	0.745
TLI	500 km	TLI1059	original	0.118	-0.168	0.405	0.742
TLI	500 km	TLI0951	original	0.126	-0.362	0.606	0.662
TLI	500 km	TLI0907	original	0.127	-0.277	0.533	0.696
TLI	500 km	TLI1022	original	0.127	-0.263	0.491	0.705
TLI	500 km	TLI1021	original	0.132	-0.344	0.629	0.668
TLI	500 km	TLI0894	original	0.134	-0.069	0.342	0.85
TLI	500 km	TLI1071	original	0.134	-0.299	0.559	0.694
TLI	500 km	TLI0552	original	0.136	-0.166	0.438	0.763
TLI	500 km	TLI0959	original	0.138	-0.34	0.618	0.677
TLI	500 km	TLI0682	original	0.144	-0.034	0.32	0.903
TLI	500 km	TLI0992	original	0.152	-0.311	0.617	0.697
TLI	500 km	TLI1002	original	0.152	-0.247	0.548	0.728
TLI	500 km	TLI0180	original	0.152	-0.215	0.526	0.742
TLI	500 km	TLI0885	original	0.154	-0.4	0.713	0.671
TLI	500 km	TLI0888	original	0.154	-0.32	0.629	0.7
TLI	500 km	TLI0898	original	0.156	-0.077	0.398	0.853
TLI	500 km	TLI0932	original	0.158	-0.323	0.667	0.693
TLI	500 km	TLI1070	original	0.158	-0.216	0.533	0.75
TLI	500 km	TLI0360	original	0.162	-0.286	0.59	0.723
TLI	500 km	TLI1078	original	0.163	-0.061	0.391	0.876
TLI	500 km	TLI1074	original	0.165	-0.12	0.45	0.821
TLI	500 km	TLI0868	original	0.166	-0.391	0.703	0.687
TLI	500 km	TLI1113	original	0.168	-0.062	0.394	0.881
TLI	500 km	TLI1073	original	0.171	0.004	0.336	0.95
TLI	500 km	TLI1014	original	0.172	-0.122	0.473	0.821
TLI	500 km	TLI0995	original	0.173	-0.236	0.582	0.752
TLI	500 km	TLI0982	original	0.174	-0.103	0.427	0.852
TLI	500 km	TLI0766	original	0.176	-0.163	0.521	0.793
TLI	500 km	TLI0882	original	0.188	-0.278	0.616	0.751
TLI	500 km	TLI0991	original	0.188	-0.217	0.565	0.779
TLI	500 km	TLI0929	original	0.19	-0.034	0.409	0.914
TLI	500 km	TLI1024	original	0.193	-0.221	0.578	0.778
TLI	500 km	TLI1061	original	0.194	-0.002	0.382	0.945
TLI	500 km	TLI0549	original	0.197	-0.156	0.577	0.806

TLI	500 km	TLI0913	original	0.203	-0.103	0.5	0.859
TLI	500 km	TLI1018	original	0.208	-0.278	0.725	0.747
TLI	500 km	TLI0889	original	0.21	-0.147	0.568	0.827
TLI	500 km	TLI0904	original	0.211	-0.105	0.533	0.853
TLI	500 km	TLI1053	original	0.214	-0.029	0.46	0.917
TLI	500 km	TLI0921	original	0.223	0.035	0.408	0.97
TLI	500 km	TLI1097	original	0.224	-0.167	0.625	0.816
TLI	500 km	TLI1013	original	0.226	-0.148	0.57	0.843
TLI	500 km	TLI0430	original	0.227	-0.289	0.766	0.756
TLI	500 km	TLI0938	original	0.227	-0.259	0.674	0.783
TLI	500 km	TLI0642	original	0.233	0.04	0.421	0.975
TLI	500 km	TLI0935	original	0.234	-0.164	0.618	0.832
TLI	500 km	TLI0940	original	0.237	-0.211	0.679	0.802
TLI	500 km	TLI1047	original	0.238	-0.145	0.634	0.836
TLI	500 km	TLI0983	original	0.251	-0.19	0.69	0.82
TLI	500 km	TLI0963	original	0.255	-0.045	0.547	0.914
TLI	500 km	TLI0179	original	0.259	-0.136	0.636	0.857
TLI	500 km	TLI0877	original	0.259	-0.182	0.696	0.828
TLI	500 km	TLI1005	original	0.26	-0.113	0.633	0.863
TLI	500 km	TLI1081	original	0.26	-0.017	0.528	0.937
TLI	500 km	TLI0616	original	0.265	0.106	0.418	0.996
TLI	500 km	TLI1032	original	0.268	-0.131	0.672	0.856
TLI	500 km	TLI0872	original	0.272	-0.044	0.575	0.919
TLI	500 km	TLI0918	original	0.274	-0.26	0.826	0.79
TLI	500 km	TLI0917	original	0.276	-0.247	0.805	0.796
TLI	500 km	TLI0914	original	0.278	-0.042	0.581	0.922
TLI	500 km	TLI0873	original	0.279	-0.201	0.735	0.829
TLI	500 km	TLI1068	original	0.282	0.153	0.41	1
TLI	500 km	TLI0911	original	0.287	-0.263	0.832	0.8
TLI	500 km	TLI0751	original	0.287	0.11	0.472	0.994
TLI	500 km	TLI1077	original	0.29	0.056	0.504	0.982
TLI	500 km	TLI0860	original	0.295	0.088	0.496	0.99
TLI	500 km	TLI1038	original	0.298	-0.154	0.76	0.851
TLI	500 km	TLI0401	original	0.298	-0.156	0.775	0.849
TLI	500 km	TLI0924	original	0.299	-0.159	0.741	0.855
TLI	500 km	TLI0378	original	0.302	-0.142	0.763	0.857
TLI	500 km	TLI0857	original	0.304	-0.126	0.738	0.872
TLI	500 km	TLI0871	original	0.311	-0.077	0.706	0.897
TLI	500 km	TLI0934	original	0.311	-0.011	0.633	0.938

TLI	500 km	TLI0582	original	0.311	-0.022	0.636	0.934
TLI	500 km	TLI1046	original	0.317	-0.046	0.683	0.919
TLI	500 km	TLI1084	original	0.323	-0.054	0.706	0.914
TLI	500 km	TLI1058	original	0.324	0.032	0.618	0.96
TLI	500 km	TLI0952	original	0.333	0.097	0.57	0.988
TLI	500 km	TLI1102	original	0.337	-0.035	0.699	0.928
TLI	500 km	TLI1033	original	0.338	0.026	0.645	0.959
TLI	500 km	TLI0985	original	0.342	-0.189	0.864	0.85
TLI	500 km	TLI0843	original	0.342	-0.144	0.819	0.87
TLI	500 km	TLI1064	original	0.347	-0.002	0.693	0.945
TLI	500 km	TLI1109	original	0.348	-0.096	0.822	0.887
TLI	500 km	TLI1010	original	0.348	0.063	0.646	0.972
TLI	500 km	TLI1030	original	0.35	-0.147	0.828	0.875
TLI	500 km	TLI0859	original	0.354	-0.101	0.814	0.891
TLI	500 km	TLI1080	original	0.355	-0.01	0.708	0.944
TLI	500 km	TLI0994	original	0.357	-0.057	0.762	0.916
TLI	500 km	TLI1043	original	0.358	-0.09	0.814	0.896
TLI	500 km	TLI0947	original	0.362	-0.038	0.786	0.921
TLI	500 km	TLI0923	original	0.365	0.005	0.708	0.95
TLI	500 km	TLI1092	original	0.37	0.017	0.708	0.956
TLI	500 km	TLI0895	original	0.379	0.182	0.576	0.998
TLI	500 km	TLI0842	original	0.381	-0.079	0.874	0.902
TLI	500 km	TLI1025	original	0.388	-0.11	0.909	0.89
TLI	500 km	TLI0986	original	0.39	-0.115	0.9	0.889
TLI	500 km	TLI0896	original	0.394	0.135	0.65	0.992
TLI	500 km	TLI0981	original	0.395	-0.129	0.913	0.888
TLI	500 km	TLI0996	original	0.395	0.028	0.751	0.96
TLI	500 km	TLI1105	original	0.4	-0.022	0.85	0.929
TLI	500 km	TLI0381	original	0.407	-0.031	0.846	0.931
TLI	500 km	TLI1091	original	0.41	0.244	0.581	1
TLI	500 km	TLI0596	original	0.412	0.105	0.717	0.985
TLI	500 km	TLI1027	original	0.413	-0.104	0.915	0.901
TLI	500 km	TLI0636	original	0.418	0.194	0.634	0.999
TLI	500 km	TLI0120	original	0.42	0.177	0.674	0.997
TLI	500 km	TLI0673	original	0.421	0.154	0.681	0.995
TLI	500 km	TLI1020	original	0.422	0.051	0.778	0.969
TLI	500 km	TLI0847	original	0.424	-0.027	0.873	0.935
TLI	500 km	TLI1067	original	0.427	0.034	0.793	0.964
TLI	500 km	TLI0364	original	0.433	0.011	0.874	0.946

TLI	500 km	TLI0592	original	0.436	0.186	0.685	0.998
TLI	500 km	TLI1093	original	0.438	-0.012	0.881	0.942
TLI	500 km	TLI0993	original	0.442	0.003	0.874	0.948
TLI	500 km	TLI1017	original	0.445	-0.015	0.913	0.937
TLI	500 km	TLI0560	original	0.448	-0.037	0.927	0.93
TLI	500 km	TLI0962	original	0.449	0.112	0.776	0.984
TLI	500 km	TLI0790	original	0.471	0.069	0.881	0.969
TLI	500 km	TLI0936	original	0.473	0.08	0.847	0.976
TLI	500 km	TLI0977	original	0.473	0.122	0.819	0.984
TLI	500 km	TLI0617	original	0.474	0.252	0.698	1
TLI	500 km	TLI0980	original	0.481	0.122	0.835	0.985
TLI	500 km	TLI1096	original	0.482	0.013	0.956	0.95
TLI	500 km	TLI1087	original	0.493	0.081	0.914	0.97
TLI	500 km	TLI1101	original	0.507	0.173	0.834	0.993
TLI	500 km	TLI0398	original	0.511	0.02	0.982	0.956
TLI	500 km	TLI0900	original	0.518	-0.006	1.034	0.945
TLI	500 km	TLI0598	original	0.526	0.171	0.88	0.991
TLI	500 km	TLI0968	original	0.529	0.207	0.844	0.996
TLI	500 km	TLI0858	original	0.542	0.01	1.055	0.952
TLI	500 km	TLI0431	original	0.548	0.13	0.974	0.981
TLI	500 km	TLI0884	original	0.559	0.105	1.039	0.971
TLI	500 km	TLI0621	original	0.584	0.423	0.738	1
TLI	500 km	TLI0961	original	0.584	0.119	1.038	0.981
TLI	500 km	TLI1100	original	0.587	0.342	0.827	1
TLI	500 km	TLI1056	original	0.588	0.209	0.963	0.994
TLI	500 km	TLI0373	original	0.59	0.107	1.086	0.973
TLI	500 km	TLI0801	original	0.599	0.198	1.021	0.99
TLI	500 km	TLI1107	original	0.605	0.389	0.814	1
TLI	500 km	TLI1031	original	0.607	0.239	0.98	0.996
TLI	500 km	TLI0841	original	0.609	0.22	0.998	0.995
TLI	500 km	TLI0589	original	0.619	0.307	0.912	0.999
TLI	500 km	TLI0881	original	0.646	0.14	1.163	0.978
TLI	500 km	TLI0392	original	0.666	0.191	1.157	0.986
TLI	500 km	TLI0383	original	0.696	0.235	1.164	0.991
TLI	500 km	TLI1008	original	0.727	0.347	1.108	0.999
TLI	500 km	TLI1112	original	0.734	0.463	1.004	1
TLI	500 km	TLI0955	original	0.786	0.48	1.073	1
TLI	500 km	TLI0903	original	0.81	0.334	1.293	0.997
TLI	500 km	TLI0604	original	0.839	0.328	1.35	0.996

TLI	500 km	TLI0989	original	0.841	0.36	1.303	0.998
TLI	500 km	TLI1051	original	0.845	0.593	1.094	1
TLI	500 km	TLI0840	original	0.849	0.383	1.3	0.998
TLI	500 km	TLI0570	original	0.885	0.513	1.256	1
TLI	500 km	TLI1006	original	0.939	0.601	1.283	1
TLI	500 km	TLI0928	original	0.965	0.594	1.343	1
TLI	500 km	TLI1007	original	0.98	0.58	1.373	1
TLI	500 km	TLI0939	original	1.047	0.641	1.461	1
TLI	500 km	TLI1052	original	1.092	0.826	1.362	1
TLI	500 km	TLI0559	original	1.16	0.829	1.498	1
TLI	500 km	TLI0958	original	1.179	0.772	1.611	1
GBI	300 km	GB192c	original	-1.261	-1.534	-0.988	1
GBI	300 km	GB030	original	-0.948	-1.217	-0.668	1
GBI	300 km	GB321c	original	-0.872	-1.194	-0.552	1
GBI	300 km	GB167	original	-0.859	-1.143	-0.571	1
GBI	300 km	GB703drmc	original	-0.829	-1.198	-0.474	1
GBI	300 km	GB041c	original	-0.799	-1.034	-0.55	1
GBI	300 km	GB254c	original	-0.788	-1.103	-0.493	1
GBI	300 km	GB326	original	-0.778	-1.085	-0.493	1
GBI	300 km	GB039c	original	-0.708	-0.997	-0.419	1
GBI	300 km	GB110cC	original	-0.681	-0.876	-0.485	1
GBI	300 km	GB947mc	original	-0.663	-1.004	-0.334	0.999
GBI	300 km	GB126	original	-0.647	-0.934	-0.368	1
GBI	300 km	GB252c	original	-0.553	-0.913	-0.199	0.994
GBI	300 km	GB075c	original	-0.548	-0.874	-0.214	0.995
GBI	300 km	GB313	original	-0.525	-0.791	-0.25	0.999
GBI	300 km	GB590mC	original	-0.522	-0.841	-0.207	0.996
GBI	300 km	GB256	original	-0.49	-0.761	-0.226	0.998
GBI	300 km	GB257	original	-0.448	-0.745	-0.137	0.991
GBI	300 km	GB159	original	-0.437	-0.738	-0.146	0.99
GBI	300 km	GB137	original	-0.393	-0.683	-0.105	0.984
GBI	300 km	GB887drmc	original	-0.393	-0.671	-0.101	0.986
GBI	300 km	GB291c	original	-0.36	-0.699	-0.014	0.954
GBI	300 km	GB300	original	-0.359	-0.613	-0.115	0.989
GBI	300 km	GB296	original	-0.346	-0.66	-0.02	0.959
GBI	300 km	GB134	original	-0.318	-0.47	-0.161	0.998
GBI	300 km	GB591mC	original	-0.317	-0.602	-0.021	0.958
GBI	300 km	GB022c	original	-0.298	-0.644	0.031	0.921
GBI	300 km	GB150	original	-0.29	-0.569	0.002	0.949

GBI	300 km	GB038c	original	-0.287	-0.775	0.187	0.831
GBI	300 km	GB553drmcC	original	-0.278	-0.597	0.028	0.921
GBI	300 km	GB299	original	-0.276	-0.57	0.011	0.936
GBI	300 km	GB329c	original	-0.274	-0.74	0.175	0.832
GBI	300 km	GB402	original	-0.265	-0.489	-0.047	0.971
GBI	300 km	GB197C	original	-0.247	-0.657	0.14	0.838
GBI	300 km	GB111C	original	-0.236	-0.481	0.021	0.933
GBI	300 km	GB138	original	-0.227	-0.505	0.043	0.908
GBI	300 km	GB522C	original	-0.22	-0.501	0.046	0.903
GBI	300 km	GB129	original	-0.218	-0.455	0.014	0.932
GBI	300 km	GB074c	original	-0.21	-0.549	0.159	0.829
GBI	300 km	GB704drm	original	-0.207	-0.495	0.069	0.882
GBI	300 km	GB800EO	original	-0.204	-0.628	0.223	0.777
GBI	300 km	GB501drm	original	-0.198	-0.52	0.119	0.838
GBI	300 km	GB262c	original	-0.195	-0.534	0.165	0.812
GBI	300 km	GB123	original	-0.193	-0.482	0.101	0.855
GBI	300 km	GB431c	original	-0.19	-0.59	0.218	0.773
GBI	300 km	GB084c	original	-0.182	-0.461	0.093	0.856
GBI	300 km	GB204	original	-0.182	-0.49	0.13	0.824
GBI	300 km	GB625mC	original	-0.171	-0.443	0.122	0.833
GBI	300 km	GB152	original	-0.168	-0.444	0.112	0.835
GBI	300 km	GB500drm	original	-0.165	-0.439	0.116	0.83
GBI	300 km	GB403	original	-0.161	-0.444	0.139	0.812
GBI	300 km	GB082cC	original	-0.158	-0.477	0.176	0.779
GBI	300 km	GB059	original	-0.152	-0.426	0.115	0.814
GBI	300 km	GB558drm	original	-0.151	-0.353	0.039	0.894
GBI	300 km	GB335	original	-0.148	-0.413	0.113	0.818
GBI	300 km	GB325	original	-0.145	-0.375	0.095	0.841
GBI	300 km	GB951drmcC	original	-0.145	-0.417	0.124	0.805
GBI	300 km	GB081	original	-0.141	-0.377	0.087	0.837
GBI	300 km	GB949m	original	-0.138	-0.434	0.146	0.778
GBI	300 km	GB196C	original	-0.134	-0.613	0.307	0.683
GBI	300 km	GB155C	original	-0.125	-0.426	0.176	0.747
GBI	300 km	GB309c	original	-0.125	-0.388	0.136	0.779
GBI	300 km	GB133C	original	-0.112	-0.501	0.253	0.682
GBI	300 km	GB049C	original	-0.112	-0.392	0.161	0.742
GBI	300 km	GB702drmC	original	-0.11	-0.465	0.27	0.687
GBI	300 km	GB415	original	-0.109	-0.385	0.164	0.74
GBI	300 km	GB250	original	-0.106	-0.422	0.208	0.705

GBI	300 km	GB263c	original	-0.104	-0.433	0.231	0.689
GBI	300 km	GB433c	original	-0.095	-0.48	0.295	0.651
GBI	300 km	GB135	original	-0.086	-0.337	0.16	0.712
GBI	300 km	GB421c	original	-0.072	-0.419	0.268	0.632
GBI	300 km	GB107C	original	-0.067	-0.361	0.209	0.646
GBI	300 km	GB136	original	-0.066	-0.309	0.196	0.664
GBI	300 km	GB057c	original	-0.064	-0.532	0.377	0.59
GBI	300 km	GB480m	original	-0.063	-0.349	0.236	0.635
GBI	300 km	GB104	original	-0.054	-0.341	0.215	0.623
GBI	300 km	GB069	original	-0.053	-0.293	0.188	0.637
GBI	300 km	GB099	original	-0.05	-0.284	0.183	0.63
GBI	300 km	GB606m	original	-0.047	-0.323	0.234	0.607
GBI	300 km	GB301	original	-0.047	-0.295	0.193	0.619
GBI	300 km	GB113	original	-0.044	-0.333	0.238	0.598
GBI	300 km	GB023c	original	-0.043	-0.372	0.276	0.585
GBI	300 km	GB302c	original	-0.04	-0.37	0.305	0.577
GBI	300 km	GB158	original	-0.037	-0.302	0.217	0.59
GBI	300 km	GB422c	original	-0.033	-0.359	0.296	0.565
GBI	300 km	GB139	original	-0.032	-0.302	0.229	0.576
GBI	300 km	GB550m	original	-0.03	-0.292	0.24	0.569
GBI	300 km	GB567drm	original	-0.029	-0.291	0.241	0.571
GBI	300 km	GB286c	original	-0.028	-0.384	0.328	0.548
GBI	300 km	GB253c	original	-0.02	-0.377	0.335	0.531
GBI	300 km	GB140	original	-0.016	-0.319	0.292	0.53
GBI	300 km	GB260	original	-0.016	-0.196	0.164	0.553
GBI	300 km	GB028	original	-0.016	-0.269	0.243	0.538
GBI	300 km	GB950m	original	-0.006	-0.311	0.289	0.514
GBI	300 km	GB298	original	-0.005	-0.2	0.181	0.513
GBI	300 km	GB035	original	-0.004	-0.3	0.269	0.508
GBI	300 km	GB068	original	-0.003	-0.295	0.293	0.505
GBI	300 km	GB297	original	0	-0.261	0.272	0.503
GBI	300 km	GB432c	original	0	-0.329	0.327	0.503
GBI	300 km	GB334	original	0.009	-0.3	0.312	0.517
GBI	300 km	GB118	original	0.019	-0.277	0.321	0.541
GBI	300 km	GB065e	original	0.037	-0.25	0.327	0.584
GBI	300 km	GB108	original	0.051	-0.231	0.342	0.613
GBI	300 km	GB327	original	0.052	-0.172	0.268	0.647
GBI	300 km	GB095C	original	0.057	-0.143	0.265	0.676
GBI	300 km	GB622drmc	original	0.057	-0.24	0.343	0.622

GBI	300 km	GB127	original	0.059	-0.218	0.347	0.63
GBI	300 km	GB556EO	original	0.065	-0.477	0.61	0.576
GBI	300 km	GB147c	original	0.072	-0.235	0.377	0.648
GBI	300 km	GB322c	original	0.078	-0.373	0.525	0.611
GBI	300 km	GB620drmc	original	0.086	-0.189	0.361	0.693
GBI	300 km	GB054c	original	0.086	-0.234	0.413	0.666
GBI	300 km	GB265	original	0.088	-0.186	0.368	0.695
GBI	300 km	GB569drmcC	original	0.091	-0.293	0.498	0.642
GBI	300 km	GB048	original	0.106	-0.153	0.362	0.744
GBI	300 km	GB103	original	0.113	-0.16	0.401	0.744
GBI	300 km	GB331C	original	0.114	-0.09	0.312	0.819
GBI	300 km	GB549drmc	original	0.116	-0.119	0.329	0.797
GBI	300 km	GB149C	original	0.124	-0.118	0.363	0.798
GBI	300 km	GB323c	original	0.144	-0.22	0.504	0.737
GBI	300 km	GB624drmc	original	0.154	-0.163	0.472	0.783
GBI	300 km	GB304c	original	0.155	-0.209	0.521	0.754
GBI	300 km	GB559drm	original	0.158	-0.03	0.347	0.911
GBI	300 km	GB273	original	0.169	-0.096	0.437	0.844
GBI	300 km	GB026	original	0.178	-0.046	0.405	0.895
GBI	300 km	GB430c	original	0.18	-0.168	0.521	0.8
GBI	300 km	GB623drmc	original	0.182	-0.116	0.48	0.837
GBI	300 km	GB850drm	original	0.183	-0.017	0.371	0.933
GBI	300 km	GB146	original	0.187	-0.102	0.481	0.85
GBI	300 km	GB046C	original	0.19	-0.05	0.432	0.896
GBI	300 km	GB852drmC	original	0.19	-0.11	0.48	0.85
GBI	300 km	GB264c	original	0.203	-0.061	0.487	0.883
GBI	300 km	GB995F	original	0.213	0.016	0.416	0.954
GBI	300 km	GB701drm	original	0.216	-0.046	0.485	0.904
GBI	300 km	GB900EO	original	0.216	0.033	0.398	0.969
GBI	300 km	GB330c	original	0.217	-0.253	0.692	0.77
GBI	300 km	GB888drmcC	original	0.222	-0.084	0.528	0.877
GBI	300 km	GB098C	original	0.227	-0.015	0.463	0.934
GBI	300 km	GB621drmc	original	0.232	-0.056	0.504	0.907
GBI	300 km	GB332EON	original	0.234	0.003	0.442	0.955
GBI	300 km	GB132	original	0.241	-0.041	0.536	0.909
GBI	300 km	GB285cc	original	0.246	-0.336	0.822	0.753
GBI	300 km	GB853drm	original	0.248	-0.047	0.534	0.914
GBI	300 km	GB052c	original	0.248	-0.061	0.555	0.899
GBI	300 km	GB955drmc	original	0.252	0.056	0.462	0.973

GBI	300 km	GB083c	original	0.261	0.027	0.491	0.963
GBI	300 km	GB401	original	0.266	-0.014	0.551	0.933
GBI	300 km	GB551mC	original	0.281	0	0.564	0.944
GBI	300 km	GB777drm	original	0.282	-0.002	0.582	0.938
GBI	300 km	GB037	original	0.3	0.144	0.463	0.995
GBI	300 km	GB109C	original	0.308	-0.008	0.635	0.938
GBI	300 km	GB117	original	0.326	0.092	0.558	0.986
GBI	300 km	GB027	original	0.337	0.067	0.618	0.974
GBI	300 km	GB954drmcC	original	0.339	0.05	0.62	0.971
GBI	300 km	GB131	original	0.341	0.049	0.629	0.97
GBI	300 km	GB105	original	0.347	0.081	0.601	0.982
GBI	300 km	GB051c	original	0.359	0.009	0.7	0.952
GBI	300 km	GB555m	original	0.372	0.105	0.643	0.986
GBI	300 km	GB556m	original	0.398	0.164	0.635	0.995
GBI	300 km	GB563m	original	0.405	0.193	0.627	0.998
GBI	300 km	GB151	original	0.426	0.164	0.708	0.993
GBI	300 km	GB953drmcC	original	0.434	-0.155	0.991	0.888
GBI	300 km	GB408	original	0.436	0.144	0.719	0.992
GBI	300 km	GB116	original	0.44	0.138	0.731	0.992
GBI	300 km	GB568drm	original	0.464	0.207	0.718	0.998
GBI	300 km	GB251m	original	0.467	0.185	0.753	0.996
GBI	300 km	GB058C	original	0.473	0.123	0.827	0.983
GBI	300 km	GB156	original	0.479	0.259	0.698	0.999
GBI	300 km	GB410	original	0.493	0.26	0.731	0.999
GBI	300 km	GB020c	original	0.498	0.238	0.759	0.999
GBI	300 km	GB510m	original	0.51	0.21	0.808	0.997
GBI	300 km	GB324	original	0.535	0.323	0.758	1
GBI	300 km	GB203e	original	0.582	0.319	0.848	0.999
GBI	300 km	GB031	original	0.608	0.365	0.843	1
GBI	300 km	GB560drmcC	original	0.618	0.274	0.949	0.998
GBI	300 km	GB130e	original	0.628	0.323	0.938	0.999
GBI	300 km	GB024e	original	0.635	0.246	1.003	0.996
GBI	300 km	GB800m	original	0.639	0.343	0.934	1
GBI	300 km	GB177	original	0.643	0.403	0.886	1
GBI	300 km	GB990drmcC	original	0.658	0.379	0.935	1
GBI	300 km	GB198c	original	0.697	0.364	1.024	1
GBI	300 km	GB047	original	0.722	0.492	0.971	1
GBI	300 km	GB053c	original	0.722	0.455	0.994	1
GBI	300 km	GB595m	original	0.727	0.474	0.97	1

GBI	300 km	GB557drm	original	0.732	0.523	0.949	1
GBI	300 km	GB122	original	0.735	0.49	0.984	1
GBI	300 km	GB952drmcC	original	0.744	0.467	1	1
GBI	300 km	GB561drmc	original	0.78	0.535	1.034	1
GBI	300 km	GB096C	original	0.8	0.556	1.03	1
GBI	300 km	GB036	original	0.811	0.547	1.093	1
GBI	300 km	GB945m	original	0.856	0.601	1.123	1
GBI	300 km	GB021c	original	0.858	0.602	1.113	1
GBI	300 km	GB552m	original	0.924	0.652	1.184	1
GBI	300 km	GB409	original	0.985	0.674	1.292	1
GBI	300 km	GB991drmcC	original	1.006	0.729	1.284	1
GBI	300 km	GB124	original	1.11	0.848	1.356	1
GBI	500 km	GB192c	original	-1.182	-1.475	-0.875	1
GBI	500 km	GB030	original	-0.983	-1.253	-0.715	1
GBI	500 km	GB703drmc	original	-0.897	-1.238	-0.554	1
GBI	500 km	GB326	original	-0.888	-1.176	-0.611	1
GBI	500 km	GB167	original	-0.881	-1.131	-0.625	1
GBI	500 km	GB321c	original	-0.818	-1.122	-0.508	1
GBI	500 km	GB947mc	original	-0.796	-1.122	-0.469	1
GBI	500 km	GB041c	original	-0.746	-0.961	-0.535	1
GBI	500 km	GB252c	original	-0.725	-1.066	-0.387	1
GBI	500 km	GB254c	original	-0.691	-1.012	-0.367	1
GBI	500 km	GB291c	original	-0.666	-0.997	-0.335	0.999
GBI	500 km	GB126	original	-0.537	-0.821	-0.261	0.999
GBI	500 km	GB039c	original	-0.537	-0.818	-0.242	0.998
GBI	500 km	GB313	original	-0.537	-0.801	-0.28	0.999
GBI	500 km	GB110cC	original	-0.518	-0.734	-0.293	1
GBI	500 km	GB590mC	original	-0.486	-0.79	-0.173	0.994
GBI	500 km	GB257	original	-0.474	-0.76	-0.175	0.994
GBI	500 km	GB075c	original	-0.426	-0.729	-0.114	0.986
GBI	500 km	GB159	original	-0.425	-0.717	-0.125	0.99
GBI	500 km	GB591mC	original	-0.379	-0.645	-0.118	0.989
GBI	500 km	GB329c	original	-0.379	-0.846	0.078	0.905
GBI	500 km	GB256	original	-0.374	-0.644	-0.106	0.986
GBI	500 km	GB887drmc	original	-0.353	-0.589	-0.112	0.99
GBI	500 km	GB300	original	-0.344	-0.62	-0.074	0.977
GBI	500 km	GB150	original	-0.341	-0.611	-0.061	0.975
GBI	500 km	GB415	original	-0.322	-0.574	-0.077	0.98
GBI	500 km	GB296	original	-0.29	-0.585	0.014	0.938

GBI	500 km	GB038c	original	-0.277	-0.707	0.167	0.844
GBI	500 km	GB431c	original	-0.261	-0.607	0.081	0.887
GBI	500 km	GB155C	original	-0.259	-0.533	0.001	0.94
GBI	500 km	GB134	original	-0.246	-0.409	-0.08	0.99
GBI	500 km	GB704drm	original	-0.231	-0.509	0.048	0.908
GBI	500 km	GB137	original	-0.228	-0.505	0.057	0.901
GBI	500 km	GB500drm	original	-0.222	-0.491	0.051	0.906
GBI	500 km	GB553drmcC	original	-0.216	-0.523	0.088	0.869
GBI	500 km	GB402	original	-0.213	-0.448	0.029	0.923
GBI	500 km	GB262c	original	-0.209	-0.532	0.09	0.857
GBI	500 km	GB325	original	-0.207	-0.391	-0.007	0.958
GBI	500 km	GB123	original	-0.205	-0.489	0.081	0.875
GBI	500 km	GB022c	original	-0.202	-0.532	0.125	0.839
GBI	500 km	GB335	original	-0.2	-0.48	0.065	0.88
GBI	500 km	GB129	original	-0.199	-0.426	0.019	0.924
GBI	500 km	GB138	original	-0.198	-0.473	0.09	0.87
GBI	500 km	GB197C	original	-0.198	-0.582	0.17	0.801
GBI	500 km	GB501drm	original	-0.184	-0.468	0.118	0.842
GBI	500 km	GB302c	original	-0.172	-0.479	0.148	0.811
GBI	500 km	GB074c	original	-0.168	-0.523	0.189	0.772
GBI	500 km	GB949m	original	-0.164	-0.44	0.116	0.827
GBI	500 km	GB309c	original	-0.159	-0.416	0.099	0.84
GBI	500 km	GB299	original	-0.153	-0.438	0.126	0.808
GBI	500 km	GB800EO	original	-0.141	-0.514	0.236	0.724
GBI	500 km	GB111C	original	-0.135	-0.377	0.109	0.814
GBI	500 km	GB297	original	-0.132	-0.399	0.13	0.786
GBI	500 km	GB049C	original	-0.131	-0.373	0.11	0.807
GBI	500 km	GB522C	original	-0.126	-0.373	0.126	0.79
GBI	500 km	GB558drm	original	-0.12	-0.327	0.083	0.829
GBI	500 km	GB433c	original	-0.115	-0.441	0.222	0.711
GBI	500 km	GB204	original	-0.11	-0.419	0.208	0.715
GBI	500 km	GB057c	original	-0.104	-0.489	0.284	0.663
GBI	500 km	GB152	original	-0.1	-0.383	0.161	0.723
GBI	500 km	GB081	original	-0.096	-0.317	0.136	0.752
GBI	500 km	GB158	original	-0.095	-0.336	0.144	0.737
GBI	500 km	GB301	original	-0.094	-0.342	0.159	0.728
GBI	500 km	GB606m	original	-0.09	-0.317	0.134	0.739
GBI	500 km	GB136	original	-0.079	-0.335	0.174	0.691
GBI	500 km	GB035	original	-0.077	-0.361	0.218	0.667

GBI	500 km	GB432c	original	-0.076	-0.385	0.222	0.654
GBI	500 km	GB139	original	-0.064	-0.305	0.182	0.662
GBI	500 km	GB951drmcC	original	-0.06	-0.312	0.198	0.651
GBI	500 km	GB147c	original	-0.054	-0.333	0.225	0.621
GBI	500 km	GB069	original	-0.049	-0.272	0.181	0.634
GBI	500 km	GB135	original	-0.048	-0.277	0.168	0.636
GBI	500 km	GB082cC	original	-0.046	-0.352	0.242	0.598
GBI	500 km	GB054c	original	-0.043	-0.366	0.283	0.584
GBI	500 km	GB028	original	-0.041	-0.29	0.205	0.607
GBI	500 km	GB702drmC	original	-0.039	-0.375	0.278	0.575
GBI	500 km	GB104	original	-0.036	-0.324	0.266	0.576
GBI	500 km	GB059	original	-0.033	-0.291	0.226	0.582
GBI	500 km	GB480m	original	-0.03	-0.325	0.26	0.565
GBI	500 km	GB113	original	-0.027	-0.303	0.231	0.563
GBI	500 km	GB084c	original	-0.021	-0.266	0.236	0.555
GBI	500 km	GB567drm	original	-0.018	-0.263	0.238	0.543
GBI	500 km	GB286c	original	-0.016	-0.376	0.358	0.528
GBI	500 km	GB421c	original	-0.016	-0.374	0.343	0.529
GBI	500 km	GB625mC	original	-0.002	-0.267	0.277	0.503
GBI	500 km	GB298	original	0.007	-0.197	0.214	0.523
GBI	500 km	GB107C	original	0.013	-0.255	0.292	0.53
GBI	500 km	GB422c	original	0.023	-0.318	0.388	0.539
GBI	500 km	GB403	original	0.023	-0.235	0.294	0.556
GBI	500 km	GB327	original	0.024	-0.205	0.261	0.57
GBI	500 km	GB068	original	0.028	-0.254	0.31	0.567
GBI	500 km	GB099	original	0.038	-0.191	0.275	0.604
GBI	500 km	GB048	original	0.043	-0.201	0.276	0.615
GBI	500 km	GB250	original	0.047	-0.256	0.357	0.596
GBI	500 km	GB052c	original	0.049	-0.231	0.336	0.609
GBI	500 km	GB133C	original	0.051	-0.293	0.408	0.593
GBI	500 km	GB260	original	0.052	-0.137	0.248	0.673
GBI	500 km	GB430c	original	0.054	-0.248	0.367	0.612
GBI	500 km	GB196C	original	0.058	-0.341	0.484	0.591
GBI	500 km	GB108	original	0.067	-0.218	0.354	0.646
GBI	500 km	GB263c	original	0.068	-0.233	0.377	0.642
GBI	500 km	GB253c	original	0.078	-0.247	0.415	0.649
GBI	500 km	GB950m	original	0.081	-0.21	0.384	0.67
GBI	500 km	GB140	original	0.082	-0.221	0.383	0.673
GBI	500 km	GB323c	original	0.087	-0.267	0.433	0.655

GBI	500 km	GB265	original	0.1	-0.165	0.365	0.729
GBI	500 km	GB023c	original	0.102	-0.229	0.417	0.695
GBI	500 km	GB118	original	0.112	-0.202	0.417	0.718
GBI	500 km	GB556EO	original	0.112	-0.347	0.571	0.651
GBI	500 km	GB550m	original	0.115	-0.152	0.382	0.758
GBI	500 km	GB334	original	0.119	-0.184	0.412	0.739
GBI	500 km	GB095C	original	0.124	-0.074	0.329	0.84
GBI	500 km	GB622drmc	original	0.133	-0.143	0.435	0.77
GBI	500 km	GB850drm	original	0.139	-0.032	0.313	0.903
GBI	500 km	GB046C	original	0.139	-0.071	0.363	0.85
GBI	500 km	GB065e	original	0.142	-0.141	0.423	0.792
GBI	500 km	GB322c	original	0.144	-0.305	0.592	0.696
GBI	500 km	GB549drmc	original	0.171	-0.067	0.418	0.87
GBI	500 km	GB852drmC	original	0.174	-0.107	0.448	0.843
GBI	500 km	GB103	original	0.178	-0.1	0.454	0.844
GBI	500 km	GB285cc	original	0.18	-0.334	0.677	0.715
GBI	500 km	GB332EON	original	0.198	0.003	0.39	0.948
GBI	500 km	GB569drmcC	original	0.199	-0.201	0.582	0.793
GBI	500 km	GB559drm	original	0.202	0.027	0.368	0.968
GBI	500 km	GB146	original	0.203	-0.079	0.482	0.875
GBI	500 km	GB330c	original	0.203	-0.267	0.659	0.76
GBI	500 km	GB620drmc	original	0.205	-0.072	0.485	0.88
GBI	500 km	GB026	original	0.21	0.011	0.416	0.952
GBI	500 km	GB623drmc	original	0.211	-0.091	0.498	0.875
GBI	500 km	GB995F	original	0.219	0.034	0.398	0.971
GBI	500 km	GB777drm	original	0.224	-0.073	0.505	0.893
GBI	500 km	GB955drmc	original	0.246	0.052	0.446	0.974
GBI	500 km	GB127	original	0.247	-0.01	0.498	0.939
GBI	500 km	GB304c	original	0.247	-0.078	0.565	0.89
GBI	500 km	GB900EO	original	0.259	0.081	0.444	0.986
GBI	500 km	GB701drm	original	0.288	0.009	0.547	0.956
GBI	500 km	GB098C	original	0.288	0.07	0.508	0.981
GBI	500 km	GB331C	original	0.291	0.075	0.514	0.981
GBI	500 km	GB132	original	0.302	0.02	0.577	0.958
GBI	500 km	GB551mC	original	0.31	0.058	0.571	0.972
GBI	500 km	GB954drmcC	original	0.317	0.048	0.566	0.975
GBI	500 km	GB037	original	0.327	0.145	0.513	0.996
GBI	500 km	GB510m	original	0.34	0.06	0.616	0.974
GBI	500 km	GB264c	original	0.354	0.073	0.635	0.978

GBI	500 km	GB563m	original	0.354	0.136	0.565	0.995
GBI	500 km	GB624drmc	original	0.36	0.073	0.659	0.975
GBI	500 km	GB105	original	0.363	0.094	0.624	0.984
GBI	500 km	GB109C	original	0.374	0.061	0.674	0.975
GBI	500 km	GB273	original	0.375	0.111	0.647	0.986
GBI	500 km	GB853drm	original	0.381	0.102	0.661	0.984
GBI	500 km	GB058C	original	0.382	0.045	0.71	0.967
GBI	500 km	GB621drmc	original	0.382	0.101	0.647	0.986
GBI	500 km	GB401	original	0.388	0.096	0.684	0.982
GBI	500 km	GB888drmcC	original	0.411	0.125	0.707	0.988
GBI	500 km	GB149C	original	0.418	0.122	0.706	0.988
GBI	500 km	GB083c	original	0.421	0.171	0.677	0.995
GBI	500 km	GB027	original	0.422	0.161	0.674	0.995
GBI	500 km	GB117	original	0.439	0.209	0.67	0.998
GBI	500 km	GB051c	original	0.44	0.132	0.752	0.989
GBI	500 km	GB953drmcC	original	0.447	-0.05	0.961	0.92
GBI	500 km	GB156	original	0.452	0.188	0.718	0.996
GBI	500 km	GB556m	original	0.458	0.226	0.695	0.998
GBI	500 km	GB151	original	0.471	0.226	0.706	0.998
GBI	500 km	GB555m	original	0.471	0.204	0.737	0.997
GBI	500 km	GB116	original	0.471	0.217	0.73	0.997
GBI	500 km	GB031	original	0.479	0.238	0.722	0.999
GBI	500 km	GB568drm	original	0.481	0.231	0.739	0.998
GBI	500 km	GB020c	original	0.505	0.242	0.759	0.999
GBI	500 km	GB131	original	0.506	0.22	0.777	0.997
GBI	500 km	GB410	original	0.517	0.284	0.741	1
GBI	500 km	GB408	original	0.541	0.268	0.823	0.999
GBI	500 km	GB251m	original	0.543	0.254	0.821	0.998
GBI	500 km	GB324	original	0.565	0.327	0.795	1
GBI	500 km	GB122	original	0.572	0.314	0.838	1
GBI	500 km	GB198c	original	0.585	0.206	0.959	0.994
GBI	500 km	GB595m	original	0.607	0.357	0.855	1
GBI	500 km	GB952drmcC	original	0.638	0.346	0.937	1
GBI	500 km	GB130e	original	0.639	0.344	0.93	1
GBI	500 km	GB024e	original	0.67	0.333	1.005	0.999
GBI	500 km	GB203e	original	0.675	0.413	0.929	1
GBI	500 km	GB557drm	original	0.703	0.493	0.922	1
GBI	500 km	GB053c	original	0.72	0.42	1.009	1
GBI	500 km	GB047	original	0.724	0.485	0.966	1

GBI	500 km	GB990drmcC	original	0.725	0.459	0.993	1
GBI	500 km	GB177	original	0.73	0.478	0.969	1
GBI	500 km	GB560drmcC	original	0.739	0.386	1.099	0.999
GBI	500 km	GB800m	original	0.746	0.483	1.014	1
GBI	500 km	GB021c	original	0.767	0.519	1.02	1
GBI	500 km	GB036	original	0.835	0.572	1.094	1
GBI	500 km	GB561drmc	original	0.873	0.613	1.131	1
GBI	500 km	GB945m	original	0.88	0.636	1.119	1
GBI	500 km	GB096C	original	0.886	0.642	1.11	1
GBI	500 km	GB552m	original	0.892	0.631	1.157	1
GBI	500 km	GB409	original	0.997	0.685	1.282	1
GBI	500 km	GB991drmcC	original	1.012	0.737	1.292	1
GBI	500 km	GB124	original	1.23	0.982	1.485	1
TLI	300 km	TLI1055	jittered	-1.479	-1.705	-1.247	1
TLI	300 km	TLI0855	jittered	-1.07	-1.427	-0.711	1
TLI	300 km	TLI1066	jittered	-0.914	-1.133	-0.685	1
TLI	300 km	TLI0612	jittered	-0.76	-0.962	-0.547	1
TLI	300 km	TLI1016	jittered	-0.735	-1.03	-0.441	1
TLI	300 km	TLI1069	jittered	-0.696	-0.947	-0.46	1
TLI	300 km	TLI0102	jittered	-0.688	-1.099	-0.263	0.996
TLI	300 km	TLI1039	jittered	-0.677	-0.927	-0.428	1
TLI	300 km	TLI0578	jittered	-0.67	-0.929	-0.402	1
TLI	300 km	TLI1060	jittered	-0.645	-0.834	-0.454	1
TLI	300 km	TLI0615	jittered	-0.638	-0.784	-0.489	1
TLI	300 km	TLI1063	jittered	-0.63	-1.085	-0.193	0.988
TLI	300 km	TLI0670	jittered	-0.624	-1.018	-0.23	0.994
TLI	300 km	TLI1036	jittered	-0.589	-0.962	-0.195	0.993
TLI	300 km	TLI1103	jittered	-0.554	-0.731	-0.375	1
TLI	300 km	TLI0966	jittered	-0.55	-0.838	-0.258	0.999
TLI	300 km	TLI0635	jittered	-0.542	-0.698	-0.386	1
TLI	300 km	TLI1085	jittered	-0.534	-0.875	-0.175	0.993
TLI	300 km	TLI1049	jittered	-0.499	-0.662	-0.337	1
TLI	300 km	TLI1110	jittered	-0.497	-0.778	-0.208	0.997
TLI	300 km	TLI1083	jittered	-0.489	-1.046	0.073	0.919
TLI	300 km	TLI0856	jittered	-0.479	-0.911	-0.062	0.965
TLI	300 km	TLI0893	jittered	-0.464	-0.712	-0.204	0.998
TLI	300 km	TLI0630	jittered	-0.449	-0.591	-0.315	1
TLI	300 km	TLI0453	jittered	-0.448	-0.609	-0.283	1
TLI	300 km	TLI0979	jittered	-0.431	-0.585	-0.272	1

TLI	300 km	TLI0861	jittered	-0.411	-0.707	-0.128	0.988
TLI	300 km	TLI0081	jittered	-0.395	-0.936	0.166	0.875
TLI	300 km	TLI0957	jittered	-0.393	-0.74	-0.04	0.963
TLI	300 km	TLI1011	jittered	-0.393	-0.748	-0.047	0.965
TLI	300 km	TLI0631	jittered	-0.381	-0.561	-0.201	1
TLI	300 km	TLI0908	jittered	-0.38	-0.854	0.09	0.9
TLI	300 km	TLI0976	jittered	-0.369	-0.719	-0.021	0.954
TLI	300 km	TLI1012	jittered	-0.367	-0.84	0.099	0.896
TLI	300 km	TLI1099	jittered	-0.364	-0.634	-0.107	0.986
TLI	300 km	TLI0956	jittered	-0.362	-0.709	-0.003	0.949
TLI	300 km	TLI0518	jittered	-0.356	-0.699	0	0.948
TLI	300 km	TLI0954	jittered	-0.331	-0.706	0.028	0.926
TLI	300 km	TLI1104	jittered	-0.317	-0.497	-0.134	0.997
TLI	300 km	TLI1076	jittered	-0.312	-0.622	-0.007	0.947
TLI	300 km	TLI0371	jittered	-0.305	-0.722	0.123	0.875
TLI	300 km	TLI1029	jittered	-0.301	-0.79	0.173	0.842
TLI	300 km	TLI1111	jittered	-0.301	-0.582	-0.027	0.957
TLI	300 km	TLI0608	jittered	-0.3	-0.44	-0.156	1
TLI	300 km	TLI1057	jittered	-0.294	-0.647	0.077	0.903
TLI	300 km	TLI0804	jittered	-0.291	-0.59	0.007	0.941
TLI	300 km	TLI0844	jittered	-0.288	-0.734	0.185	0.844
TLI	300 km	TLI1062	jittered	-0.287	-0.439	-0.14	0.999
TLI	300 km	TLI0044	jittered	-0.281	-0.56	-0.002	0.946
TLI	300 km	TLI0107	jittered	-0.281	-0.63	0.101	0.891
TLI	300 km	TLI1065	jittered	-0.275	-0.461	-0.094	0.992
TLI	300 km	TLI0512	jittered	-0.271	-0.66	0.123	0.863
TLI	300 km	TLI0919	jittered	-0.269	-0.846	0.302	0.776
TLI	300 km	TLI1075	jittered	-0.261	-0.447	-0.085	0.989
TLI	300 km	TLI1028	jittered	-0.256	-0.545	0.012	0.929
TLI	300 km	TLI1023	jittered	-0.252	-0.772	0.312	0.773
TLI	300 km	TLI0886	jittered	-0.251	-0.786	0.281	0.775
TLI	300 km	TLI0984	jittered	-0.251	-0.696	0.176	0.82
TLI	300 km	TLI0874	jittered	-0.251	-0.638	0.137	0.848
TLI	300 km	TLI1094	jittered	-0.241	-0.703	0.216	0.797
TLI	300 km	TLI0975	jittered	-0.236	-0.731	0.236	0.781
TLI	300 km	TLI0580	jittered	-0.232	-0.646	0.153	0.823
TLI	300 km	TLI0933	jittered	-0.229	-0.598	0.156	0.835
TLI	300 km	TLI0140	jittered	-0.227	-0.561	0.104	0.862
TLI	300 km	TLI0891	jittered	-0.22	-0.443	-0.003	0.946

TLI	300 km	TLI0096	jittered	-0.22	-0.584	0.128	0.838
TLI	300 km	TLI1072	jittered	-0.217	-0.573	0.164	0.826
TLI	300 km	TLI0035	jittered	-0.203	-0.385	-0.02	0.962
TLI	300 km	TLI0890	jittered	-0.203	-0.457	0.045	0.902
TLI	300 km	TLI1117	jittered	-0.199	-0.696	0.31	0.735
TLI	300 km	TLI1079	jittered	-0.192	-0.586	0.219	0.777
TLI	300 km	TLI0839	jittered	-0.188	-0.493	0.116	0.84
TLI	300 km	TLI0674	jittered	-0.186	-0.403	0.038	0.912
TLI	300 km	TLI1089	jittered	-0.18	-0.582	0.218	0.762
TLI	300 km	TLI0912	jittered	-0.176	-0.722	0.354	0.699
TLI	300 km	TLI1050	jittered	-0.172	-0.337	-0.005	0.951
TLI	300 km	TLI0964	jittered	-0.161	-0.45	0.118	0.818
TLI	300 km	TLI0519	jittered	-0.158	-0.581	0.267	0.721
TLI	300 km	TLI0374	jittered	-0.158	-0.556	0.252	0.732
TLI	300 km	TLI0388	jittered	-0.157	-0.641	0.303	0.704
TLI	300 km	TLI1116	jittered	-0.157	-0.483	0.153	0.786
TLI	300 km	TLI0164	jittered	-0.156	-0.55	0.247	0.736
TLI	300 km	TLI0400	jittered	-0.151	-0.583	0.257	0.719
TLI	300 km	TLI0930	jittered	-0.151	-0.295	-0.001	0.95
TLI	300 km	TLI1040	jittered	-0.148	-0.715	0.402	0.665
TLI	300 km	TLI0973	jittered	-0.148	-0.547	0.271	0.718
TLI	300 km	TLI0915	jittered	-0.147	-0.426	0.134	0.802
TLI	300 km	TLI1090	jittered	-0.144	-0.322	0.031	0.904
TLI	300 km	TLI0867	jittered	-0.14	-0.367	0.072	0.846
TLI	300 km	TLI0902	jittered	-0.139	-0.566	0.306	0.694
TLI	300 km	TLI1098	jittered	-0.138	-0.352	0.082	0.846
TLI	300 km	TLI0792	jittered	-0.138	-0.534	0.266	0.71
TLI	300 km	TLI0910	jittered	-0.137	-0.636	0.37	0.668
TLI	300 km	TLI0943	jittered	-0.127	-0.575	0.333	0.671
TLI	300 km	TLI1009	jittered	-0.121	-0.588	0.323	0.666
TLI	300 km	TLI0440	jittered	-0.113	-0.673	0.456	0.626
TLI	300 km	TLI1041	jittered	-0.107	-0.655	0.436	0.622
TLI	300 km	TLI1014	jittered	-0.107	-0.407	0.192	0.716
TLI	300 km	TLI0880	jittered	-0.104	-0.616	0.392	0.629
TLI	300 km	TLI0892	jittered	-0.102	-0.347	0.161	0.74
TLI	300 km	TLI0848	jittered	-0.101	-0.248	0.041	0.869
TLI	300 km	TLI0654	jittered	-0.1	-0.44	0.242	0.681
TLI	300 km	TLI1106	jittered	-0.096	-0.332	0.142	0.742
TLI	300 km	TLI0629	jittered	-0.093	-0.206	0.017	0.909

TLI	300 km	TLI0999	jittered	-0.091	-0.484	0.311	0.645
TLI	300 km	TLI0879	jittered	-0.088	-0.64	0.446	0.605
TLI	300 km	TLI1042	jittered	-0.085	-0.616	0.436	0.602
TLI	300 km	TLI0597	jittered	-0.082	-0.692	0.499	0.588
TLI	300 km	TLI1003	jittered	-0.081	-0.585	0.427	0.601
TLI	300 km	TLI0771	jittered	-0.08	-0.543	0.393	0.607
TLI	300 km	TLI1088	jittered	-0.079	-0.307	0.14	0.712
TLI	300 km	TLI0376	jittered	-0.078	-0.519	0.347	0.611
TLI	300 km	TLI0965	jittered	-0.078	-0.328	0.177	0.69
TLI	300 km	TLI0987	jittered	-0.073	-0.491	0.343	0.61
TLI	300 km	TLI0960	jittered	-0.072	-0.348	0.195	0.663
TLI	300 km	TLI0514	jittered	-0.068	-0.473	0.34	0.605
TLI	300 km	TLI0552	jittered	-0.068	-0.337	0.219	0.651
TLI	300 km	TLI1019	jittered	-0.067	-0.483	0.369	0.599
TLI	300 km	TLI0849	jittered	-0.064	-0.395	0.277	0.619
TLI	300 km	TLI0916	jittered	-0.059	-0.6	0.464	0.57
TLI	300 km	TLI0974	jittered	-0.058	-0.598	0.468	0.569
TLI	300 km	TLI0613	jittered	-0.05	-0.222	0.128	0.672
TLI	300 km	TLI0931	jittered	-0.049	-0.562	0.475	0.558
TLI	300 km	TLI0970	jittered	-0.047	-0.558	0.464	0.561
TLI	300 km	TLI0906	jittered	-0.044	-0.398	0.299	0.579
TLI	300 km	TLI0894	jittered	-0.044	-0.216	0.12	0.663
TLI	300 km	TLI0920	jittered	-0.041	-0.58	0.521	0.547
TLI	300 km	TLI0875	jittered	-0.038	-0.446	0.401	0.557
TLI	300 km	TLI0624	jittered	-0.035	-0.215	0.136	0.625
TLI	300 km	TLI0846	jittered	-0.031	-0.231	0.167	0.597
TLI	300 km	TLI0850	jittered	-0.029	-0.548	0.464	0.538
TLI	300 km	TLI0909	jittered	-0.028	-0.669	0.621	0.528
TLI	300 km	TLI0668	jittered	-0.027	-0.234	0.184	0.586
TLI	300 km	TLI1022	jittered	-0.025	-0.373	0.338	0.544
TLI	300 km	TLI0945	jittered	-0.02	-0.44	0.424	0.53
TLI	300 km	TLI0967	jittered	-0.018	-0.339	0.314	0.534
TLI	300 km	TLI0937	jittered	-0.015	-0.386	0.36	0.526
TLI	300 km	TLI0853	jittered	-0.015	-0.435	0.42	0.522
TLI	300 km	TLI0515	jittered	-0.014	-0.407	0.389	0.523
TLI	300 km	TLI1015	jittered	-0.009	-0.147	0.12	0.542
TLI	300 km	TLI0905	jittered	-0.009	-0.361	0.324	0.516
TLI	300 km	TLI0887	jittered	-0.009	-0.52	0.518	0.512
TLI	300 km	TLI0370	jittered	-0.007	-0.438	0.39	0.512

TLI	300 km	TLI0005	jittered	-0.004	-0.335	0.329	0.507
TLI	300 km	TLI1074	jittered	-0.003	-0.237	0.23	0.509
TLI	300 km	TLI0640	jittered	-0.001	-0.17	0.167	0.505
TLI	300 km	TLI1082	jittered	0.002	-0.369	0.388	0.504
TLI	300 km	TLI1113	jittered	0.003	-0.169	0.178	0.515
TLI	300 km	TLI1000	jittered	0.011	-0.379	0.413	0.519
TLI	300 km	TLI0878	jittered	0.014	-0.424	0.439	0.519
TLI	300 km	TLI1047	jittered	0.015	-0.38	0.418	0.524
TLI	300 km	TLI0899	jittered	0.017	-0.477	0.536	0.521
TLI	300 km	TLI0901	jittered	0.022	-0.463	0.506	0.528
TLI	300 km	TLI0738	jittered	0.023	-0.087	0.134	0.631
TLI	300 km	TLI0672	jittered	0.026	-0.249	0.314	0.558
TLI	300 km	TLI0953	jittered	0.032	-0.404	0.454	0.547
TLI	300 km	TLI0430	jittered	0.043	-0.427	0.529	0.559
TLI	300 km	TLI0885	jittered	0.043	-0.502	0.571	0.549
TLI	300 km	TLI0560	jittered	0.043	-0.418	0.475	0.558
TLI	300 km	TLI0517	jittered	0.046	-0.379	0.452	0.568
TLI	300 km	TLI1037	jittered	0.048	-0.443	0.581	0.56
TLI	300 km	TLI0978	jittered	0.053	-0.1	0.203	0.711
TLI	300 km	TLI0925	jittered	0.055	-0.045	0.151	0.818
TLI	300 km	TLI1026	jittered	0.056	-0.385	0.481	0.582
TLI	300 km	TLI0642	jittered	0.057	-0.089	0.201	0.735
TLI	300 km	TLI0972	jittered	0.061	-0.44	0.564	0.576
TLI	300 km	TLI1005	jittered	0.064	-0.278	0.395	0.62
TLI	300 km	TLI0998	jittered	0.064	-0.327	0.462	0.603
TLI	300 km	TLI0992	jittered	0.067	-0.396	0.521	0.592
TLI	300 km	TLI0360	jittered	0.067	-0.396	0.53	0.592
TLI	300 km	TLI0766	jittered	0.067	-0.226	0.359	0.64
TLI	300 km	TLI1044	jittered	0.069	-0.145	0.285	0.694
TLI	300 km	TLI0938	jittered	0.07	-0.412	0.559	0.59
TLI	300 km	TLI0852	jittered	0.072	-0.291	0.439	0.624
TLI	300 km	TLI0991	jittered	0.076	-0.3	0.462	0.628
TLI	300 km	TLI1078	jittered	0.078	-0.127	0.3	0.719
TLI	300 km	TLI1018	jittered	0.089	-0.432	0.593	0.611
TLI	300 km	TLI0866	jittered	0.091	-0.168	0.338	0.717
TLI	300 km	TLI0907	jittered	0.093	-0.296	0.487	0.646
TLI	300 km	TLI0951	jittered	0.093	-0.358	0.571	0.625
TLI	300 km	TLI1115	jittered	0.098	-0.303	0.496	0.652
TLI	300 km	TLI0959	jittered	0.099	-0.362	0.58	0.633

TLI	300 km	TLI1073	jittered	0.1	-0.028	0.229	0.894
TLI	300 km	TLI1097	jittered	0.101	-0.253	0.453	0.678
TLI	300 km	TLI0682	jittered	0.103	-0.047	0.251	0.865
TLI	300 km	TLI0932	jittered	0.107	-0.434	0.625	0.627
TLI	300 km	TLI1002	jittered	0.107	-0.284	0.498	0.668
TLI	300 km	TLI0180	jittered	0.114	-0.243	0.461	0.698
TLI	300 km	TLI1102	jittered	0.115	-0.226	0.446	0.708
TLI	300 km	TLI0865	jittered	0.121	-0.361	0.621	0.652
TLI	300 km	TLI0845	jittered	0.122	-0.182	0.409	0.747
TLI	300 km	TLI1118	jittered	0.124	-0.368	0.618	0.655
TLI	300 km	TLI1071	jittered	0.128	-0.249	0.505	0.707
TLI	300 km	TLI1059	jittered	0.128	-0.088	0.344	0.826
TLI	300 km	TLI0913	jittered	0.128	-0.093	0.361	0.814
TLI	300 km	TLI0995	jittered	0.134	-0.287	0.584	0.69
TLI	300 km	TLI0513	jittered	0.137	-0.203	0.481	0.736
TLI	300 km	TLI1077	jittered	0.138	-0.006	0.283	0.937
TLI	300 km	TLI1035	jittered	0.14	-0.304	0.596	0.688
TLI	300 km	TLI0971	jittered	0.14	-0.369	0.648	0.672
TLI	300 km	TLI1004	jittered	0.146	-0.13	0.425	0.8
TLI	300 km	TLI1092	jittered	0.148	-0.203	0.49	0.753
TLI	300 km	TLI0751	jittered	0.153	0.017	0.277	0.97
TLI	300 km	TLI0990	jittered	0.154	-0.329	0.634	0.694
TLI	300 km	TLI0888	jittered	0.157	-0.318	0.636	0.701
TLI	300 km	TLI1070	jittered	0.157	-0.096	0.419	0.838
TLI	300 km	TLI0860	jittered	0.159	-0.016	0.325	0.93
TLI	300 km	TLI0877	jittered	0.16	-0.249	0.594	0.729
TLI	300 km	TLI0900	jittered	0.163	-0.331	0.637	0.707
TLI	300 km	TLI0935	jittered	0.163	-0.182	0.515	0.775
TLI	300 km	TLI0898	jittered	0.166	-0.062	0.393	0.877
TLI	300 km	TLI0549	jittered	0.168	-0.168	0.497	0.789
TLI	300 km	TLI0872	jittered	0.172	-0.131	0.469	0.82
TLI	300 km	TLI1053	jittered	0.174	0.019	0.33	0.964
TLI	300 km	TLI0882	jittered	0.178	-0.263	0.608	0.743
TLI	300 km	TLI1058	jittered	0.178	-0.07	0.429	0.874
TLI	300 km	TLI0155	jittered	0.184	-0.238	0.614	0.756
TLI	300 km	TLI0616	jittered	0.184	0.071	0.294	0.995
TLI	300 km	TLI0924	jittered	0.184	-0.239	0.6	0.757
TLI	300 km	TLI0904	jittered	0.184	-0.098	0.444	0.862
TLI	300 km	TLI0868	jittered	0.184	-0.309	0.702	0.719

TLI	300 km	TLI0983	jittered	0.187	-0.249	0.642	0.75
TLI	300 km	TLI1021	jittered	0.192	-0.308	0.679	0.732
TLI	300 km	TLI0889	jittered	0.196	-0.129	0.522	0.833
TLI	300 km	TLI0921	jittered	0.197	0.036	0.361	0.973
TLI	300 km	TLI1013	jittered	0.206	-0.159	0.562	0.822
TLI	300 km	TLI1020	jittered	0.207	-0.093	0.497	0.87
TLI	300 km	TLI1045	jittered	0.207	-0.104	0.523	0.854
TLI	300 km	TLI0179	jittered	0.212	-0.146	0.586	0.823
TLI	300 km	TLI0914	jittered	0.213	-0.089	0.517	0.869
TLI	300 km	TLI0982	jittered	0.213	-0.043	0.466	0.908
TLI	300 km	TLI0963	jittered	0.213	-0.097	0.519	0.867
TLI	300 km	TLI1024	jittered	0.214	-0.203	0.633	0.794
TLI	300 km	TLI1030	jittered	0.216	-0.298	0.746	0.745
TLI	300 km	TLI0917	jittered	0.218	-0.27	0.715	0.761
TLI	300 km	TLI0934	jittered	0.22	-0.158	0.579	0.83
TLI	300 km	TLI0988	jittered	0.225	-0.071	0.522	0.889
TLI	300 km	TLI1109	jittered	0.225	-0.204	0.64	0.8
TLI	300 km	TLI1061	jittered	0.225	0.067	0.388	0.987
TLI	300 km	TLI0378	jittered	0.229	-0.172	0.648	0.813
TLI	300 km	TLI1081	jittered	0.235	0.018	0.462	0.955
TLI	300 km	TLI1080	jittered	0.235	-0.129	0.589	0.851
TLI	300 km	TLI0985	jittered	0.236	-0.304	0.781	0.753
TLI	300 km	TLI0857	jittered	0.238	-0.225	0.664	0.801
TLI	300 km	TLI1032	jittered	0.238	-0.148	0.627	0.835
TLI	300 km	TLI0871	jittered	0.25	-0.109	0.6	0.872
TLI	300 km	TLI0918	jittered	0.256	-0.265	0.764	0.786
TLI	300 km	TLI0843	jittered	0.256	-0.215	0.713	0.812
TLI	300 km	TLI0929	jittered	0.256	0.052	0.444	0.981
TLI	300 km	TLI1046	jittered	0.256	-0.091	0.605	0.88
TLI	300 km	TLI0859	jittered	0.263	-0.187	0.741	0.818
TLI	300 km	TLI0994	jittered	0.263	-0.135	0.654	0.856
TLI	300 km	TLI0847	jittered	0.266	-0.23	0.741	0.809
TLI	300 km	TLI0986	jittered	0.275	-0.249	0.81	0.799
TLI	300 km	TLI0398	jittered	0.282	-0.183	0.724	0.838
TLI	300 km	TLI0873	jittered	0.287	-0.148	0.718	0.856
TLI	300 km	TLI0381	jittered	0.291	-0.124	0.687	0.875
TLI	300 km	TLI0401	jittered	0.295	-0.144	0.731	0.859
TLI	300 km	TLI0636	jittered	0.296	0.123	0.473	0.997
TLI	300 km	TLI1056	jittered	0.301	-0.087	0.669	0.9

TLI	300 km	TLI1025	jittered	0.302	-0.162	0.791	0.844
TLI	300 km	TLI0936	jittered	0.304	-0.051	0.643	0.917
TLI	300 km	TLI1068	jittered	0.309	0.215	0.399	1
TLI	300 km	TLI0981	jittered	0.318	-0.162	0.792	0.858
TLI	300 km	TLI0911	jittered	0.321	-0.185	0.849	0.837
TLI	300 km	TLI1084	jittered	0.324	-0.015	0.641	0.943
TLI	300 km	TLI0617	jittered	0.325	0.172	0.483	0.999
TLI	300 km	TLI0940	jittered	0.34	-0.062	0.747	0.913
TLI	300 km	TLI0980	jittered	0.351	0.004	0.706	0.946
TLI	300 km	TLI1064	jittered	0.351	-0.012	0.714	0.939
TLI	300 km	TLI0364	jittered	0.353	-0.07	0.782	0.907
TLI	300 km	TLI1091	jittered	0.357	0.217	0.498	1
TLI	300 km	TLI0604	jittered	0.363	-0.053	0.773	0.921
TLI	300 km	TLI0582	jittered	0.375	0.092	0.672	0.98
TLI	300 km	TLI1038	jittered	0.378	-0.093	0.841	0.903
TLI	300 km	TLI1105	jittered	0.38	-0.078	0.843	0.907
TLI	300 km	TLI0790	jittered	0.38	-0.024	0.805	0.93
TLI	300 km	TLI0120	jittered	0.382	0.141	0.623	0.994
TLI	300 km	TLI0952	jittered	0.382	0.175	0.583	0.999
TLI	300 km	TLI0842	jittered	0.383	-0.045	0.827	0.922
TLI	300 km	TLI1033	jittered	0.387	0.099	0.68	0.983
TLI	300 km	TLI1010	jittered	0.388	0.165	0.615	0.997
TLI	300 km	TLI1017	jittered	0.393	-0.038	0.819	0.929
TLI	300 km	TLI0993	jittered	0.396	-0.057	0.841	0.922
TLI	300 km	TLI0996	jittered	0.398	0.026	0.774	0.955
TLI	300 km	TLI1067	jittered	0.405	0.066	0.726	0.975
TLI	300 km	TLI0962	jittered	0.409	0.115	0.692	0.988
TLI	300 km	TLI1087	jittered	0.412	0.044	0.785	0.963
TLI	300 km	TLI0896	jittered	0.412	0.221	0.601	1
TLI	300 km	TLI0895	jittered	0.413	0.239	0.581	1
TLI	300 km	TLI1096	jittered	0.42	-0.041	0.897	0.925
TLI	300 km	TLI0968	jittered	0.437	0.117	0.777	0.983
TLI	300 km	TLI1031	jittered	0.438	0.038	0.839	0.96
TLI	300 km	TLI0673	jittered	0.445	0.182	0.714	0.996
TLI	300 km	TLI0884	jittered	0.458	0.017	0.87	0.957
TLI	300 km	TLI0431	jittered	0.479	0.096	0.847	0.98
TLI	300 km	TLI0801	jittered	0.479	0.076	0.882	0.972
TLI	300 km	TLI1093	jittered	0.48	0.08	0.866	0.975
TLI	300 km	TLI0592	jittered	0.49	0.24	0.742	0.999

TLI	300 km	TLI1027	jittered	0.495	-0.013	0.983	0.944
TLI	300 km	TLI0977	jittered	0.511	0.199	0.831	0.995
TLI	300 km	TLI0589	jittered	0.514	0.249	0.765	0.999
TLI	300 km	TLI0621	jittered	0.516	0.388	0.655	1
TLI	300 km	TLI0373	jittered	0.518	0.052	0.979	0.963
TLI	300 km	TLI0947	jittered	0.534	0.098	0.983	0.974
TLI	300 km	TLI0923	jittered	0.534	0.206	0.855	0.995
TLI	300 km	TLI1043	jittered	0.535	0.127	0.96	0.98
TLI	300 km	TLI0881	jittered	0.544	0.063	1.021	0.965
TLI	300 km	TLI0598	jittered	0.567	0.237	0.891	0.997
TLI	300 km	TLI0383	jittered	0.571	0.116	0.999	0.981
TLI	300 km	TLI0596	jittered	0.571	0.19	0.94	0.992
TLI	300 km	TLI1008	jittered	0.571	0.194	0.972	0.99
TLI	300 km	TLI1107	jittered	0.572	0.4	0.741	1
TLI	300 km	TLI0858	jittered	0.598	0.148	1.108	0.978
TLI	300 km	TLI0392	jittered	0.613	0.144	1.078	0.983
TLI	300 km	TLI1101	jittered	0.637	0.303	0.972	0.999
TLI	300 km	TLI1112	jittered	0.641	0.398	0.903	1
TLI	300 km	TLI0961	jittered	0.663	0.19	1.116	0.989
TLI	300 km	TLI1100	jittered	0.667	0.437	0.895	1
TLI	300 km	TLI0841	jittered	0.696	0.388	1.03	1
TLI	300 km	TLI1006	jittered	0.745	0.393	1.104	1
TLI	300 km	TLI0840	jittered	0.749	0.34	1.174	0.998
TLI	300 km	TLI0903	jittered	0.768	0.332	1.198	0.998
TLI	300 km	TLI0570	jittered	0.77	0.393	1.151	1
TLI	300 km	TLI1051	jittered	0.771	0.587	0.954	1
TLI	300 km	TLI0989	jittered	0.794	0.345	1.26	0.997
TLI	300 km	TLI0928	jittered	0.805	0.484	1.129	1
TLI	300 km	TLI1007	jittered	0.888	0.507	1.289	1
TLI	300 km	TLI0955	jittered	1.019	0.775	1.263	1
TLI	300 km	TLI0958	jittered	1.117	0.765	1.469	1
TLI	300 km	TLI0939	jittered	1.177	0.835	1.51	1
TLI	300 km	TLI1052	jittered	1.196	0.992	1.392	1
TLI	300 km	TLI0559	jittered	1.215	0.918	1.521	1
TLI	500 km	TLI1055	jittered	-1.721	-1.976	-1.462	1
TLI	500 km	TLI0855	jittered	-1.452	-1.782	-1.11	1
TLI	500 km	TLI1066	jittered	-1.088	-1.333	-0.843	1
TLI	500 km	TLI1063	jittered	-0.982	-1.416	-0.546	1
TLI	500 km	TLI0578	jittered	-0.951	-1.197	-0.713	1

TLI	500 km	TLI1069	jittered	-0.845	-1.081	-0.598	1
TLI	500 km	TLI1036	jittered	-0.841	-1.216	-0.472	1
TLI	500 km	TLI1016	jittered	-0.84	-1.11	-0.573	1
TLI	500 km	TLI1039	jittered	-0.829	-1.057	-0.602	1
TLI	500 km	TLI0102	jittered	-0.746	-1.158	-0.352	0.998
TLI	500 km	TLI1083	jittered	-0.679	-1.318	-0.045	0.956
TLI	500 km	TLI0966	jittered	-0.671	-0.959	-0.378	1
TLI	500 km	TLI0615	jittered	-0.665	-0.821	-0.5	1
TLI	500 km	TLI1060	jittered	-0.662	-0.875	-0.456	1
TLI	500 km	TLI0612	jittered	-0.646	-0.854	-0.437	1
TLI	500 km	TLI0512	jittered	-0.621	-0.975	-0.263	0.998
TLI	500 km	TLI0856	jittered	-0.612	-0.967	-0.24	0.996
TLI	500 km	TLI0908	jittered	-0.604	-0.944	-0.245	0.997
TLI	500 km	TLI0976	jittered	-0.604	-0.996	-0.22	0.994
TLI	500 km	TLI0861	jittered	-0.596	-0.906	-0.286	0.999
TLI	500 km	TLI1103	jittered	-0.58	-0.758	-0.404	1
TLI	500 km	TLI1099	jittered	-0.574	-0.828	-0.32	1
TLI	500 km	TLI0518	jittered	-0.565	-0.89	-0.234	0.997
TLI	500 km	TLI0453	jittered	-0.562	-0.719	-0.411	1
TLI	500 km	TLI1085	jittered	-0.549	-0.932	-0.164	0.988
TLI	500 km	TLI0635	jittered	-0.549	-0.713	-0.386	1
TLI	500 km	TLI1049	jittered	-0.546	-0.718	-0.367	1
TLI	500 km	TLI0893	jittered	-0.54	-0.743	-0.349	1
TLI	500 km	TLI0844	jittered	-0.537	-0.996	-0.091	0.971
TLI	500 km	TLI0670	jittered	-0.535	-0.944	-0.143	0.983
TLI	500 km	TLI0630	jittered	-0.527	-0.677	-0.378	1
TLI	500 km	TLI0954	jittered	-0.511	-0.872	-0.167	0.989
TLI	500 km	TLI1111	jittered	-0.497	-0.682	-0.319	1
TLI	500 km	TLI0631	jittered	-0.495	-0.685	-0.298	1
TLI	500 km	TLI1012	jittered	-0.494	-0.987	0.001	0.945
TLI	500 km	TLI0956	jittered	-0.482	-0.796	-0.15	0.992
TLI	500 km	TLI0974	jittered	-0.481	-1.008	0.052	0.926
TLI	500 km	TLI0979	jittered	-0.479	-0.641	-0.319	1
TLI	500 km	TLI1110	jittered	-0.458	-0.744	-0.157	0.994
TLI	500 km	TLI1011	jittered	-0.457	-0.826	-0.09	0.976
TLI	500 km	TLI0957	jittered	-0.431	-0.763	-0.106	0.982
TLI	500 km	TLI1057	jittered	-0.425	-0.757	-0.098	0.981
TLI	500 km	TLI0371	jittered	-0.425	-0.843	-0.008	0.947
TLI	500 km	TLI0164	jittered	-0.409	-0.79	-0.016	0.954

TLI	500 km	TLI1079	jittered	-0.406	-0.755	-0.069	0.97
TLI	500 km	TLI0804	jittered	-0.399	-0.677	-0.112	0.989
TLI	500 km	TLI0597	jittered	-0.388	-1.05	0.28	0.824
TLI	500 km	TLI0999	jittered	-0.372	-0.765	0.016	0.937
TLI	500 km	TLI0886	jittered	-0.369	-0.944	0.203	0.849
TLI	500 km	TLI1029	jittered	-0.366	-0.817	0.076	0.905
TLI	500 km	TLI0984	jittered	-0.362	-0.762	0.051	0.921
TLI	500 km	TLI0919	jittered	-0.349	-0.933	0.206	0.838
TLI	500 km	TLI1062	jittered	-0.344	-0.471	-0.216	1
TLI	500 km	TLI1075	jittered	-0.338	-0.532	-0.148	0.998
TLI	500 km	TLI1076	jittered	-0.334	-0.665	0.006	0.944
TLI	500 km	TLI1104	jittered	-0.331	-0.522	-0.135	0.997
TLI	500 km	TLI0096	jittered	-0.327	-0.653	0.004	0.944
TLI	500 km	TLI0107	jittered	-0.323	-0.686	0.051	0.921
TLI	500 km	TLI0933	jittered	-0.32	-0.685	0.039	0.921
TLI	500 km	TLI0081	jittered	-0.32	-0.919	0.28	0.803
TLI	500 km	TLI0891	jittered	-0.313	-0.54	-0.086	0.986
TLI	500 km	TLI0440	jittered	-0.312	-0.847	0.238	0.821
TLI	500 km	TLI1028	jittered	-0.307	-0.561	-0.051	0.974
TLI	500 km	TLI0140	jittered	-0.297	-0.634	0.038	0.92
TLI	500 km	TLI1094	jittered	-0.284	-0.758	0.162	0.837
TLI	500 km	TLI1040	jittered	-0.28	-0.865	0.302	0.779
TLI	500 km	TLI0608	jittered	-0.276	-0.431	-0.117	0.998
TLI	500 km	TLI0930	jittered	-0.272	-0.442	-0.096	0.994
TLI	500 km	TLI1019	jittered	-0.264	-0.696	0.16	0.838
TLI	500 km	TLI0580	jittered	-0.255	-0.653	0.158	0.841
TLI	500 km	TLI1023	jittered	-0.251	-0.829	0.33	0.758
TLI	500 km	TLI1065	jittered	-0.244	-0.398	-0.085	0.994
TLI	500 km	TLI0771	jittered	-0.242	-0.7	0.205	0.801
TLI	500 km	TLI1050	jittered	-0.241	-0.412	-0.07	0.988
TLI	500 km	TLI0514	jittered	-0.228	-0.62	0.151	0.828
TLI	500 km	TLI0975	jittered	-0.221	-0.689	0.246	0.772
TLI	500 km	TLI0374	jittered	-0.214	-0.633	0.216	0.786
TLI	500 km	TLI0388	jittered	-0.206	-0.656	0.233	0.771
TLI	500 km	TLI0792	jittered	-0.206	-0.56	0.147	0.825
TLI	500 km	TLI0964	jittered	-0.205	-0.51	0.09	0.864
TLI	500 km	TLI1003	jittered	-0.2	-0.754	0.34	0.722
TLI	500 km	TLI1089	jittered	-0.191	-0.577	0.179	0.791
TLI	500 km	TLI0902	jittered	-0.188	-0.582	0.205	0.777

TLI	500 km	TLI0867	jittered	-0.187	-0.429	0.044	0.898
TLI	500 km	TLI0519	jittered	-0.183	-0.585	0.222	0.763
TLI	500 km	TLI0874	jittered	-0.173	-0.51	0.17	0.794
TLI	500 km	TLI0915	jittered	-0.169	-0.478	0.14	0.811
TLI	500 km	TLI0912	jittered	-0.168	-0.688	0.346	0.699
TLI	500 km	TLI1022	jittered	-0.163	-0.484	0.153	0.793
TLI	500 km	TLI0400	jittered	-0.16	-0.592	0.281	0.718
TLI	500 km	TLI0960	jittered	-0.157	-0.441	0.127	0.813
TLI	500 km	TLI0879	jittered	-0.148	-0.746	0.444	0.653
TLI	500 km	TLI0892	jittered	-0.146	-0.396	0.104	0.826
TLI	500 km	TLI0848	jittered	-0.143	-0.271	-0.017	0.963
TLI	500 km	TLI0624	jittered	-0.139	-0.337	0.053	0.871
TLI	500 km	TLI0973	jittered	-0.139	-0.57	0.306	0.694
TLI	500 km	TLI0044	jittered	-0.137	-0.42	0.155	0.776
TLI	500 km	TLI0890	jittered	-0.131	-0.36	0.086	0.827
TLI	500 km	TLI0998	jittered	-0.122	-0.537	0.286	0.684
TLI	500 km	TLI0629	jittered	-0.121	-0.243	0.002	0.942
TLI	500 km	TLI1072	jittered	-0.112	-0.501	0.267	0.679
TLI	500 km	TLI0839	jittered	-0.107	-0.423	0.209	0.707
TLI	500 km	TLI1042	jittered	-0.105	-0.64	0.439	0.622
TLI	500 km	TLI0517	jittered	-0.105	-0.508	0.298	0.661
TLI	500 km	TLI0910	jittered	-0.104	-0.649	0.432	0.621
TLI	500 km	TLI0965	jittered	-0.103	-0.389	0.186	0.715
TLI	500 km	TLI0906	jittered	-0.101	-0.439	0.241	0.683
TLI	500 km	TLI1014	jittered	-0.101	-0.374	0.177	0.72
TLI	500 km	TLI1116	jittered	-0.09	-0.37	0.179	0.7
TLI	500 km	TLI0035	jittered	-0.09	-0.304	0.116	0.753
TLI	500 km	TLI0674	jittered	-0.087	-0.31	0.131	0.737
TLI	500 km	TLI0931	jittered	-0.086	-0.633	0.457	0.599
TLI	500 km	TLI0967	jittered	-0.083	-0.408	0.229	0.661
TLI	500 km	TLI0849	jittered	-0.071	-0.355	0.235	0.65
TLI	500 km	TLI0376	jittered	-0.071	-0.505	0.378	0.601
TLI	500 km	TLI0987	jittered	-0.067	-0.428	0.311	0.617
TLI	500 km	TLI0907	jittered	-0.057	-0.402	0.29	0.604
TLI	500 km	TLI1098	jittered	-0.057	-0.276	0.149	0.668
TLI	500 km	TLI0515	jittered	-0.056	-0.448	0.339	0.589
TLI	500 km	TLI1106	jittered	-0.047	-0.271	0.188	0.627
TLI	500 km	TLI1041	jittered	-0.046	-0.606	0.513	0.551
TLI	500 km	TLI0937	jittered	-0.04	-0.391	0.327	0.571

TLI	500 km	TLI0972	jittered	-0.032	-0.532	0.462	0.54
TLI	500 km	TLI0005	jittered	-0.03	-0.351	0.298	0.558
TLI	500 km	TLI0953	jittered	-0.028	-0.419	0.354	0.547
TLI	500 km	TLI0875	jittered	-0.026	-0.374	0.325	0.548
TLI	500 km	TLI0766	jittered	-0.022	-0.39	0.348	0.539
TLI	500 km	TLI0943	jittered	-0.021	-0.476	0.442	0.529
TLI	500 km	TLI1082	jittered	-0.019	-0.325	0.3	0.539
TLI	500 km	TLI1118	jittered	-0.016	-0.525	0.489	0.521
TLI	500 km	TLI1088	jittered	-0.014	-0.242	0.214	0.538
TLI	500 km	TLI0909	jittered	-0.012	-0.757	0.754	0.511
TLI	500 km	TLI0970	jittered	-0.012	-0.567	0.537	0.514
TLI	500 km	TLI0878	jittered	-0.01	-0.44	0.409	0.515
TLI	500 km	TLI1074	jittered	-0.009	-0.285	0.267	0.52
TLI	500 km	TLI0654	jittered	-0.004	-0.361	0.358	0.507
TLI	500 km	TLI0668	jittered	-0.003	-0.173	0.168	0.506
TLI	500 km	TLI1115	jittered	0.003	-0.431	0.447	0.505
TLI	500 km	TLI0880	jittered	0.01	-0.507	0.542	0.511
TLI	500 km	TLI1090	jittered	0.011	-0.18	0.211	0.538
TLI	500 km	TLI0672	jittered	0.011	-0.28	0.309	0.524
TLI	500 km	TLI1015	jittered	0.012	-0.098	0.12	0.571
TLI	500 km	TLI0916	jittered	0.013	-0.574	0.554	0.515
TLI	500 km	TLI0920	jittered	0.016	-0.566	0.618	0.517
TLI	500 km	TLI0885	jittered	0.018	-0.558	0.592	0.519
TLI	500 km	TLI0865	jittered	0.019	-0.481	0.506	0.523
TLI	500 km	TLI0738	jittered	0.02	-0.09	0.13	0.615
TLI	500 km	TLI0853	jittered	0.026	-0.4	0.467	0.538
TLI	500 km	TLI0971	jittered	0.027	-0.432	0.493	0.537
TLI	500 km	TLI0846	jittered	0.03	-0.13	0.187	0.618
TLI	500 km	TLI0640	jittered	0.03	-0.151	0.209	0.607
TLI	500 km	TLI0894	jittered	0.038	-0.143	0.213	0.634
TLI	500 km	TLI0155	jittered	0.038	-0.302	0.393	0.57
TLI	500 km	TLI0613	jittered	0.039	-0.16	0.242	0.623
TLI	500 km	TLI1000	jittered	0.043	-0.324	0.398	0.576
TLI	500 km	TLI1004	jittered	0.043	-0.245	0.318	0.598
TLI	500 km	TLI1009	jittered	0.043	-0.39	0.503	0.56
TLI	500 km	TLI1117	jittered	0.045	-0.345	0.434	0.575
TLI	500 km	TLI0925	jittered	0.048	-0.043	0.144	0.794
TLI	500 km	TLI0905	jittered	0.049	-0.281	0.394	0.592
TLI	500 km	TLI1037	jittered	0.05	-0.501	0.595	0.559

TLI	500 km	TLI0887	jittered	0.053	-0.538	0.622	0.556
TLI	500 km	TLI0899	jittered	0.062	-0.449	0.568	0.577
TLI	500 km	TLI1113	jittered	0.064	-0.113	0.236	0.721
TLI	500 km	TLI1044	jittered	0.066	-0.127	0.253	0.709
TLI	500 km	TLI1005	jittered	0.067	-0.272	0.417	0.623
TLI	500 km	TLI0877	jittered	0.074	-0.308	0.455	0.622
TLI	500 km	TLI1073	jittered	0.079	-0.054	0.215	0.826
TLI	500 km	TLI1002	jittered	0.091	-0.323	0.482	0.641
TLI	500 km	TLI1045	jittered	0.091	-0.226	0.411	0.677
TLI	500 km	TLI1059	jittered	0.092	-0.165	0.34	0.72
TLI	500 km	TLI0991	jittered	0.096	-0.293	0.475	0.655
TLI	500 km	TLI1021	jittered	0.103	-0.364	0.565	0.639
TLI	500 km	TLI0360	jittered	0.112	-0.278	0.518	0.672
TLI	500 km	TLI0513	jittered	0.116	-0.208	0.441	0.717
TLI	500 km	TLI0852	jittered	0.119	-0.206	0.44	0.721
TLI	500 km	TLI1078	jittered	0.122	-0.073	0.314	0.844
TLI	500 km	TLI1097	jittered	0.127	-0.222	0.463	0.725
TLI	500 km	TLI0983	jittered	0.129	-0.307	0.582	0.68
TLI	500 km	TLI1035	jittered	0.14	-0.297	0.566	0.7
TLI	500 km	TLI0845	jittered	0.141	-0.179	0.472	0.754
TLI	500 km	TLI0995	jittered	0.144	-0.307	0.614	0.691
TLI	500 km	TLI0963	jittered	0.149	-0.132	0.426	0.802
TLI	500 km	TLI0932	jittered	0.15	-0.388	0.669	0.675
TLI	500 km	TLI0682	jittered	0.151	-0.015	0.312	0.93
TLI	500 km	TLI0898	jittered	0.152	-0.067	0.367	0.87
TLI	500 km	TLI0913	jittered	0.156	-0.072	0.377	0.867
TLI	500 km	TLI0988	jittered	0.156	-0.083	0.405	0.846
TLI	500 km	TLI1024	jittered	0.156	-0.261	0.572	0.728
TLI	500 km	TLI0850	jittered	0.157	-0.404	0.713	0.673
TLI	500 km	TLI0945	jittered	0.159	-0.319	0.619	0.707
TLI	500 km	TLI0616	jittered	0.16	0.031	0.288	0.977
TLI	500 km	TLI0560	jittered	0.162	-0.335	0.64	0.701
TLI	500 km	TLI0866	jittered	0.163	-0.055	0.377	0.887
TLI	500 km	TLI1080	jittered	0.164	-0.225	0.556	0.749
TLI	500 km	TLI0992	jittered	0.165	-0.328	0.651	0.706
TLI	500 km	TLI0901	jittered	0.166	-0.33	0.659	0.703
TLI	500 km	TLI0370	jittered	0.173	-0.267	0.617	0.734
TLI	500 km	TLI1013	jittered	0.173	-0.194	0.524	0.78
TLI	500 km	TLI0978	jittered	0.174	0.009	0.338	0.954

TLI	500 km	TLI1061	jittered	0.181	0.026	0.331	0.97
TLI	500 km	TLI1077	jittered	0.184	0.033	0.335	0.974
TLI	500 km	TLI1026	jittered	0.194	-0.197	0.59	0.785
TLI	500 km	TLI0430	jittered	0.194	-0.256	0.672	0.747
TLI	500 km	TLI0882	jittered	0.195	-0.22	0.615	0.772
TLI	500 km	TLI0860	jittered	0.195	0.032	0.355	0.972
TLI	500 km	TLI1053	jittered	0.195	0.004	0.381	0.951
TLI	500 km	TLI0935	jittered	0.198	-0.145	0.533	0.825
TLI	500 km	TLI0642	jittered	0.2	0.035	0.364	0.974
TLI	500 km	TLI1018	jittered	0.202	-0.355	0.756	0.719
TLI	500 km	TLI0180	jittered	0.203	-0.132	0.524	0.839
TLI	500 km	TLI1081	jittered	0.204	-0.007	0.419	0.936
TLI	500 km	TLI1047	jittered	0.206	-0.182	0.599	0.799
TLI	500 km	TLI0951	jittered	0.208	-0.24	0.65	0.773
TLI	500 km	TLI0751	jittered	0.208	0.071	0.341	0.993
TLI	500 km	TLI0982	jittered	0.209	-0.048	0.469	0.901
TLI	500 km	TLI0888	jittered	0.217	-0.237	0.669	0.779
TLI	500 km	TLI1030	jittered	0.223	-0.268	0.723	0.764
TLI	500 km	TLI0552	jittered	0.224	-0.049	0.488	0.91
TLI	500 km	TLI0179	jittered	0.233	-0.091	0.563	0.872
TLI	500 km	TLI0959	jittered	0.238	-0.218	0.691	0.799
TLI	500 km	TLI0873	jittered	0.252	-0.194	0.692	0.819
TLI	500 km	TLI0990	jittered	0.254	-0.179	0.694	0.823
TLI	500 km	TLI1020	jittered	0.257	-0.021	0.53	0.931
TLI	500 km	TLI1070	jittered	0.257	-0.035	0.547	0.92
TLI	500 km	TLI0889	jittered	0.258	-0.068	0.578	0.899
TLI	500 km	TLI0872	jittered	0.272	-0.002	0.545	0.943
TLI	500 km	TLI1102	jittered	0.275	-0.058	0.6	0.908
TLI	500 km	TLI1068	jittered	0.295	0.191	0.405	1
TLI	500 km	TLI0917	jittered	0.295	-0.213	0.815	0.821
TLI	500 km	TLI1092	jittered	0.296	-0.025	0.599	0.935
TLI	500 km	TLI0914	jittered	0.306	0.01	0.608	0.949
TLI	500 km	TLI1058	jittered	0.307	0.019	0.591	0.957
TLI	500 km	TLI1084	jittered	0.314	-0.009	0.634	0.941
TLI	500 km	TLI0904	jittered	0.316	0.05	0.584	0.971
TLI	500 km	TLI0582	jittered	0.319	0.04	0.592	0.968
TLI	500 km	TLI0921	jittered	0.32	0.177	0.464	1
TLI	500 km	TLI0378	jittered	0.321	-0.087	0.712	0.901
TLI	500 km	TLI0929	jittered	0.324	0.129	0.512	0.997

TLI	500 km	TLI0871	jittered	0.33	-0.042	0.708	0.921
TLI	500 km	TLI1105	jittered	0.333	-0.09	0.74	0.902
TLI	500 km	TLI0900	jittered	0.337	-0.176	0.843	0.856
TLI	500 km	TLI0549	jittered	0.338	0.039	0.634	0.966
TLI	500 km	TLI0994	jittered	0.349	-0.077	0.788	0.902
TLI	500 km	TLI1064	jittered	0.351	0.012	0.702	0.949
TLI	500 km	TLI1071	jittered	0.354	-0.035	0.746	0.926
TLI	500 km	TLI0940	jittered	0.356	-0.069	0.814	0.902
TLI	500 km	TLI0895	jittered	0.367	0.191	0.537	1
TLI	500 km	TLI1046	jittered	0.373	0.016	0.717	0.956
TLI	500 km	TLI0924	jittered	0.377	-0.079	0.806	0.914
TLI	500 km	TLI0962	jittered	0.381	0.09	0.661	0.983
TLI	500 km	TLI1032	jittered	0.382	0.014	0.739	0.953
TLI	500 km	TLI0617	jittered	0.383	0.197	0.574	0.999
TLI	500 km	TLI0364	jittered	0.386	-0.017	0.798	0.935
TLI	500 km	TLI0857	jittered	0.392	-0.008	0.82	0.935
TLI	500 km	TLI0938	jittered	0.393	-0.114	0.894	0.894
TLI	500 km	TLI0401	jittered	0.395	-0.052	0.839	0.922
TLI	500 km	TLI1038	jittered	0.396	-0.034	0.828	0.928
TLI	500 km	TLI0868	jittered	0.398	-0.14	0.913	0.887
TLI	500 km	TLI0636	jittered	0.399	0.196	0.59	0.999
TLI	500 km	TLI0934	jittered	0.402	0.077	0.721	0.977
TLI	500 km	TLI1091	jittered	0.404	0.271	0.539	1
TLI	500 km	TLI0120	jittered	0.409	0.181	0.64	0.998
TLI	500 km	TLI0859	jittered	0.411	-0.047	0.873	0.924
TLI	500 km	TLI1010	jittered	0.419	0.145	0.688	0.994
TLI	500 km	TLI1109	jittered	0.42	0.015	0.808	0.955
TLI	500 km	TLI0847	jittered	0.438	-0.017	0.897	0.937
TLI	500 km	TLI0918	jittered	0.441	-0.114	1.01	0.896
TLI	500 km	TLI0980	jittered	0.446	0.111	0.781	0.984
TLI	500 km	TLI0981	jittered	0.453	-0.064	0.957	0.922
TLI	500 km	TLI0843	jittered	0.471	-0.01	0.973	0.937
TLI	500 km	TLI0673	jittered	0.481	0.206	0.761	0.997
TLI	500 km	TLI0381	jittered	0.498	0.09	0.901	0.975
TLI	500 km	TLI1017	jittered	0.498	0.077	0.909	0.973
TLI	500 km	TLI0985	jittered	0.509	-0.016	1.033	0.94
TLI	500 km	TLI0996	jittered	0.518	0.124	0.908	0.982
TLI	500 km	TLI0936	jittered	0.519	0.159	0.87	0.99
TLI	500 km	TLI0986	jittered	0.53	0.049	1.026	0.958

TLI	500 km	TLI1067	jittered	0.543	0.195	0.893	0.994
TLI	500 km	TLI0592	jittered	0.553	0.329	0.784	1
TLI	500 km	TLI0911	jittered	0.554	-0.004	1.117	0.943
TLI	500 km	TLI0896	jittered	0.555	0.353	0.754	1
TLI	500 km	TLI0621	jittered	0.563	0.438	0.687	1
TLI	500 km	TLI0952	jittered	0.567	0.361	0.766	1
TLI	500 km	TLI1087	jittered	0.574	0.228	0.919	0.996
TLI	500 km	TLI1056	jittered	0.583	0.25	0.912	0.997
TLI	500 km	TLI0398	jittered	0.583	0.147	1.039	0.982
TLI	500 km	TLI1101	jittered	0.585	0.293	0.871	0.999
TLI	500 km	TLI1107	jittered	0.594	0.393	0.795	1
TLI	500 km	TLI1093	jittered	0.597	0.207	0.984	0.993
TLI	500 km	TLI1025	jittered	0.597	0.199	1.001	0.991
TLI	500 km	TLI1033	jittered	0.603	0.367	0.846	1
TLI	500 km	TLI0598	jittered	0.614	0.283	0.937	0.999
TLI	500 km	TLI0589	jittered	0.617	0.401	0.834	1
TLI	500 km	TLI0842	jittered	0.632	0.209	1.074	0.991
TLI	500 km	TLI0993	jittered	0.636	0.203	1.07	0.99
TLI	500 km	TLI0790	jittered	0.636	0.278	0.984	0.998
TLI	500 km	TLI0431	jittered	0.649	0.28	1.022	0.997
TLI	500 km	TLI0881	jittered	0.65	0.15	1.132	0.983
TLI	500 km	TLI1043	jittered	0.658	0.251	1.072	0.994
TLI	500 km	TLI0968	jittered	0.661	0.39	0.933	1
TLI	500 km	TLI0947	jittered	0.661	0.248	1.075	0.995
TLI	500 km	TLI0801	jittered	0.674	0.312	1.028	0.999
TLI	500 km	TLI0923	jittered	0.674	0.31	1.044	0.998
TLI	500 km	TLI0596	jittered	0.675	0.337	0.997	0.999
TLI	500 km	TLI0977	jittered	0.684	0.383	0.994	1
TLI	500 km	TLI1031	jittered	0.72	0.341	1.094	0.999
TLI	500 km	TLI0884	jittered	0.736	0.307	1.16	0.997
TLI	500 km	TLI1100	jittered	0.738	0.47	1.005	1
TLI	500 km	TLI0604	jittered	0.747	0.256	1.267	0.992
TLI	500 km	TLI1096	jittered	0.748	0.285	1.2	0.996
TLI	500 km	TLI1112	jittered	0.78	0.567	1	1
TLI	500 km	TLI0373	jittered	0.789	0.29	1.258	0.996
TLI	500 km	TLI0383	jittered	0.832	0.397	1.264	0.999
TLI	500 km	TLI0392	jittered	0.834	0.361	1.308	0.998
TLI	500 km	TLI1008	jittered	0.838	0.478	1.187	1
TLI	500 km	TLI1027	jittered	0.841	0.337	1.361	0.995

TLI	500 km	TLI0858	jittered	0.858	0.373	1.33	0.998
TLI	500 km	TLI0961	jittered	0.903	0.429	1.351	0.999
TLI	500 km	TLI1051	jittered	0.926	0.715	1.143	1
TLI	500 km	TLI0841	jittered	0.939	0.6	1.269	1
TLI	500 km	TLI0570	jittered	0.96	0.58	1.331	1
TLI	500 km	TLI0840	jittered	1.042	0.62	1.441	1
TLI	500 km	TLI0928	jittered	1.075	0.731	1.413	1
TLI	500 km	TLI0903	jittered	1.09	0.656	1.509	1
TLI	500 km	TLI1006	jittered	1.092	0.783	1.409	1
TLI	500 km	TLI0955	jittered	1.113	0.859	1.378	1
TLI	500 km	TLI1007	jittered	1.201	0.825	1.567	1
TLI	500 km	TLI0989	jittered	1.273	0.809	1.734	1
TLI	500 km	TLI1052	jittered	1.358	1.132	1.587	1
TLI	500 km	TLI0559	jittered	1.42	1.106	1.739	1
TLI	500 km	TLI0958	jittered	1.439	1.026	1.849	1
TLI	500 km	TLI0939	jittered	1.45	1.109	1.798	1
GBI	300 km	GB192c	jittered	-1.25	-1.497	-1.006	1
GBI	300 km	GB321c	jittered	-1.085	-1.311	-0.854	1
GBI	300 km	GB030	jittered	-1.079	-1.283	-0.883	1
GBI	300 km	GB167	jittered	-0.993	-1.191	-0.803	1
GBI	300 km	GB326	jittered	-0.99	-1.202	-0.773	1
GBI	300 km	GB703drmc	jittered	-0.825	-1.137	-0.497	1
GBI	300 km	GB041c	jittered	-0.81	-0.958	-0.661	1
GBI	300 km	GB110cC	jittered	-0.809	-0.959	-0.663	1
GBI	300 km	GB039c	jittered	-0.753	-0.961	-0.525	1
GBI	300 km	GB159	jittered	-0.734	-0.954	-0.515	1
GBI	300 km	GB254c	jittered	-0.699	-0.941	-0.44	1
GBI	300 km	GB947mc	jittered	-0.689	-0.96	-0.416	1
GBI	300 km	GB126	jittered	-0.65	-0.857	-0.447	1
GBI	300 km	GB313	jittered	-0.546	-0.762	-0.347	1
GBI	300 km	GB252c	jittered	-0.542	-0.844	-0.254	0.998
GBI	300 km	GB075c	jittered	-0.54	-0.787	-0.303	1
GBI	300 km	GB590mC	jittered	-0.527	-0.768	-0.283	1
GBI	300 km	GB256	jittered	-0.483	-0.662	-0.298	1
GBI	300 km	GB257	jittered	-0.472	-0.708	-0.237	0.999
GBI	300 km	GB887drmc	jittered	-0.433	-0.605	-0.266	1
GBI	300 km	GB300	jittered	-0.426	-0.599	-0.248	1
GBI	300 km	GB296	jittered	-0.414	-0.661	-0.184	0.997
GBI	300 km	GB150	jittered	-0.407	-0.618	-0.203	0.999

GBI	300 km	GB553drmcC	jittered	-0.403	-0.632	-0.175	0.997
GBI	300 km	GB137	jittered	-0.4	-0.626	-0.191	0.998
GBI	300 km	GB291c	jittered	-0.385	-0.682	-0.086	0.981
GBI	300 km	GB138	jittered	-0.368	-0.57	-0.16	0.998
GBI	300 km	GB591mC	jittered	-0.325	-0.512	-0.131	0.996
GBI	300 km	GB038c	jittered	-0.315	-0.71	0.074	0.899
GBI	300 km	GB329c	jittered	-0.308	-0.681	0.074	0.905
GBI	300 km	GB402	jittered	-0.287	-0.424	-0.149	0.999
GBI	300 km	GB500drm	jittered	-0.282	-0.481	-0.072	0.985
GBI	300 km	GB335	jittered	-0.269	-0.454	-0.082	0.99
GBI	300 km	GB325	jittered	-0.253	-0.41	-0.097	0.994
GBI	300 km	GB123	jittered	-0.252	-0.464	-0.043	0.97
GBI	300 km	GB501drm	jittered	-0.25	-0.482	-0.014	0.956
GBI	300 km	GB951drmcC	jittered	-0.247	-0.446	-0.052	0.977
GBI	300 km	GB480m	jittered	-0.247	-0.465	-0.023	0.962
GBI	300 km	GB152	jittered	-0.246	-0.439	-0.049	0.978
GBI	300 km	GB134	jittered	-0.244	-0.349	-0.139	0.999
GBI	300 km	GB800EO	jittered	-0.242	-0.578	0.097	0.872
GBI	300 km	GB084c	jittered	-0.241	-0.444	-0.036	0.971
GBI	300 km	GB949m	jittered	-0.238	-0.447	-0.032	0.967
GBI	300 km	GB704drm	jittered	-0.238	-0.444	-0.019	0.964
GBI	300 km	GB059	jittered	-0.232	-0.43	-0.041	0.97
GBI	300 km	GB057c	jittered	-0.231	-0.582	0.117	0.855
GBI	300 km	GB309c	jittered	-0.23	-0.419	-0.044	0.974
GBI	300 km	GB155C	jittered	-0.225	-0.402	-0.047	0.978
GBI	300 km	GB022c	jittered	-0.219	-0.487	0.043	0.906
GBI	300 km	GB197C	jittered	-0.217	-0.536	0.107	0.859
GBI	300 km	GB111C	jittered	-0.216	-0.382	-0.05	0.982
GBI	300 km	GB299	jittered	-0.215	-0.439	0.005	0.939
GBI	300 km	GB129	jittered	-0.203	-0.371	-0.031	0.971
GBI	300 km	GB082cC	jittered	-0.191	-0.421	0.044	0.906
GBI	300 km	GB049C	jittered	-0.188	-0.386	0.017	0.932
GBI	300 km	GB133C	jittered	-0.188	-0.49	0.112	0.841
GBI	300 km	GB081	jittered	-0.184	-0.355	-0.009	0.956
GBI	300 km	GB204	jittered	-0.182	-0.428	0.054	0.886
GBI	300 km	GB421c	jittered	-0.181	-0.436	0.081	0.868
GBI	300 km	GB522C	jittered	-0.176	-0.364	0.012	0.933
GBI	300 km	GB403	jittered	-0.174	-0.371	0.03	0.917
GBI	300 km	GB113	jittered	-0.173	-0.374	0.032	0.912

GBI	300 km	GB262c	jittered	-0.173	-0.437	0.088	0.854
GBI	300 km	GB107C	jittered	-0.164	-0.385	0.048	0.887
GBI	300 km	GB250	jittered	-0.164	-0.397	0.078	0.864
GBI	300 km	GB422c	jittered	-0.161	-0.409	0.076	0.855
GBI	300 km	GB606m	jittered	-0.151	-0.329	0.031	0.908
GBI	300 km	GB415	jittered	-0.147	-0.322	0.023	0.914
GBI	300 km	GB158	jittered	-0.145	-0.325	0.03	0.905
GBI	300 km	GB135	jittered	-0.145	-0.299	0.005	0.937
GBI	300 km	GB558drm	jittered	-0.144	-0.269	-0.019	0.967
GBI	300 km	GB140	jittered	-0.14	-0.357	0.089	0.844
GBI	300 km	GB625mC	jittered	-0.133	-0.335	0.07	0.854
GBI	300 km	GB433c	jittered	-0.129	-0.361	0.092	0.82
GBI	300 km	GB702drmC	jittered	-0.129	-0.402	0.148	0.775
GBI	300 km	GB068	jittered	-0.124	-0.338	0.086	0.825
GBI	300 km	GB297	jittered	-0.122	-0.337	0.089	0.82
GBI	300 km	GB286c	jittered	-0.121	-0.435	0.203	0.727
GBI	300 km	GB069	jittered	-0.12	-0.293	0.048	0.871
GBI	300 km	GB139	jittered	-0.119	-0.318	0.07	0.837
GBI	300 km	GB567drm	jittered	-0.109	-0.289	0.067	0.835
GBI	300 km	GB302c	jittered	-0.107	-0.331	0.106	0.786
GBI	300 km	GB104	jittered	-0.102	-0.324	0.113	0.772
GBI	300 km	GB431c	jittered	-0.095	-0.34	0.166	0.727
GBI	300 km	GB263c	jittered	-0.092	-0.345	0.161	0.719
GBI	300 km	GB108	jittered	-0.088	-0.307	0.142	0.734
GBI	300 km	GB054c	jittered	-0.087	-0.335	0.164	0.71
GBI	300 km	GB028	jittered	-0.081	-0.26	0.092	0.769
GBI	300 km	GB074c	jittered	-0.08	-0.345	0.183	0.685
GBI	300 km	GB099	jittered	-0.067	-0.203	0.068	0.787
GBI	300 km	GB301	jittered	-0.057	-0.249	0.126	0.688
GBI	300 km	GB950m	jittered	-0.041	-0.271	0.182	0.612
GBI	300 km	GB023c	jittered	-0.03	-0.277	0.229	0.575
GBI	300 km	GB196C	jittered	-0.027	-0.399	0.343	0.547
GBI	300 km	GB432c	jittered	-0.023	-0.231	0.193	0.569
GBI	300 km	GB260	jittered	-0.017	-0.144	0.109	0.588
GBI	300 km	GB048	jittered	-0.008	-0.194	0.18	0.528
GBI	300 km	GB334	jittered	-0.002	-0.236	0.233	0.504
GBI	300 km	GB622drmc	jittered	0.003	-0.214	0.221	0.509
GBI	300 km	GB035	jittered	0.005	-0.207	0.218	0.516
GBI	300 km	GB550m	jittered	0.009	-0.189	0.201	0.529

GBI	300 km	GB147c	jittered	0.014	-0.237	0.266	0.535
GBI	300 km	GB136	jittered	0.02	-0.154	0.202	0.572
GBI	300 km	GB118	jittered	0.02	-0.216	0.261	0.55
GBI	300 km	GB253c	jittered	0.022	-0.227	0.28	0.555
GBI	300 km	GB620drmc	jittered	0.024	-0.182	0.221	0.576
GBI	300 km	GB322c	jittered	0.027	-0.377	0.408	0.544
GBI	300 km	GB095C	jittered	0.036	-0.119	0.182	0.651
GBI	300 km	GB298	jittered	0.038	-0.075	0.154	0.706
GBI	300 km	GB569drmcC	jittered	0.048	-0.287	0.374	0.591
GBI	300 km	GB065e	jittered	0.054	-0.168	0.275	0.65
GBI	300 km	GB852drmC	jittered	0.072	-0.147	0.293	0.7
GBI	300 km	GB146	jittered	0.073	-0.145	0.289	0.705
GBI	300 km	GB549drmc	jittered	0.074	-0.073	0.226	0.786
GBI	300 km	GB103	jittered	0.083	-0.122	0.294	0.738
GBI	300 km	GB323c	jittered	0.089	-0.191	0.355	0.697
GBI	300 km	GB285cc	jittered	0.092	-0.423	0.623	0.609
GBI	300 km	GB327	jittered	0.094	-0.051	0.236	0.853
GBI	300 km	GB556EO	jittered	0.095	-0.351	0.547	0.632
GBI	300 km	GB551mC	jittered	0.097	-0.097	0.298	0.786
GBI	300 km	GB332EON	jittered	0.098	-0.038	0.239	0.87
GBI	300 km	GB331C	jittered	0.106	-0.024	0.236	0.905
GBI	300 km	GB127	jittered	0.111	-0.079	0.304	0.823
GBI	300 km	GB850drm	jittered	0.12	-0.022	0.256	0.916
GBI	300 km	GB430c	jittered	0.125	-0.087	0.337	0.824
GBI	300 km	GB900EO	jittered	0.137	0.02	0.258	0.967
GBI	300 km	GB265	jittered	0.142	-0.059	0.344	0.869
GBI	300 km	GB304c	jittered	0.142	-0.106	0.384	0.822
GBI	300 km	GB624drmc	jittered	0.146	-0.084	0.383	0.841
GBI	300 km	GB701drm	jittered	0.149	-0.049	0.346	0.885
GBI	300 km	GB149C	jittered	0.161	-0.028	0.358	0.908
GBI	300 km	GB853drm	jittered	0.167	-0.06	0.381	0.887
GBI	300 km	GB046C	jittered	0.169	0.008	0.328	0.953
GBI	300 km	GB621drmc	jittered	0.175	-0.037	0.388	0.907
GBI	300 km	GB052c	jittered	0.177	-0.047	0.402	0.895
GBI	300 km	GB623drmc	jittered	0.179	-0.061	0.403	0.891
GBI	300 km	GB995F	jittered	0.18	0.048	0.315	0.984
GBI	300 km	GB955drmc	jittered	0.181	0.067	0.296	0.993
GBI	300 km	GB098C	jittered	0.194	0.027	0.358	0.969
GBI	300 km	GB559drm	jittered	0.204	0.106	0.306	0.998

GBI	300 km	GB273	jittered	0.213	-0.012	0.428	0.939
GBI	300 km	GB330c	jittered	0.227	-0.134	0.61	0.833
GBI	300 km	GB888drmcC	jittered	0.227	-0.023	0.47	0.93
GBI	300 km	GB116	jittered	0.229	0.084	0.372	0.994
GBI	300 km	GB026	jittered	0.232	0.042	0.431	0.972
GBI	300 km	GB954drmcC	jittered	0.249	0.078	0.426	0.989
GBI	300 km	GB777drm	jittered	0.258	0.042	0.476	0.971
GBI	300 km	GB264c	jittered	0.27	0.073	0.469	0.985
GBI	300 km	GB401	jittered	0.272	0.068	0.467	0.985
GBI	300 km	GB151	jittered	0.283	0.134	0.432	0.998
GBI	300 km	GB083c	jittered	0.289	0.128	0.453	0.997
GBI	300 km	GB563m	jittered	0.29	0.143	0.427	0.999
GBI	300 km	GB037	jittered	0.291	0.185	0.398	1
GBI	300 km	GB132	jittered	0.292	0.062	0.508	0.981
GBI	300 km	GB027	jittered	0.301	0.106	0.495	0.993
GBI	300 km	GB109C	jittered	0.304	0.061	0.558	0.974
GBI	300 km	GB568drm	jittered	0.311	0.117	0.5	0.995
GBI	300 km	GB117	jittered	0.317	0.16	0.468	1
GBI	300 km	GB556m	jittered	0.36	0.202	0.519	1
GBI	300 km	GB408	jittered	0.37	0.158	0.583	0.997
GBI	300 km	GB051c	jittered	0.395	0.171	0.62	0.997
GBI	300 km	GB510m	jittered	0.404	0.195	0.614	0.999
GBI	300 km	GB156	jittered	0.416	0.245	0.586	1
GBI	300 km	GB555m	jittered	0.442	0.248	0.636	1
GBI	300 km	GB410	jittered	0.445	0.259	0.62	1
GBI	300 km	GB020c	jittered	0.445	0.249	0.638	1
GBI	300 km	GB105	jittered	0.461	0.265	0.67	1
GBI	300 km	GB131	jittered	0.468	0.253	0.689	1
GBI	300 km	GB560drmcC	jittered	0.475	0.248	0.694	1
GBI	300 km	GB251m	jittered	0.482	0.276	0.684	1
GBI	300 km	GB058C	jittered	0.51	0.247	0.786	0.998
GBI	300 km	GB990drmcC	jittered	0.516	0.306	0.728	1
GBI	300 km	GB031	jittered	0.517	0.348	0.685	1
GBI	300 km	GB177	jittered	0.526	0.37	0.693	1
GBI	300 km	GB953drmcC	jittered	0.534	0.048	1.028	0.959
GBI	300 km	GB324	jittered	0.574	0.391	0.757	1
GBI	300 km	GB595m	jittered	0.581	0.404	0.761	1
GBI	300 km	GB024e	jittered	0.623	0.364	0.885	1
GBI	300 km	GB130e	jittered	0.673	0.443	0.915	1

GBI	300 km	GB203e	jittered	0.675	0.472	0.88	1
GBI	300 km	GB198c	jittered	0.676	0.411	0.924	1
GBI	300 km	GB047	jittered	0.679	0.517	0.841	1
GBI	300 km	GB122	jittered	0.691	0.513	0.866	1
GBI	300 km	GB053c	jittered	0.7	0.492	0.902	1
GBI	300 km	GB952drmcC	jittered	0.71	0.512	0.905	1
GBI	300 km	GB557drm	jittered	0.725	0.562	0.901	1
GBI	300 km	GB945m	jittered	0.739	0.549	0.918	1
GBI	300 km	GB991drmcC	jittered	0.745	0.54	0.942	1
GBI	300 km	GB036	jittered	0.756	0.557	0.958	1
GBI	300 km	GB021c	jittered	0.774	0.579	0.965	1
GBI	300 km	GB552m	jittered	0.778	0.572	0.989	1
GBI	300 km	GB096C	jittered	0.794	0.64	0.954	1
GBI	300 km	GB800m	jittered	0.8	0.598	1	1
GBI	300 km	GB561drmc	jittered	0.814	0.623	1.005	1
GBI	300 km	GB409	jittered	0.98	0.757	1.197	1
GBI	300 km	GB124	jittered	1.141	0.943	1.343	1
GBI	500 km	GB192c	jittered	-1.396	-1.608	-1.187	1
GBI	500 km	GB030	jittered	-1.131	-1.339	-0.915	1
GBI	500 km	GB167	jittered	-1.023	-1.234	-0.81	1
GBI	500 km	GB326	jittered	-1.003	-1.224	-0.772	1
GBI	500 km	GB041c	jittered	-0.976	-1.162	-0.794	1
GBI	500 km	GB703drmc	jittered	-0.919	-1.202	-0.631	1
GBI	500 km	GB254c	jittered	-0.867	-1.105	-0.622	1
GBI	500 km	GB110cC	jittered	-0.859	-1.024	-0.684	1
GBI	500 km	GB321c	jittered	-0.853	-1.126	-0.574	1
GBI	500 km	GB252c	jittered	-0.791	-1.044	-0.549	1
GBI	500 km	GB947mc	jittered	-0.777	-1.056	-0.509	1
GBI	500 km	GB039c	jittered	-0.668	-0.901	-0.439	1
GBI	500 km	GB257	jittered	-0.63	-0.86	-0.384	1
GBI	500 km	GB075c	jittered	-0.627	-0.882	-0.374	1
GBI	500 km	GB126	jittered	-0.624	-0.851	-0.409	1
GBI	500 km	GB590mC	jittered	-0.604	-0.837	-0.365	1
GBI	500 km	GB313	jittered	-0.604	-0.822	-0.397	1
GBI	500 km	GB159	jittered	-0.558	-0.792	-0.333	1
GBI	500 km	GB291c	jittered	-0.512	-0.799	-0.229	0.998
GBI	500 km	GB329c	jittered	-0.491	-0.847	-0.146	0.987
GBI	500 km	GB300	jittered	-0.474	-0.682	-0.271	1
GBI	500 km	GB038c	jittered	-0.395	-0.777	-0.017	0.952

GBI	500 km	GB256	jittered	-0.392	-0.591	-0.192	0.999
GBI	500 km	GB591mC	jittered	-0.39	-0.612	-0.172	0.997
GBI	500 km	GB887drmc	jittered	-0.389	-0.579	-0.188	0.999
GBI	500 km	GB137	jittered	-0.378	-0.611	-0.16	0.996
GBI	500 km	GB335	jittered	-0.334	-0.526	-0.141	0.997
GBI	500 km	GB197C	jittered	-0.317	-0.654	0.009	0.937
GBI	500 km	GB150	jittered	-0.315	-0.533	-0.092	0.988
GBI	500 km	GB022c	jittered	-0.311	-0.569	-0.045	0.97
GBI	500 km	GB325	jittered	-0.3	-0.45	-0.143	0.998
GBI	500 km	GB134	jittered	-0.293	-0.416	-0.167	1
GBI	500 km	GB155C	jittered	-0.289	-0.481	-0.094	0.991
GBI	500 km	GB057c	jittered	-0.283	-0.637	0.083	0.895
GBI	500 km	GB111C	jittered	-0.271	-0.466	-0.083	0.988
GBI	500 km	GB402	jittered	-0.264	-0.444	-0.084	0.99
GBI	500 km	GB138	jittered	-0.256	-0.477	-0.035	0.968
GBI	500 km	GB704drm	jittered	-0.238	-0.449	-0.024	0.963
GBI	500 km	GB296	jittered	-0.231	-0.476	0.017	0.932
GBI	500 km	GB553drmcC	jittered	-0.222	-0.469	0.026	0.923
GBI	500 km	GB074c	jittered	-0.221	-0.494	0.059	0.9
GBI	500 km	GB302c	jittered	-0.208	-0.464	0.05	0.902
GBI	500 km	GB501drm	jittered	-0.203	-0.441	0.034	0.915
GBI	500 km	GB522C	jittered	-0.2	-0.404	-0.007	0.946
GBI	500 km	GB951drmcC	jittered	-0.194	-0.413	0.021	0.924
GBI	500 km	GB415	jittered	-0.185	-0.371	0.001	0.944
GBI	500 km	GB309c	jittered	-0.18	-0.376	0.025	0.924
GBI	500 km	GB949m	jittered	-0.18	-0.394	0.038	0.907
GBI	500 km	GB129	jittered	-0.176	-0.367	0.018	0.928
GBI	500 km	GB432c	jittered	-0.175	-0.43	0.071	0.868
GBI	500 km	GB800EO	jittered	-0.172	-0.469	0.122	0.822
GBI	500 km	GB403	jittered	-0.168	-0.359	0.021	0.922
GBI	500 km	GB204	jittered	-0.155	-0.404	0.095	0.839
GBI	500 km	GB082cC	jittered	-0.15	-0.387	0.098	0.84
GBI	500 km	GB059	jittered	-0.147	-0.362	0.061	0.868
GBI	500 km	GB297	jittered	-0.145	-0.344	0.063	0.875
GBI	500 km	GB113	jittered	-0.145	-0.351	0.066	0.868
GBI	500 km	GB606m	jittered	-0.134	-0.32	0.054	0.875
GBI	500 km	GB099	jittered	-0.132	-0.32	0.063	0.865
GBI	500 km	GB558drm	jittered	-0.131	-0.305	0.041	0.887
GBI	500 km	GB135	jittered	-0.127	-0.284	0.021	0.91

GBI	500 km	GB500drm	jittered	-0.125	-0.345	0.093	0.819
GBI	500 km	GB139	jittered	-0.122	-0.319	0.084	0.835
GBI	500 km	GB147c	jittered	-0.121	-0.368	0.135	0.778
GBI	500 km	GB133C	jittered	-0.108	-0.377	0.175	0.734
GBI	500 km	GB123	jittered	-0.104	-0.315	0.102	0.788
GBI	500 km	GB049C	jittered	-0.1	-0.294	0.093	0.798
GBI	500 km	GB262c	jittered	-0.098	-0.349	0.145	0.735
GBI	500 km	GB421c	jittered	-0.097	-0.395	0.207	0.697
GBI	500 km	GB023c	jittered	-0.093	-0.356	0.163	0.717
GBI	500 km	GB107C	jittered	-0.089	-0.305	0.134	0.745
GBI	500 km	GB081	jittered	-0.089	-0.273	0.101	0.778
GBI	500 km	GB422c	jittered	-0.088	-0.372	0.191	0.694
GBI	500 km	GB299	jittered	-0.085	-0.301	0.143	0.729
GBI	500 km	GB158	jittered	-0.076	-0.26	0.12	0.741
GBI	500 km	GB068	jittered	-0.072	-0.294	0.153	0.698
GBI	500 km	GB480m	jittered	-0.068	-0.304	0.164	0.679
GBI	500 km	GB152	jittered	-0.067	-0.27	0.126	0.706
GBI	500 km	GB567drm	jittered	-0.066	-0.275	0.139	0.695
GBI	500 km	GB084c	jittered	-0.064	-0.263	0.139	0.698
GBI	500 km	GB250	jittered	-0.062	-0.311	0.184	0.655
GBI	500 km	GB702drmC	jittered	-0.06	-0.317	0.196	0.645
GBI	500 km	GB028	jittered	-0.06	-0.255	0.131	0.691
GBI	500 km	GB430c	jittered	-0.055	-0.302	0.196	0.638
GBI	500 km	GB069	jittered	-0.051	-0.236	0.129	0.674
GBI	500 km	GB301	jittered	-0.05	-0.258	0.173	0.643
GBI	500 km	GB260	jittered	-0.048	-0.198	0.107	0.694
GBI	500 km	GB286c	jittered	-0.045	-0.387	0.301	0.581
GBI	500 km	GB108	jittered	-0.017	-0.256	0.214	0.545
GBI	500 km	GB140	jittered	-0.016	-0.246	0.224	0.542
GBI	500 km	GB625mC	jittered	-0.013	-0.216	0.18	0.542
GBI	500 km	GB433c	jittered	-0.009	-0.264	0.246	0.523
GBI	500 km	GB431c	jittered	-0.008	-0.282	0.258	0.518
GBI	500 km	GB104	jittered	0.012	-0.216	0.239	0.535
GBI	500 km	GB065e	jittered	0.015	-0.214	0.245	0.542
GBI	500 km	GB298	jittered	0.016	-0.149	0.185	0.562
GBI	500 km	GB048	jittered	0.017	-0.17	0.212	0.556
GBI	500 km	GB334	jittered	0.024	-0.224	0.27	0.561
GBI	500 km	GB095C	jittered	0.026	-0.147	0.197	0.596
GBI	500 km	GB035	jittered	0.038	-0.188	0.262	0.608

GBI	500 km	GB127	jittered	0.046	-0.133	0.239	0.654
GBI	500 km	GB285cc	jittered	0.053	-0.444	0.569	0.566
GBI	500 km	GB620drmc	jittered	0.056	-0.152	0.263	0.666
GBI	500 km	GB136	jittered	0.061	-0.141	0.265	0.682
GBI	500 km	GB327	jittered	0.061	-0.113	0.245	0.708
GBI	500 km	GB263c	jittered	0.066	-0.181	0.323	0.663
GBI	500 km	GB852drmC	jittered	0.088	-0.139	0.325	0.728
GBI	500 km	GB253c	jittered	0.092	-0.142	0.317	0.739
GBI	500 km	GB323c	jittered	0.096	-0.168	0.357	0.722
GBI	500 km	GB322c	jittered	0.105	-0.29	0.49	0.665
GBI	500 km	GB622drmc	jittered	0.119	-0.113	0.344	0.8
GBI	500 km	GB550m	jittered	0.123	-0.071	0.318	0.846
GBI	500 km	GB109C	jittered	0.124	-0.112	0.358	0.803
GBI	500 km	GB950m	jittered	0.127	-0.102	0.363	0.808
GBI	500 km	GB118	jittered	0.127	-0.123	0.377	0.792
GBI	500 km	GB265	jittered	0.128	-0.078	0.336	0.837
GBI	500 km	GB103	jittered	0.128	-0.106	0.35	0.816
GBI	500 km	GB054c	jittered	0.148	-0.144	0.433	0.795
GBI	500 km	GB196C	jittered	0.15	-0.221	0.524	0.741
GBI	500 km	GB146	jittered	0.168	-0.052	0.388	0.889
GBI	500 km	GB149C	jittered	0.185	0	0.369	0.945
GBI	500 km	GB569drmcC	jittered	0.201	-0.118	0.53	0.841
GBI	500 km	GB304c	jittered	0.204	-0.044	0.453	0.905
GBI	500 km	GB850drm	jittered	0.208	0.058	0.347	0.987
GBI	500 km	GB332EON	jittered	0.21	0.059	0.366	0.982
GBI	500 km	GB559drm	jittered	0.213	0.085	0.347	0.993
GBI	500 km	GB623drmc	jittered	0.215	-0.013	0.449	0.931
GBI	500 km	GB556EO	jittered	0.22	-0.174	0.629	0.811
GBI	500 km	GB026	jittered	0.22	0.046	0.389	0.978
GBI	500 km	GB955drmc	jittered	0.228	0.086	0.378	0.992
GBI	500 km	GB401	jittered	0.229	-0.022	0.481	0.927
GBI	500 km	GB900EO	jittered	0.238	0.102	0.372	0.996
GBI	500 km	GB701drm	jittered	0.24	0.031	0.443	0.968
GBI	500 km	GB621drmc	jittered	0.244	0.044	0.448	0.972
GBI	500 km	GB052c	jittered	0.251	0.026	0.473	0.963
GBI	500 km	GB995F	jittered	0.26	0.115	0.415	0.995
GBI	500 km	GB954drmcC	jittered	0.262	0.076	0.442	0.988
GBI	500 km	GB132	jittered	0.272	0.034	0.497	0.969
GBI	500 km	GB098C	jittered	0.299	0.119	0.486	0.995

GBI	500 km	GB330c	jittered	0.299	-0.043	0.657	0.913
GBI	500 km	GB549drmc	jittered	0.301	0.117	0.473	0.995
GBI	500 km	GB273	jittered	0.302	0.08	0.519	0.985
GBI	500 km	GB568drm	jittered	0.315	0.109	0.523	0.992
GBI	500 km	GB046C	jittered	0.316	0.135	0.497	0.997
GBI	500 km	GB510m	jittered	0.316	0.09	0.554	0.985
GBI	500 km	GB551mC	jittered	0.318	0.109	0.523	0.993
GBI	500 km	GB331C	jittered	0.327	0.127	0.524	0.995
GBI	500 km	GB888drmcC	jittered	0.344	0.104	0.592	0.987
GBI	500 km	GB037	jittered	0.346	0.21	0.49	1
GBI	500 km	GB083c	jittered	0.356	0.165	0.544	0.998
GBI	500 km	GB051c	jittered	0.372	0.142	0.612	0.994
GBI	500 km	GB777drm	jittered	0.382	0.144	0.608	0.995
GBI	500 km	GB264c	jittered	0.386	0.18	0.604	0.998
GBI	500 km	GB117	jittered	0.391	0.222	0.554	1
GBI	500 km	GB116	jittered	0.399	0.215	0.573	1
GBI	500 km	GB563m	jittered	0.402	0.246	0.56	1
GBI	500 km	GB027	jittered	0.41	0.202	0.624	0.998
GBI	500 km	GB555m	jittered	0.417	0.199	0.631	0.999
GBI	500 km	GB156	jittered	0.431	0.241	0.63	1
GBI	500 km	GB105	jittered	0.448	0.228	0.658	0.999
GBI	500 km	GB953drmcC	jittered	0.45	-0.004	0.894	0.946
GBI	500 km	GB624drmc	jittered	0.471	0.233	0.714	0.999
GBI	500 km	GB410	jittered	0.49	0.295	0.678	1
GBI	500 km	GB853drm	jittered	0.512	0.277	0.737	1
GBI	500 km	GB408	jittered	0.513	0.281	0.74	1
GBI	500 km	GB131	jittered	0.515	0.286	0.742	1
GBI	500 km	GB020c	jittered	0.515	0.328	0.698	1
GBI	500 km	GB151	jittered	0.533	0.318	0.742	1
GBI	500 km	GB251m	jittered	0.548	0.318	0.765	1
GBI	500 km	GB198c	jittered	0.548	0.251	0.846	0.999
GBI	500 km	GB058C	jittered	0.552	0.275	0.818	1
GBI	500 km	GB324	jittered	0.559	0.379	0.735	1
GBI	500 km	GB556m	jittered	0.562	0.379	0.729	1
GBI	500 km	GB031	jittered	0.563	0.385	0.747	1
GBI	500 km	GB560drmcC	jittered	0.617	0.352	0.884	1
GBI	500 km	GB595m	jittered	0.645	0.46	0.842	1
GBI	500 km	GB177	jittered	0.645	0.471	0.818	1
GBI	500 km	GB130e	jittered	0.658	0.417	0.895	1

GBI	500 km	GB952drmcC	jittered	0.679	0.469	0.891	1
GBI	500 km	GB024e	jittered	0.68	0.407	0.95	1
GBI	500 km	GB557drm	jittered	0.69	0.508	0.867	1
GBI	500 km	GB990drmcC	jittered	0.698	0.476	0.916	1
GBI	500 km	GB047	jittered	0.706	0.52	0.904	1
GBI	500 km	GB203e	jittered	0.719	0.497	0.952	1
GBI	500 km	GB800m	jittered	0.794	0.581	1.012	1
GBI	500 km	GB021c	jittered	0.837	0.63	1.046	1
GBI	500 km	GB122	jittered	0.877	0.697	1.059	1
GBI	500 km	GB036	jittered	0.887	0.693	1.077	1
GBI	500 km	GB096C	jittered	0.913	0.72	1.116	1
GBI	500 km	GB552m	jittered	0.914	0.704	1.118	1
GBI	500 km	GB561drmc	jittered	0.924	0.716	1.133	1
GBI	500 km	GB053c	jittered	0.945	0.718	1.179	1
GBI	500 km	GB945m	jittered	0.98	0.797	1.166	1
GBI	500 km	GB991drmcC	jittered	1.011	0.793	1.223	1
GBI	500 km	GB409	jittered	1.18	0.938	1.43	1
GBI	500 km	GB124	jittered	1.229	1.024	1.438	1

Table S3.
List of variables and their source.

#	Variable	Description*	Aggregation per cell	Source
1	ln.richness	Logarithm of language and dialect richness	log(count)	Glottolog, v. 5.0 (4)
2	ln.tax.div	Logarithm of taxonomic diversity of attested languages and dialects	see ‘Methods’	Glottolog, v. 5.0 (4)
3	env.PC1	First PC of a PCA over variables 11-21 (see ‘Methods’), main loadings relate to temperature	–	–
4	env.PC2	Second PC of a PCA over variables 11-21 (see ‘Methods’), main loadings relate to terrain	–	–
5	ln.popd	Logarithm of population density (resolution: 5 arcminutes)	median	HYDE, v. 3.3 (5)
6	F.mean	Posterior estimates of Wright’s *F* coefficient (excess of homozygosity)	mean	GeLaTo (6)
7	feature	Linguistic feature identifier (features from TLI-statistical or GBI-statistical)	–	TLI/GBI (3)
8	area	AUTOTYP area (10 “continent-sized” areas) associated with observation	–	TLI/GBI (3)
9	grid.id	Identifier of a local observation unit	–	–
10	lon, lat	Coordinates (EPSG:8859)	–	–
11	precipitation	Seasonal variance of precipitation (resolution: 10 arcminutes).	median	WorldClim, v2 (7)
12	temp_mean	Mean annual temperature (resolution: 10 arcminutes).	median	WorldClim, v2 (7)
13	wettest	Precipitation in the wettest quarter (resolution: 10 arcminutes).	median	WorldClim, v2 (7)
14	warmest	Mean temperature of the warmest quarter (resolution: 10 arcminutes).	median	WorldClim, v2 (7)
15	n_warm_months	Months with mean temperature > 15°C (resolution: 10 arcminutes).	median	Derungs et al. 2018 (8)
16	grass	km2 of grassland/pasture (resolution: 5 arcminutes)	median	HYDE, v. 3.3 (5)
17	crop	km2 of cropland (resolution: 5 arcminutes)	median	HYDE, v. 3.3 (5)

18	elev	Highest elevation (resolution: 5 arcminutes)	max	Derungs et al. 2018 (8)
19	elevSD	Terrain ruggedness (sd of elevation, resolution: 5 arcminutes)	sd	Derungs et al. 2018 (8)
20	distOcean	Distance to ocean (resolution: 5 arcminutes)	median	Derungs et al. 2018 (8)
21	distRiver	Distance to river (resolution: 5 arcminutes)	median	Derungs et al. 2018 (8)

* Variables from HYDE (3.3) were extracted for the year 2000 CE. Variables from WorldClim2 figure the averages for the years 1970-2000.

Table S4.
Generalised additive mixed-effect models for estimating logit-transformed normalized entropy (div) using R notation. All numeric predictors are scaled.

Model	Description	Regression
m1	Null model	bf(div | se(div.sd, sigma = T)) ~ 1 + ln.richness + ln.tax.div + (1 + ln.richness + ln.tax.div | feature) + (1 + ln.richness + ln.tax.div | area) + (1 | grid.id) + t2(lon, lat) *
m2	Null model + environment	m1 + env.PC1 + env.PC2 + (env.PC1 + env.PC2 | feature) + (env.PC1 + env.PC2 | area)
m3	Null model + population density	m1 + ln.popd + (ln.popd | feature) + (ln.popd | area)
m4	Null model + genetic diversity	m1 + me(F.mean, F.sd) + (me(F.mean, F.sd) | feature) + (me(F.mean, F.sd) | area)
m5	Null model + environment + population density	m1 + env.PC1 + env.PC2 + ln.popd + (env.PC1 + env.PC2 + ln.popd | feature) + (env.PC1 + env.PC2 + ln.popd | area)
m6	Null model + environment + genetic diversity	m1 + env.PC1 + env.PC2 + me(F.mean, F.sd) + (env.PC1 + env.PC2 + me(F.mean, F.sd) | feature) + (env.PC1 + env.PC2 + me(F.mean, F.sd) | area)
m7	Null model + population density + genetic diversity	m1 + ln.popd + me(F.mean, F.sd) + (ln.popd + me(F.mean, F.sd) | feature) + (ln.popd + me(F.mean, F.sd) | area)
m8	Full model: null model + environment+ population density + genetic diversity	m1 + env.PC1 + env.PC2 + ln.popd + me(F.mean, F.sd) + (env.PC1 + env.PC2 + ln.popd + me(F.mean, F.sd) | feature) + (env.PC1 + env.PC2 + ln.popd + me(F.mean, F.sd) | area)

* The geographic locations of the grid cells (here: lon and lat) are indicated by their coordinates in projection EPSG:8859.

SI References

1. D. Lüdecke, M. S. Ben-Shachar, I. Patil, P. Waggoner, D. Makowski, performance: An R Package for Assessment, Comparison and Testing of Statistical Models. *Journal of Open Source Software* **6**, 3139 (2021).

2. B. Bickel, *et al.*, The AUTOTYP database. (2021). https://doi.org/10.5281/zenodo.4574513. Deposited 2 March 2021.

3. A. Graff, *et al.*, Curating global datasets of structural linguistic features for independence. *Sci Data* **12**, 106 (2025).

4. H. Hammarström, R. Forkel, M. Haspelmath, S. Bank, glottolog/glottolog: Glottolog database 5.0. Zenodo. https://doi.org/10.5281/zenodo.10804357. Deposited 11 March 2024.

5. K. Klein Goldewijk, History Database of the Global Environment 3.3. (2024). Available at: https://public.yoda.uu.nl/geo/UU01/94FNH0.html [Accessed 30 October 2024].

6. C. Barbieri, D. Blasi, R. Forkel, Eds., *GeLaTo* (Max Planck Institute for Evolutionary Anthropology, 2022).

7. S. E. Fick, R. J. Hijmans, WorldClim 2: new 1-km spatial resolution climate surfaces for global land areas. *International Journal of Climatology* **37**, 4302–4315 (2017).

8. C. Derungs, M. Köhl, R. Weibel, B. Bickel, Environmental factors drive language density more in food-producing than in hunter–gatherer populations. *Proceedings of the Royal Society B: Biological Sciences* **285**, 20172851 (2018).
